# Measuring β*‐*diversity with species abundance data

**DOI:** 10.1111/1365-2656.12362

**Published:** 2015-03-21

**Authors:** Louise J. Barwell, Nick J. B. Isaac, William E. Kunin

**Affiliations:** ^1^ Institute of Integrative and Comparative BiologyUniversity of LeedsLC Miall BuildingLeedsLS2 9JTUK; ^2^ Biological Records CentreCentre for Ecology and HydrologyMaclean Building, Benson Lane, Crowmarsh GiffordWallingfordOX10 8BBUK

**Keywords:** β‐diversity indices, community composition, differentiation, metrics, rank abundance distribution, similarity, simulated assemblage, spatial turnover

## Abstract

1. In 2003, 24 presence–absence β‐diversity metrics were reviewed and a number of trade‐offs and redundancies identified. We present a parallel investigation into the performance of abundance‐based metrics of β‐diversity.
2. β‐diversity is a multi‐faceted concept, central to spatial ecology. There are multiple metrics available to quantify it: the choice of metric is an important decision.
3. We test 16 conceptual properties and two sampling properties of a β‐diversity metric: metrics should be 1) independent of α*‐*diversity and 2) cumulative along a gradient of species turnover. Similarity should be 3) probabilistic when assemblages are independently and identically distributed. Metrics should have 4) a minimum of zero and increase monotonically with the degree of 5) species turnover, 6) decoupling of species ranks and 7) evenness differences. However, complete species turnover should always generate greater values of β than extreme 8) rank shifts or 9) evenness differences. Metrics should 10) have a fixed upper limit, 11) symmetry (β_*A*,*B*_ = β_*B*,*A*_), 12) double‐zero asymmetry for double absences and double presences and 13) not decrease in a series of nested assemblages. Additionally, metrics should be independent of 14) species replication 15) the units of abundance and 16) differences in total abundance between sampling units. When samples are used to infer β‐diversity, metrics should be 1) independent of sample sizes and 2) independent of unequal sample sizes. We test 29 metrics for these properties and five ‘personality’ properties.
4. Thirteen metrics were outperformed or equalled across all conceptual and sampling properties. Differences in sensitivity to species’ abundance lead to a performance trade‐off between sample size bias and the ability to detect turnover among rare species. In general, abundance‐based metrics are substantially less biased in the face of undersampling, although the presence–absence metric, β_sim_, performed well overall. Only β_Baselga R turn_, β_Baselga B‐C turn_ and β_sim_ measured purely species turnover and were independent of nestedness. Among the other metrics, sensitivity to nestedness varied >4‐fold.
5. Our results indicate large amounts of redundancy among existing β‐diversity metrics, whilst the estimation of unseen shared and unshared species is lacking and should be addressed in the design of new abundance‐based metrics.

## Introduction

Metrics of β‐diversity are widely used in ecological studies, but there is uncertainty about the degree of redundancy among the metrics available and the facets of β‐diversity being measured. Whittaker (1960, 1972) broadly defined β‐diversity as the spatial variation (turnover) in species composition and abundance between sampling units, whilst α‐diversity is the local diversity within a single sampling unit and γ‐diversity measures larger‐scale diversity.

The number of studies investigating β*‐*diversity has increased considerably in recent years (Koleff, Gaston & Lennon 2003a; Anderson *et al*. 2011). β‐diversity has been linked to the shape of the species–area curve (Harte *et al*. 1999), variance in species occupancy (McGlinn & Hurlbert 2012) and species’ spatial aggregation (Morlon *et al*. 2008). The distance–decay relationship (the increase in β‐diversity with geographical distance) is a critical component of three of the six unified theories of biodiversity reviewed by McGill (2010). Measures of β‐diversity in relation to environmental and spatial gradients have been used to unpick community assembly (Chase 2003) and drivers of global scale biodiversity patterns (Qian & Ricklefs 2007). Empirical measures of β‐diversity can be used to delineate biotic regions (Holt *et al*. 2013) and to inform the optimal configuration of reserves (Wiersma & Urban 2005). β‐diversity has been used to evaluate the landscape‐scale implications of farm management (Gabriel *et al*. 2006) and to assess the effects of environmental change on biotic homogenization (Baiser *et al*. 2012). Because γ‐diversity is entirely determined by the α*‐* and β‐components of diversity, empirical estimates of β‐diversity link biodiversity at local and regional scales (Smith 2010). Turnover in abundance also has important implications for ecosystem functioning and monitoring responses to disturbance (Balata, Piazzi & Benedetti‐Cecchi 2007).

A key distinction is between β‐diversity metrics that use presence–absence data and metrics that use species abundances (Anderson *et al*. 2011). Abundance data are clearly more information‐rich than presence–absence data, and this can change how we interpret spatial variation in assemblage structure (Cassey *et al*. 2008). For presence–absence metrics, the only visible differences between sites are in species identities. Abundance‐based measures detect more nuanced variation: we may observe all the same species at two sites, but those species may have different abundance ranks (the commonest species here may be rare there and vice versa). Even when the ranks are the same, evenness of abundances can vary (the common species can be more or less dominant). Consequently, we distinguish sensitivity to (i) species turnover, (ii) species richness differences (iii) rank abundance shifts and (iv) evenness differences as distinct components of β‐diversity. Abundance‐based indices may also be expected to be more robust to incomplete sampling (Beck, Holloway & Schwanghart 2013): stochastic differences in rare species are an artefact of undersampling, but abundance‐based metrics are less influenced by turnover of rare species than their presence–absence counterparts. Whilst abundance information makes our inferences about β‐diversity more powerful, it also introduces a source of subjectivity: we need to decide how to weight turnover in common and rare species.

Koleff, Gaston & Lennon (2003a) compared the performance of 24 presence–absence metrics of β‐diversity and identified a number of trade‐offs and redundancies among the presence–absence metrics available. Overall, they recommended β_*sim*_ (Lennon *et al*. 2001) as the best‐performing index. We are lacking an equivalent investigation into the performance and ‘personality’ of the many abundance‐based metrics available.

We test 16 conceptual properties that are important for an abundance‐based β‐diversity metric, whatever the application. Where applicable, we note the relationship between these properties and those previously described in the literature.

### Desirable properties

We make a distinction between conceptual and statistical properties. Conceptual properties (C1–C16) are intrinsic to the design of the metric (e.g. the use of abundance information and whether the metric has a fixed upper limit). Sampling properties (S1–S2) explore responses to undersampling: true differences between assemblages are confounded by imperfect detection, especially of rare species. We consider both conceptual and sampling properties as desirable when choosing a metric.

#### Independence of α‐diversity (C1)

β‐diversity should be independent of α*‐*diversity within assemblage pairs, so that the α*‐* and β‐ components of diversity can be partitioned (Jost 2007; Chase *et al*. 2011) and β‐diversity can be meaningfully compared between regions differing in α‐diversity. If α‐ and β‐diversities are independent, then pairs of assemblages with the same proportion of species turnover should have the same value of β‐diversity, regardless of whether α‐diversity within those assemblages is high or low. Legendre & De Cáceres (2013: property 10) test this property algebraically for 16 dissimilarity metrics. In P1, we consider an alternative where assemblage pairs have unequal species richness.

#### β is cumulative along a gradient of species turnover (C2)

When assemblages are positioned along an environmental gradient, species turnover will be directional. Koleff, Gaston & Lennon (2003a) call this property additivity. Species are gradually replaced as conditions change, so turnover between neighbouring pairs of assemblages is lower than between pairs that are farther apart. When samples A, B and C are positioned in sequence along such a gradient, summed β‐diversity between consecutive pairs of samples (β_A,B_ + β_B,C_) should equal the total β‐diversity between the end points of the gradient (β_A,C_). Metrics with disproportionate sensitivity to small amounts of turnover will lead to overestimates of cumulative β.

#### Similarity is probabilistic when assemblages are independently and identically distributed (C3)

When assemblages are independently drawn from within a larger, well‐mixed metacommunity, then similarity (i.e. 1‐β for metrics with an upper limit of 1) among multiple pairs of assemblages should be probabilistic. The expected similarity of assemblages A and C (1‐β_A,C_) is given by the product of similarities between A and B, and B and C, (1‐β_A,B_)*(1‐β_B,C_). Metrics that lack an upper limit cannot be converted to their similarity complement and so cannot be probabilistic.

#### Minimum of zero (C4)

Legendre & De Cáceres (2013: property 1) state that when comparing an assemblage to itself, β should always be zero, and when comparing two different assemblages, β should be equal to or greater than zero.

#### Fixed upper limit (C5)

Legendre & De Cáceres (2013: property 9) note that bounded metrics are easier to compare than unbounded ones. For example, the maximum value of β_Euclidean_ and β_Manhattan_ depends on the combined abundances of an assemblage pair, making it difficult to interpret the values of β when assemblage pairs have different numbers of individuals.

#### Monotonic increase with species turnover (C6)

β should be a strictly increasing monotonic function of the proportion of species in the first assemblage that are replaced by new species in the second assemblage; otherwise, it is not reflecting species turnover. A pair of assemblages in which 20% of assemblage A species are replaced by new species in assemblage B should have lower β‐diversity than an assemblage pair with 40% turnover. The property is closely related to the property described by Jost, Chao & Chazdon (2011) as monotonicity.

#### Monotonic increase with the decoupling of species ranks (C7)

An abundance‐based β‐diversity metric should be sensitive to the degree to which species ranks are decoupled between assemblage pairs (reflecting differences in the dominant and rare species). Therefore, β‐diversity should decrease monotonically with increased correlation between species ranks.

#### Monotonic increase with differences in evenness (C8)

Even if two sites have the same species, with the same rank order of abundances, they may still differ in evenness: the commonest species may dominate more in some sites than others. A good abundance‐based β‐diversity metric should increase monotonically as differences in evenness between sites grow larger. Properties C7 and C8 are two aspects of a property described as monotonicity to changes in abundance by Legendre & De Cáceres (2013: property 3).

#### β is lower for complete decoupling of species ranks than for complete species turnover (C9)

Consider a pair of assemblages in which all species are unshared and a second pair of assemblages in which all species are shared, but the rank abundances are reversed, such that the dominant species in assemblage A becomes the rarest in assemblage B and vice versa. The first pair of assemblages must be considered more different than the second pair.

#### β is lower for evenness differences than for complete species turnover (C10)

As an alternative scenario for abundance differences, consider a pair of assemblages in which all species are shared: in the first assemblage, the abundances are perfectly even and in the second assemblage, all species are singletons except the dominant species (e.g. extreme unevenness). Compare this to an assemblage pair where all species are shared. As above, the loss or gain of a species should always be deemed a more extreme difference than a shift in its abundance. Sites with no species in common should have the largest values of β (Legendre & De Cáceres 2013: property 5). Properties C9 and C10 describe two alternative scenarios in which this property should hold.

#### Symmetry (C11)

Legendre & De Cáceres (2013: property 2) and Koleff, Gaston & Lennon (2003a) note that the order in which two assemblages, A and B, are considered should not change the value of β for that pair (e.g. β_*A, B*_ *= *β_*B, A*_).

#### Double‐zero asymmetry (C12)

Legendre & De Cáceres (2013: property 4) argue that the absence of a species from both assemblages does not indicate resemblance between the two assemblages in the way that shared presences do: double absences contain no information about the distance in ecological niche space. Consequently, the addition of zero abundances to both assemblages should not change the value of β*,* whilst the addition of shared presences should lower the value of β.

#### β does not decrease in a series of nested assemblages (C13)

Metrics vary in how they respond to nestedness. However, β should never decrease when species richness differences increase, as the addition of unique species should not increase similarity (Legendre & De Cáceres 2013: property 6).

#### Independence of species replication (C14)

When all species in both the assemblages being compared are duplicated, the value of β should remain constant. This becomes important when identical subsets of an assemblage are pooled (Jost, Chao & Chazdon 2011; Legendre & De Cáceres 2013: property 7).

#### Independence of units of abundance (C15)

When comparing β among regions differing in productivity or the units used to measure abundance, metrics that are sensitive to the total abundance in an assemblage pair will be inappropriate. Legendre & De Cáceres (2013: property 8) call this property invariance to measurement units.

#### Independent of differences in abundance (C16)

This property was described as invariance to the total abundance in each assemblage by Legendre & De Cáceres (2013: property 11) and density invariance by Jost, Chao & Chazdon (2011). It is designed to identify metrics that are mathematically dependent on differences in abundance between sampling units. C15 and C16 differ from undersampling in that there is no stochasticity.

#### Unbiased by undersampling (S1)

In all previous simulations, we have assumed our simulated assemblages represent the ‘true’ composition. However, β‐diversity is usually estimated from samples, which generates differences in richness and abundances as a sampling artefact (Chao *et al*. 2005, 2006). A good β‐diversity metric should remain constant as the sample size decreases.

#### Unbiased by unequal sampling effort (S2)

Differences in sample size can also inflate β‐diversity due to imperfect detection of rare species. A good β‐diversity metric should remain constant with increasing difference in sample sizes.

### Personality properties

In addition to the desirable properties identified above, β‐diversity metrics may differ in other respects that are worthy of note. We term this the ‘personality’ of the metrics and their importance will depend on the ecological question concerned.

#### Sensitivity to nestedness (P1)

For presence–absence metrics, Koleff, Gaston & Lennon (2003a) distinguish ‘narrow‐sense’ metrics, which measure purely species turnover, from ‘broad‐sense’ metrics, which measure both species turnover and differences in species richness. We may want a β‐diversity metric to reflect differences in richness, as these will mean that one site will have species that are absent in another. On the other hand, we may want the value of β to measure purely species turnover, especially if we are comparing β‐diversity between regions with different species richness. This differs from the test in C1 (independence of differences in α‐diversity): in C1, each pair of assemblages we compare has an equal number of species. Here, species richness differs between the two assemblages we compare.

#### Relative sensitivity to nestedness and turnover components of β (P2)

We test two metrics (β_Bray‐Curtis_ and β_Ruzicka_) that can be additively partitioned into independent nestedness and turnover components (Baselga 2013; Podani, Ricotta & Schmera 2013; Legendre 2014). For metrics that cannot be deconstructed, it is useful to compare the value of β for complete turnover to that for extreme nestedness to estimate the relative sensitivity to these components.

#### Relative weighting of species turnover and abundance differences (P3 and P4)

We have identified two ways in which species abundances can vary between assemblages: decoupling of species ranks and differences in evenness. The relative weighting of these components and species turnover is a useful property to quantify. The ideal weighting is somewhat subjective (provided that β‐diversity is less for extreme differences in abundance than for turnover of a species, see C9 and C10, above).

#### Relative sensitivity to turnover of rare versus common species (P5)

There is scope for variation in how common versus rare species contribute to β. One reason for investigating this is the occupancy–abundance relationship (ONR). Positive ONRs are nearly ubiquitous (Brown 1984) and reflect that rare species are generally more range restricted and so more likely to be turned over than are locally abundant (and more widespread) species.

Here, we manipulate the composition and structure of hypothetical assemblages and apply 29 β‐diversity metrics to the resulting assemblage pairs. Each metric is evaluated against 18 desirable properties (C1–C15 and S1–S2) to generate a score card, which we use to identify the best‐performing abundance‐based β‐diversity metrics. We then explore how personality properties may affect the choice of metric for different ecological applications.

## Materials and methods

### β*‐*diversity metrics

In total, we evaluated 24 abundance‐based metrics and five presence–absence metrics (Appendix S1, Supplementary Information). All metrics are expressed so that higher values of β indicate more differentiation (1‐β for similarity metrics). For comparability, metrics were rescaled relative to the maximum value obtained in each set of simulations, before calculating scores.

### Hypothetical species assemblages

Abundance differences in our hypothetical assemblages were modelled using the log series distribution (Fisher, Corbet & Williams 1943) using the function *fisher.ecosystem* in R package ‘untb’ (Hankin 2007). Our conclusions would be qualitatively identical using other commonly used models of the species abundance distribution (McGill 2010). A hypothetical species assemblage with 100 species and 10 000 individuals was used as the starting assemblage for all simulations.

### Evaluation of properties

For β‐diversity metrics that have been previously implemented in R, the functions *vegdist* and *d* and *adipart* in R package ‘vegan’ v.2.0‐5 (Oksanen *et al*. 2013) were used to calculate β‐diversity. Formulae for the remaining metrics can be found in Appendix S1. Each of our properties was assessed by exploring how measured β‐diversity covaried with a test‐specific parameter, describing some aspect of assemblage structure. We manipulated the starting assemblage according to the specific rules for each test. Each simulation described below was run 10 000 times at each unique combination of the test‐specific parameter and proportion species turnover, *t* = 0, 0·2, 0·4, −0·6, 0·8 and 1·0, to obtain median β for that combination. All simulations were carried out in R v.3.0.3 (R Core Team 2014). Formulae for evaluating β‐diversity metrics for each of the properties can be found in Appendix S2.

#### Independence of α‐diversity (C1)

Fisher's α of assemblages was manipulated using the function *fisher.ecosystem* in R package ‘untb’ (Hankin 2007). The expected number of individuals was fixed at *N* = 10 000, whilst manipulating the number of expected species, *S*, to generate a series of assemblages with *S* = 300, 250, 200, 150, 100, 80, 60, 40, 20 and 10. Fisher's α was estimated for each assemblage. For each α‐diversity:turnover combination, we calculated error as the difference between the median β‐diversity at each level of α and the median β‐diversity when α was highest (*S* = 300): dependence on α*‐*diversity was measured as the root‐mean‐squared error (RMSE).

#### β is cumulative along a gradient of species turnover (C2)

In each simulation, three assemblages, A, B and C, were generated according to the following rules: a proportion of species, *t*, in assemblage A were randomly selected to be turned over in assemblage B (*t* = 0, 0·1, 0·2, 0·3, 0·4 and 0·5). Of the species in assemblage B, the same proportion was turned over in assemblage C, with the condition that species shared between assemblages A and B were *g* times more likely to be turned over in assemblage C than species unique to assemblage B, where *g* is a test‐specific parameter which we manipulate to simulate different strengths of directional species turnover (*g* = 1, 5, 10, 50, 100, 500 or 1000). At each turnover:gradient combination, we calculated error as the difference between observed β‐diversity for assemblages A and C (β_*A,C*_) and the value predicted if the metric was cumulative (β_*A,B*_ + β_*B,C*_): departure from cumulative β was evaluated as the RMSE.

#### Similarity is probabilistic when assemblages are distributed independently and identically in space (C3)

In each simulation, three assemblages, A, B and C, were generated according to the following rules: a proportion, *p* (*p = *0–1 in increments of 0·2), of the species in assemblage 1 were randomly selected to be conserved in assemblage 2. This process was repeated with the species in assemblages 1 and 2 (with the same value of *p*) to obtain the third assemblage. Species lost from assemblage A can reappear in assemblage C, as we would expect in independent samples drawn from a well‐mixed species pool, but entirely novel species can also appear in assemblage C. In each simulation, we calculated error as the difference between observed similarity for assemblages A and C (1−β_*A,C*_) and the similarity predicted if the metric is probabilistic (1−β_*A,B*_)(1−β_*B,C*_): departure from probabilistic similarity was evaluated as the RMSE.

#### Minimum of zero (C4)

The starting assemblage was manipulated to generate assemblage pairs with increasing differences in species turnover, *t*; decoupling of species ranks, *r*; and evenness differences, *ΔE*. Methods for these simulations can be found in C7 and C8. Two behaviours were tested: (i) β is zero for identical assemblages and (ii) β is greater than or equal to zero when assemblages are different, either because of species turnover, decoupling of species ranks or evenness differences. The metric was scored as TRUE if both qualities were met.

#### Fixed upper bound (C5)

This property was evaluated as TRUE/FALSE by applying equation 8 and then equation 3 in Legendre & De Cáceres (2013: property 9) to calculate the upper limit of a metric, using a pair of assemblages with no shared species.

#### Monotonic increase with species turnover (C6)

A series of assemblages with increasing species turnover was generated by randomly selecting a proportion of species (*t* = 0–1 in increments of 0·2) in the starting assemblage and assigning them a new identity in the new assemblage. Metrics were scored as TRUE if each consecutive increase in species turnover generated an increase in median β.

#### Monotonic increase with decoupling of species ranks (C7)

A series of assemblages with increased decoupling of species ranks was generated by determining species ranks in the new assemblage partially by the ranks in the starting assemblage and partially at random (*r* = +1·0 (a perfect positive correlation between ranks) to −1·0 (a perfect negative correlation) in increments of 0·1). Metrics were scored as TRUE if each incremental decrease in *r*, generated an increase in median β at a given level of species turnover.

#### Monotonic increase with differences in evenness (C8)

In the starting assemblage for this test all except the dominant species have just one individual (extreme unevenness). A series of assemblages with increasing evenness differences was generated by redistributing individuals from the dominant species among the other 99 species: the probability of being allocated to each species was determined by raising the abundances in a Fisher's log series distributed assemblage to a power, *b* = 0·2, 0·4, 0·6, 0·8, 1·0, 1·2, 1·4, 1·6, 1·8 2·0, 4·0, 6·0 and 8·0. These values were chosen to generate assemblages with both more and less evenness relative to a Fisher's log series distribution. Metrics were scored as TRUE if each incremental increase in *ΔE* led to an increase in median β.

#### β under extreme decoupling of species ranks *<* β when species turnover is complete; β under extreme evenness differences *<* β when species turnover is complete (C9 and C10)

The turnover of a species should be weighted greater than a change in abundance. Metrics were scored as TRUE for these two properties if median β is lower for extreme decoupling of species ranks (*r* = −1) and extreme evenness differences (*ΔE* = 0·97) than for complete species turnover (*t* = 1). The relative weighting of abundance differences and species turnover also has a personality component (see P3 and P4).

#### Symmetry (C11)

Symmetry was tested by reversing the order in which assemblages A and B were given to a metric. This was tested for assemblage pairs with multiple levels of species turnover, *t;* decoupling of species ranks, *r*; and evenness differences, *ΔE*. A metric was scored as TRUE if β_*A,B*_ = β_*B,A*_ in all simulations.

#### Double‐zero asymmetry (C12)

We generated a series of eleven assemblage pairs, the first with no double zeros and then consecutively adding up to 10 double zeros to the assemblage pair. This was repeated, but adding double presences of equal abundance. Abundances in each simulation were chosen at random from within the starting assemblage. Two behaviours were tested: (i) β does not change with the addition of double zeros and (ii) β decreases with the addition of double presences. Metrics were scored as TRUE if both conditions were met.

#### β does not decrease in a series of nested assemblages (C13)

A series of nested assemblages was generated by randomly selecting a number of species to be lost from the starting assemblage (*S = *0–90 in increments of 10). Metrics were scored as TRUE if each incremental increase in species loss led to an increase in median β.

#### Independence of species replication (C14)

A series of 10 assemblage pairs with all species replicated *x* times at six levels of species turnover, *t*, was used to simulate the effect of pooling identical subsets of unshared species. At each combination of *x* (in 1–10) and *t*, error was calculated as the difference between median β in one identical subset and when *x* identical subsets were pooled. Metrics were scored as the RMSE.

#### Independent of the units of abundance (C15)

Following the method in Legendre & De Cáceres (2013), we test this property by generating a series of assemblage pairs in which the abundances in both assemblages are multiplied by a constant factor (*cc* = 1–10). Error was calculated as the difference between median β in the starting assemblage pair (*cc *= 1) and between median β at each combination of *cc* and species turnover, *t*. Metrics were scored as the RMSE.

#### Independence of differences in abundance (C16)

We test this property by generating a series of assemblage pairs in which the abundances in *one* assemblage are multiplied by a constant factor, (*c *= 1–10). At each *c*:turnover combination, error was calculated as the difference between median β at each value of *c* and median β in the starting assemblage pair, (*c *= 1). Metrics were scored as the RMSE.

The following two properties test the behaviour of metrics when samples are used to infer β‐diversity.

#### Independence of sample size (S1)

For a series assemblage pairs with different levels of turnover, *t*, both assemblages were randomly sampled, without replacement, to generate a series of assemblage pairs with equal sample sizes of *N* = 10 000 (fully censused), 9000, 8000, 7000, 6000, 5000, 4000, 3000, 2000, 1000, 500, 200, 100, 50, 20 and 10. For each sample size:turnover combination, error was calculated as the difference between median β‐diversity at sample size *N* and median β‐diversity in a fully censused assemblage: dependence on sample size was measured as the RMSE.

#### Independence of unequal sample sizes (S2)

For a series of assemblage pairs with different levels of turnover, *t*, one assemblage in each pair was randomly sampled, without replacement, whilst the other was fully sampled to generate sample size differences of *ΔN *= 0, 1000, 2000, 3000, 4000, 5000, 6000, 7000, 8000, 9000, 9500, 9800, 9900, 9950, 9980 and 9990. As above, for each *ΔN*:turnover combination, we calculated error as the difference between the median β‐diversity at sample size difference *ΔN* and median β‐diversity when both assemblages were fully censused (*ΔN *= 0): dependence on unequal sample size was measured as the RMSE.

#### Sensitivity to nestedness (P1)

To generate ten assemblages with differences in species richness, *ΔS*, we randomly selected *S* species (see C13) to be lost from the starting assemblage. For each species loss:turnover combination, we calculated error as the difference between the median β‐diversity for *S* and median β‐diversity when species richness was equal (*S* = 0): sensitivity to nestedness was measured as the RMSE.

#### Relative sensitivity to nestedness and turnover (P2)

This property was measured as the ratio of β under extreme nestedness but no turnover (*ΔS* = 90, *t* = 0) and the value for complete species turnover but no species loss (*t = *1, *ΔS = *0).

#### Relative sensitivity to abundance differences and species turnover (P3 and P4)

We calculated β under extreme decoupling of species ranks (*r* = −1), and extreme differences in evenness (*ΔE* = 0·97), using simulated assemblages from C7 and C8. These values were expressed as a proportion of the value of median β under complete species turnover, *t *= 1.

#### Relative sensitivity to turnover in rare versus common species (P5)

We turned over a single species in the starting assemblage, from the dominant (1450 individuals) to the rarest species (1 individual) and recorded the value of β for each. Relative sensitivity to rare and common species was evaluated as the ratio between β when the rarest species was turned over to β when the dominant species was turned over.

In order to investigate redundancy and complementarity among the 29 metrics, a principal component analysis was performed using all quantitatively measured properties, using the function *prcomp* in R version 3.0.3 (R Development Core Team, 2014). We also investigate which of the metrics are Pareto‐dominated, that is, those metrics that are outperformed or equalled across all desirable properties.

## Results

We have scored the performance of 29 metrics for 16 conceptual and two sampling properties (Table 1). In addition, a further five personality tests have enabled us to identify more subjective variation in metrics’ behaviour (Table 2). The results of all simulations are presented in Appendix S3.

**Table 1:** Scorecard for 29 β*‐*diversity metrics against the 16 conceptual and two sampling properties described in the text. Metrics are ordered by number of TRUES and, when equal, by the mean of quantitative scores. Note this weights qualitative properties greater than quantitative properties, such that metrics with one or two fails drop down the scorecard. Metrics have an ideal score of TRUE (T) for qualitative properties and 0 for quantitative properties. C4, C6 and C11 were TRUE for all metrics and scores are not shown

Metric	Conceptual properties													Sampling properties		Performance summary		
	C1	C2	C3	C5	C7	C8	C9	C10	C12	C13	C14	C15	C16	S1	S2	#T	# F	Mean of quantitative scores
Morisita	0·0757	0·0197	0·0047	T	T	T	T	T	T	T	0·0030	0·0038	0·0027	0·0159	0·0036	8	0	0·0161
Horn	0·0294	0·0331	0·0024	T	T	T	T	T	T	T	0·0009	0·0016	0·0007	0·1359	0·0357	8	0	0·0300
Morisita‐Horn	0·0763	0·0200	0·0048	T	T	T	T	T	T	T	0·0030	0·0037	0·0026	0·1356	0·0899	8	0	0·0420
Jost Simpson	0·0826	0·0642	0·0037	T	T	T	T	T	T	T	0·0030	0·0043	0·0026	0·1157	0·0694	8	0	0·0432
Renkonen^a^	0·0294	0·0331	0·0024	T	T	T	T	T	T	T	0·0009	0·0016	0·0007	0·1690	0·1433	8	0	0·0476
Kulczynski^a^	0·0294	0·0331	0·0024	T	T	T	T	T	T	T	0·0009	0·0016	0·2292	0·1690	0·2235	8	0	0·0861
Bray‐Curtis^a^	0·0294	0·0331	0·0024	T	T	T	T	T	T	T	0·0009	0·0016	0·3916	0·1690	0·4151	8	0	0·1304
Canberra	0·0000	0·1584	0·0170	T	T	T	T	T	T	T	0·0000	0·0000	0·3433	0·2260	0·3699	8	0	0·1393
Ružička	0·0312	0·1166	0·0153	T	T	T	T	T	T	T	0·0010	0·0015	0·3966	0·1881	0·3902	8	0	0·1426
bBaselga B‐C turn	0·0294	0·0331	0·0024	T	T	T	T	T	T	F	0·0009	0·0016	0·0007	0·1690	0·0031	7	1	0·0300
NESS	0·0062	0·0351	0·0014	T	T	T	T	T	F	T	0·0137	0·0010	0·0009	0·1431	0·0945	7	1	0·0370
bBaselga R turn	0·0312	0·1166	0·0153	T	T	T	T	T	T	F	0·0010	0·0015	0·0006	0·1881	0·0032	7	1	0·0447
bPodani B‐C turn^a^	0·0294	0·0331	0·0024	T	T	T	T	T	T	F	0·0009	0·0016	0·3916	0·1690	0·4156	7	1	0·1305
bPodani R turn^a^	0·0312	0·1166	0·0153	T	T	T	T	T	T	F	0·0010	0·0015	0·4556	0·1881	0·4736	7	1	0·1604
sim	0·0000	0·0547	0·0000	T	F	F	T	T	T	T	0·0000	0·0000	0·0000	0·1485	0·0026	6	2	0·0257
Classic Sørensen^a^	0·0000	0·0547	0·0000	T	F	F	T	T	T	T	0·0000	0·0000	0·0000	0·1618	0·2299	6	2	0·0558
Classic Jaccard^a^	0·0000	0·1584	0·0170	T	F	F	T	T	T	T	0·0000	0·0000	0·0000	0·1854	0·2404	6	2	0·0752
Jost Shannon	0·0302	0·0482	0·0079	T	T	T	T	T	F	F	0·0009	0·0017	0·2529	0·1272	0·3459	6	2	0·1019
Chao Sørensen	0·0300	0·0330	0·0034	T	F	F	T	T	F	T	0·0019	0·0023	0·0015	0·0481	0·0849	5	3	0·0256
Chao Jaccard	0·0300	0·1160	0·0155	T	F	F	T	T	F	T	0·0014	0·0017	0·0014	0·0645	0·1038	5	3	0·0418
Lande Shannon^a^	0·0294	0·0331	F	F	T	T	T	T	F	T	0·0009	0·0016	0·2322	0·1359	0·1462	5	3	0·0828
CYd	0·1280	0·1400	F	F	T	F	T	T	T	T	0·0003	0·1703	0·1324	0·2091	0·2528	5	3	0·1476
Lande Simpson	0·2586	0·0200	F	F	T	T	T	F	F	T	0·4663	0·0037	0·2905	0·0614	0·0446	4	4	0·1636
Binomial	0·4092	0·0547	F	F	F	T	T	T	F	F	0·3233	0·0000	0·1157	0·2704	0·1911	3	5	0·1949
Gower^a^	0·0000	0·0577	0·1350	T	F	F	F	F	F	T	0·0000	0·0000	0·5054	0·4137	0·4602	3	5	0·1965
Manhattan^a^	0·0294	0·0331	F	F	F	T	T	T	F	F	0·3244	0·3244	0·2669	0·4458	0·2542	3	5	0·2397
alt. Gower^a^	0·1802	0·1022	F	F	F	T	F	F	T	T	0·0012	0·3258	0·3457	0·4232	0·3334	3	5	0·2445
Av. Euclidean^a^	0·2178	0·1786	F	F	F	T	F	F	T	T	0·0022	0·3479	0·3646	0·4762	0·2888	3	5	0·2680
Euclidean^a^	0·2625	0·1393	F	F	F	T	T	F	F	T	0·3168	0·3775	0·3586	0·5203	0·2670	3	5	0·3203

a Pareto‐dominated.

b Partitioned turnover component of β.

**Table 2:** Summary of scores for personality and sampling properties among 29 beta‐diversity metrics. Properties P1–P5 are described in the text

Metric	Personality properties				
	P1	P2	P3	P4	P5
Morisita	0·2862	0·8538	0·9940	0·9798	0·0000
Horn	0·1989	0·6046	0·9012	0·9195	0·0007
Morisita‐Horn	0·2861	0·8541	0·9940	0·9798	0·0000
Renkonen	0·3305	0·9150	0·9544	0·9801	0·0007
Jost Simpson	0·2631	0·7453	0·9880	0·9604	0·0000
Kulczynski	0·1619	0·4575	0·9544	0·9801	0·0007
Bray‐Curtis	0·2678	0·8433	0·9544	0·9801	0·0007
Canberra	0·2759	0·9000	0·7979	0·9802	1·0000
Ružička	0·2825	0·9150	0·9767	0·9900	0·0008
Baselga B‐C turn^a^	0·0198	0·0000	0·9544	0·9801	0·0007
NESS	0·2424	0·7749	0·9634	0·9289	0·0000
Baselga R turn^a^	0·0219	0·0000	0·9767	0·9900	0·0008
Podani B‐C turn^a^	0·2672	0·0000	0·9543	0·9801	0·0007
Podani R turn^a^	0·3154	0·0000	0·9767	0·9900	0·0008
sim	0·0000	0·0000	0·0000	0·0000	1·0000
Classic Sørensen	0·2574	0·8182	0·0000	0·0000	1·0000
Classic Jaccard	0·2759	0·9000	0·0000	0·0000	1·0000
Jost Shannon	0·1675	0·1807	0·8676	0·8915	0·0007
Chao Sørensen	0·2665	0·8406	0·0000	0·0000	0·0000
Chao Jaccard	0·2819	0·9134	0·0000	0·0000	0·0000
Lande Shannon	0·1996	1·4297	0·9012	0·9195	0·0007
CYd	0·2582	0·9001	0·6221	0·6243	0·1682
Lande Simpson	0·1121	5·0896	0·9940	48·5149	0·0000
Binomial	0·1823	0·4500	0·3264	0·4599	1·0000
Gower	0·2759	0·9000	1·0000	1·0000	1·0000
Manhattan	0·1860	0·4575	0·9544	0·9801	0·0007
alt. Gower	0·2154	0·9150	1·9088	1·9602	0·0007
Av. Euclidean	0·2430	0·9766	1·4099	9·8504	0·0007
Euclidean	0·2303	0·6905	0·9970	6·9653	0·0007

a Partitioned turnover component of β.

### Conceptual and sampling properties

All 29 metrics satisfied properties C4, C6 (minimum of zero and positiveness, monotonic increase with species turnover: Fig. S4) and C11 (symmetry). We use the remaining properties to discriminate between the performances of metrics. Thirteen metrics were Pareto‐dominated (Tables 1, S5). We focus on the metrics that performed best against the conceptual and sampling properties and consider their contrasting strengths and weaknesses.

Nine metrics passed all qualitatively scored tests (β_Morisita_, β_Horn_, β_Morisita‐Horn_, β_Jost Simpson_ β_Renkonen_, β_Kulczynski_, β_Bray‐Curtis_, β_Canberra_ and β_Ružička,_: C5–C13, Table 1). The presence–absence metrics β_sim_, β_Classic Jaccard_ and β_Classic Sørensen_ failed only C7 and C8 (monotonic increase with decoupling of species ranks and evenness differences: Figs S5 and S6), as such measures, by definition, are insensitive to differences in abundance. All abundance‐based metrics became less sensitive to abundance differences as the species turnover between assemblages became more extreme (Figs S5 and S6).

Across all quantitative tests, β_Morisita_ obtained the best mean score. The presence–absence metric, β_sim,_ performed best or joint best for six of the eight quantitative conceptual and sampling properties, with the exception of C2 (β is cumulative) and S1 (independence of sample size). β_Morisita_ was the most robust metric to undersampling, performing best when both assemblages were undersampled (S1) and second best under unequal sample sizes (S2). β_sim_ was best for S2, but performed poorly for S1 (Figs S2 and S12: Table 1). β_Canberra_ scored equally highly with β_sim,_ β_Classic Sørensen_ and β_Classic Jaccard_ for C1 (independence of α‐diversity: Fig. S1), C14 (independence of species replication: Fig. S7) and C15 (independence of measurement units: Fig. S8), but performed poorly on C2 (β is cumulative: Fig. S2), C3 (similarity is probabilistic: Fig. S3) and C16 (independence of differences in abundance: Fig. S9) and for both sampling properties (S1 and S2). β_Binomial_ was joint best for C15 (independence of measurement units: Fig. S8), but performed poorly for all other quantitative properties. β_Horn_ and β_Renkonen_ performed relatively well across all quantitative properties, but were never best for any property.

In sampling simulations S1 and S2 (Table 1; Figs S12 and S13), most presence–absence metrics were positively biased by undersampling, with the exception of β_Chao Sørensen_ and β_Chao Jaccard_ which have a correction for undersampling.

### Personality properties

With the exception of β_sim_ and the partitioned turnover components of β_Bray‐Curtis_ and β_Ružička_, all metrics were at least somewhat sensitive to nestedness (P1), although there were fourfold differences in the degree of sensitivity to species richness differences (P2, Table 2).

The relative weighting of abundance differences and turnover varied substantially among abundance‐based metrics (Table 2). With the exception of β_Gower_ β_alt. Gower_, β_Av. Euclidean_, β_Lande Simpson_ and β_Euclidean_, metrics were more sensitive to species turnover than differences in abundance (P3: decoupling of species ranks, P4: differences in evenness, Figs S5 and S6).

The relative sensitivity to turnover in rare versus common species (P5) varied substantially among metrics from equal weighting of rare and common species (all presence–absence metrics) to metrics that had a negligible response to turnover in rare species (β_*Morisita*_: Fig. S11).

**Figure 1:**
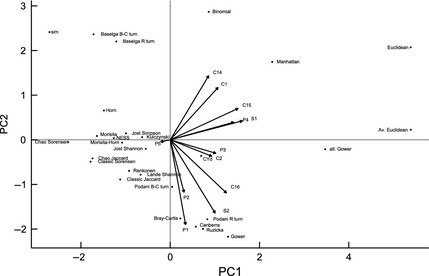
Biplot of the first two principal components axes of the scores of 29 β*‐*diversity metrics based on quantitative scores for properties C1–C2, C14–C16, S1–S2 and P1–P5. Four partitioned turnover components are also shown, using the partitioning methods proposed by Baselga (2013) and Podani, Ricotta & Schmera (2013). Together, PC1 and PC2 explain 52% of variation in scores.

A principal component analysis revealed substantial redundancy among the 29 metrics investigated (Fig. 1).

## Discussion

Our results identify a number of trade‐offs in performance, consider redundancy and complementarity among existing metrics and suggest areas to be addressed in the design of new metrics.

In choosing a metric, we suggest that our desirable properties will provide a useful primary filter in choosing a metric. We focus on the best‐performing metrics in Table 1, but other metrics may still be useful if the relative weighting of the desirable properties is changed, or if personality properties or additional properties, untested here, become important. Our personality properties highlight two additional sources of variation which may further filter the appropriate metrics for some applications: (i) sensitivity to rare species and (ii) sensitivity to nestedness. Our results indicate the first of these is traded‐off with performance for sampling properties (Fig. S16).

The most extreme example of this trade‐off is β_Morisita_, which is the most independent of sample size (Fig. S10), at the expense of being almost completely insensitive to turnover in rare species (Fig. S13). β‐diversity metrics fall along a continuum in terms of sensitivity to rare species. β_Classic Sørensen_ is conceptually linked to species richness metrics of α‐diversity such that rare and dominant species are weighted equally. β_Horn_ relates to Shannon entropy: species are weighted by their relative abundance. β_Morisita_ is linked to the Gini‐Simpson index of α‐diversity (Jost 2007): rare species contribute little to the final value of these metrics. Consequently, β_Morisita_ performs well, even with the very partial samples that ecologists usually work with, because the missing rare species in small samples have a negligible effect on the value of β. This may be important: the emphasis β_Morisita_ places on common species is suitable when shifts in dominance are of interest (e.g. when linking diversity to ecosystem function), but will be less appropriate when patterns of turnover in rare species of particular interest (e.g. complementarity of reserve networks: Wiersma & Urban (2005)). Unfortunately, those metrics that *are* sensitive to turnover in rare species are, consequently, less robust in the face of undersampling.

In general, our results suggest that when insensitivity to sample size (S1 and S2), sensitivity to turnover of individuals (C7 and C8) and/or cumulative β (C2) are priorities, β_Morisita_ should be favoured. When turnover in rare species is important and undersampling is not severe, the presence–absence metric, β_sim_, is favoured due to superior performance in terms of independence of α‐diversity (C1), probabilistic similarity (C3), independence of species replication (C14), measurement units (C15) and differences in abundance (C16). However, β_Morisita_ is almost completely independent of sample size (S1), whilst β_sim_, β_Classic Sørensen_ and β_Classic Jaccard_ are eleventh, twelfth and eighteenth. This is consistent with predictions that presence–absence metrics are more sensitive to sample sizes.

An example of where our results have implications for existing studies of β‐diversity is in the spatial scaling of β‐diversity. Studies using presence–absence metrics have shown that β*‐*diversity decreases with the spatial grain of samples (McGlinn & Hurlbert 2012; Barton *et al*. 2013). One reason for this is statistical: the probability of a rare species being turned over increases at finer grains (Keil *et al*. 2012) both because rare species are range‐restricted and because fine‐grain samples have (almost by definition) much smaller sample sizes of individuals than do coarse‐grain samples. By contrast, common species are usually more widespread than rare species and much less likely to be turned over at fine grains. The trade‐off we have noted between robustness to undersampling and sensitivity to rare species thus becomes relevant here: those metrics which weight rare species turnover highly (including all presence/absence measures) will likely find β shifting with scale. It follows that abundance‐based metrics, particularly those disproportionately influenced by dominant species, will likely be less scale dependent than presence–absence metrics (Fig. S15).

A second consequence of this trade‐off is that metrics that are insensitive to turnover in rare species will also return very low values of beta under a positive occupancy–abundance relationship (Fig. S14), a pattern that is near ubiquitous. Specialist applications focussing on rare species may need to use metrics that are less robust to undersampling but, consequently, will require larger sample sizes to observe the rarer species: no abundance‐based metric is able to account for unseen shared species (i.e. abundance‐based equivalents of β_*Chao Sørensen*_ and β_*Chao Jaccard*_).

Another potential filter of metrics is sensitivity to nestedness (P1). There are circumstances when the partitioning of the nestedness and turnover components will be a priority when choosing a metric. First, metrics measuring purely species turnover address methodological issues associated with species richness gradients (e.g. latitudinal gradients: Koleff, Lennon & Gaston 2003b). Moreover, patterns of nestedness and turnover are likely to emerge as a result of different processes: distinguishing these patterns may contribute to a more mechanistic understanding of spatial patterns in β‐diversity (e.g. Baselga 2010). Our simulations include two abundance‐based metrics, β_Bray‐Curtis_ and β_Ružička,_ that can be additively partitioned into independent nestedness and turnover components. We find the partitioning method described by Baselga (2013) generates turnover components that are independent of nestedness, whilst the method proposed by Podani, Ricotta & Schmera (2013) does not.

A principal component analysis indicated a large amount of redundancy among metrics. Yet our results highlight one property which is lacking among existing abundance‐based β‐diversity metrics. Three pieces of information are absent in samples of species assemblages; (i) how many species are missing in the sample, but present at the site, (ii) their abundances and (iii) whether they are shared or unshared between undersampled assemblage pairs. Abundance‐based β*‐*diversity metrics that estimate this information and adjust the value of β accordingly are one avenue for improving performance when there is undersampling. Recent developments in biodiversity sampling theory (Green & Plotkin 2007; Morlon *et al*. 2008; McGill 2011) and hierarchical Bayesian techniques that model the observation process (Kéry & Royle 2008) provide a useful starting point for developing such metrics.

The issues we have raised highlight that β‐diversity is a multi‐faceted concept. Any study measuring β‐diversity should be explicit about its goals (which properties should be emphasized) and assumptions (e.g. about sampling) when filtering the available metrics.

## Data accessibility

Code for simulations is available from figshare: http://dx.doi.org/10.6084/m9.figshare.1305115 (Barwell, Isaac & Kunin 2015).

## Supporting information


**Appendix S1.** β‐diversity metrics.
**Appendix S2.** Evaluation of metrics.
**Appendix S3.** Supplementary results.

## References

[jane12362-bib-0001] Anderson, M.J. , Crist, T.O. , Chase, J.M. , Vellend, M. , Inouye, B.D. , Freestone, A.L. *et al* (2011) Navigating the multiple meanings of beta diversity: a roadmap for the practicing ecologist. Ecology Letters, 14, 19–28. 21070562 10.1111/j.1461-0248.2010.01552.x

[jane12362-bib-0002] Baiser, B. , Olden, J.D. , Record, S. , Lockwood, J.L. & McKinney, M.L. (2012) Pattern and process of biotic homogenization in the New Pangaea. Proceedings of the Royal Society of London ‐ Series B: Biological Sciences, 279, 4772–4777. 23055062 10.1098/rspb.2012.1651 PMC3497087

[jane12362-bib-0003] Balata, D. , Piazzi, L. & Benedetti‐Cecchi, L. (2007) Sediment disturbance and loss of beta diversity on subtidal rocky reefs. Ecology, 88, 2455–2461. 18027747 10.1890/07-0053.1

[jane12362-bib-0004] Barton, P.S. , Cunningham, S.A. , Manning, A.D. , Gibb, H. , Lindenmayer, D.B. & Didham, R.K. (2013) The spatial scaling of beta diversity (ed A Baselga). Global Ecology and Biogeography, 22, 639–647.

[jane12362-bib-0005] Barwell, L.J. , Isaac, N.J.B. & Kunin, W.E. (2015) Measuring beta‐diversity with abundance data. Figshare. doi: 10.6084/m9.figshare.1305115 10.1111/1365-2656.12362 PMC4979660 25732937

[jane12362-bib-0006] Baselga, A. (2010) Partitioning the turnover and nestedness components of beta diversity. Global Ecology and Biogeography, 19, 134–143.

[jane12362-bib-0007] Baselga, A. (2013) Separating the two components of abundance‐based dissimilarity: balanced changes in abundance vs. abundance gradients (ed R Freckleton). Methods in Ecology and Evolution, 4, 552–557.

[jane12362-bib-0008] Beck, J. , Holloway, J.D. & Schwanghart, W. (2013) Undersampling and the measurement of beta diversity (ed D Orme). Methods in Ecology and Evolution, 4, 370–382.

[jane12362-bib-0009] Brown, J.H. (1984) On the relationship between abundance and distribution of species. American Naturalist, 124, 255–279.

[jane12362-bib-0010] Cassey, P. , Lockwood, J.L. , Olden, J.D. & Blackburn, T.M. (2008) The varying role of population abundance in structuring indices of biotic homogenization. Journal of Biogeography, 35, 884–892.

[jane12362-bib-0011] Chao, A. , Chazdon, R.L. , Colwell, R.K. & Shen, T.‐J. (2005) A new statistical approach for assessing similarity of species composition with incidence and abundance data. Ecology Letters, 8, 148–159.

[jane12362-bib-0012] Chao, A. , Chazdon, R.L. , Colwell, R.K. & Shen, T.‐J. (2006) Abundance‐based similarity indices and their estimation when there are unseen species in samples. Biometrics, 62, 361–371. 16918900 10.1111/j.1541-0420.2005.00489.x

[jane12362-bib-0013] Chase, J.M. (2003) Community assembly: when should history matter? Oecologia, 136, 489–498. 12836009 10.1007/s00442-003-1311-7

[jane12362-bib-0014] Chase, J.M. , Kraft, N.J.B. , Smith, K.G. , Vellend, M. & Inouye, B.D. (2011) Using null models to disentangle variation in community dissimilarity from variation in a‐diversity. Ecosphere, 2, Article 24.

[jane12362-bib-0015] Fisher, R.A. , Corbet, A.S. & Williams, C.B. (1943) The relation between the number of species and the number of individuals in a random sample of an animal population. Journal of Animal Ecology, 12, 42–58.

[jane12362-bib-0016] Gabriel, D. , Roschewitz, I. , Tscharntke, T. & Thies, C. (2006) Beta diversity at different spatial scales: plant communities in organic and conventional agriculture. Ecological Applications, 16, 2011–2021. 17069391 10.1890/1051-0761(2006)016[2011:bdadss]2.0.co;2

[jane12362-bib-0017] Green, J.L. & Plotkin, J.B. (2007) A statistical theory for sampling species abundances. Ecology Letters, 10, 1037–1045. 17803677 10.1111/j.1461-0248.2007.01101.x

[jane12362-bib-0018] Hankin, R.K.S. (2007) Introducing untb, an R Package For Simulating Ecological Drift Under the Unified Neutral Theory of Biodiversity. Journal of Statistical Software, 22, 1–15.

[jane12362-bib-0019] Harte, J. , McCarthy, S. , Taylor, K. , Kinzig, A. & Fischer, M.L. (1999) Estimating species‐area relationships from plot to landscape scale using species spatial‐turnover data. Oikos, 86, 45–54.

[jane12362-bib-0020] Holt, B.G. , Lessard, J.‐P. , Borregaard, M.K. , Fritz, S.A. , Araújo, M.B. , Dimitrov, D. *et al* (2013) An update of Wallace's zoogeographic regions of the world. Science, 339, 74–78. 23258408 10.1126/science.1228282

[jane12362-bib-0021] Jost, L. (2007) Partitioning diversity into independent alpha and beta components. Ecology, 88, 2427–2439. 18027744 10.1890/06-1736.1

[jane12362-bib-0022] Jost, L. , Chao, A. & Chazdon, R. (2011) Compositional similarity and β (beta) diversity Biological Diversity: Frontiers in Measurement and Assessment (eds Magurran A.E. & McGill B.J.), pp. 66–84. Oxford University Press, Oxford.

[jane12362-bib-0023] Keil, P. , Schweiger, O. , Kühn, I. , Kunin, W.E. , Kuussaari, M. , Settele, J. *et al* (2012) Patterns of beta diversity in Europe: the role of climate, land cover and distance across scales. Journal of Biogeography, 39, 1473–1486.

[jane12362-bib-0024] Kéry, M. & Royle, J.A. (2008) Hierarchical Bayes estimation of species richness and occupancy in spatially replicated surveys. Journal of Applied Ecology, 45, 589–598.

[jane12362-bib-0025] Koleff, P. , Gaston, K.J. & Lennon, J.J. (2003a) Measuring beta diversity for presence‐absence data. Journal of Animal Ecology, 72, 367–382.

[jane12362-bib-0026] Koleff, P. , Lennon, J.J. & Gaston, K.J. (2003b) Are there latitudinal gradients in species turnover? Global Ecology and Biogeography, 12, 483–498.

[jane12362-bib-0027] Legendre, P. (2014) Interpreting the replacement and richness difference components of beta diversity. Global Ecology and Biogeography, 23, 1324–1334.

[jane12362-bib-0028] Legendre, P. & De Cáceres, M. (2013) Beta diversity as the variance of community data: dissimilarity coefficients and partitioning. Ecology Letters, 16, 951–963. 23809147 10.1111/ele.12141

[jane12362-bib-0500] Lennon, J.J. , Koleff, P. , Greenwood, J.J.D. & Gaston, K.J. (2001) The geographical structure of British bird distributions: diversity, spatial turnover and scale. Journal of Animal Ecology, 70, 966–979.

[jane12362-bib-0029] McGill, B.J. (2010) Towards a unification of unified theories of biodiversity. Ecology Letters, 13, 627–642. 20337695 10.1111/j.1461-0248.2010.01449.x

[jane12362-bib-0030] McGill, B.J. (2011) Linking biodiversity patterns by autocorrelated random sampling. American Journal of Botany, 98, 481–502. 21613141 10.3732/ajb.1000509

[jane12362-bib-0031] McGlinn, D.J. & Hurlbert, A.H. (2012) Scale dependence in species turnover reflects variance in species occupancy. Ecology, 93, 294–302. 22624311 10.1890/11-0229.1

[jane12362-bib-0032] Morlon, H. , Chuyong, G. , Condit, R. , Hubbell, S. , Kenfack, D. , Thomas, D. *et al* (2008) A general framework for the distance‐decay of similarity in ecological communities. Ecology Letters, 11, 904–917. 18494792 10.1111/j.1461-0248.2008.01202.x PMC2613237

[jane12362-bib-0033] Oksanen, J. , Blanchet, F.G. , Kindt, R. , Legendre, P. , Minchin, P.R. , O'Hara, R.B. *et al* (2013) vegan: Community Ecology Package. R Package version 2.0‐10. http://cran.r-project.org/package=vegan

[jane12362-bib-0034] Podani, J. , Ricotta, C. & Schmera, D. (2013) A general framework for analyzing beta diversity, nestedness and related community‐level phenomena based on abundance data. Ecological Complexity, 15, 52–61.

[jane12362-bib-0035] Qian, H. & Ricklefs, R.E. (2007) A latitudinal gradient in large‐scale beta diversity for vascular plants in North America. Ecology Letters, 10, 737–744. 17594429 10.1111/j.1461-0248.2007.01066.x

[jane12362-bib-0036] R Development Core Team . (2014) R: A Language and Environment for Statistical Computing. Version 3.1.1. R Foundation for Statistical Computing, Vienna, Austria http://www.R-project.org/

[jane12362-bib-0037] Smith, A.B. (2010) Caution with curves: caveats for using the species–area relationship in conservation. Biological Conservation, 143, 555–564.

[jane12362-bib-0038] Whittaker, R.H. (1960) Vegetation of the Siskiyou Mountains, Oregon and California. Ecological Monographs, 30, 280–338.

[jane12362-bib-0039] Whittaker, R.H. (1972) Evolution and measurement of species diversity. Taxon, 21, 213–251.

[jane12362-bib-0040] Wiersma, Y.F. & Urban, D.L. (2005) Beta diversity and nature reserve system design in the Yukon, Canada. Conservation Biology, 19, 1262–1272.

