# Supplementary material for: Measuring β‐diversity with species abundance data
Source: J Anim Ecol. 2015 Mar 21;84(4):1112–22. doi: 10.1111/1365-2656.12362 (PMC4979660; doi:10.1111/1365-2656.12362)
Supplement: Supplementary file 1 — Appendix S1. β‐diversity metrics. Appendix S2. Evaluation of metrics. Appendix S3. Supplementary results. [file JANE-84-1112-s001.docx]

**Appendix S1** – The *β*-diversity metrics

Table S1 Formulae and references for 33 *β*-diversity metrics

|  | **Index** | **Formula** | **Minimum – Maximum** | **Data** | **Reference** |
| --- | --- | --- | --- | --- | --- |
| 1 | **sim** | $\beta=\frac{\min(b,c)}{\min\left( b, c \right)+ a}$ | 0 – 1 | Presence-absence | (Lennon *et al.* 2001) based on (Simpson 1949), as expressed by (Koleff *et al.* 2003) |
| 2 | **Classic Sørensen** | $\beta= 1-\frac{2a}{2a+b+c}$ | 0 – 1 | Presence-absence | (Sørensen 1948) as expressed by (Koleff *et al.* 2003) |
| 3 | **Classic Jaccard** | $\beta= 1-\frac{a}{a+b+c}$ | 0 – 1 | Presence-absence | (Jaccard 1912) as expressed by (Koleff *et al.* 2003) |
| 4 | **Chao Sørensen** | $\beta= 1-\frac{2\hat{U}\hat{V}}{\hat{U}+\hat{V}}$ | 0 - 1 | Abundance | (Chao *et al.* 2005) |
| 5 | **Chao Jaccard** | $\beta=1- \frac{\hat{U}\hat{V}}{\hat{U}+\hat{V}-\hat{U}\hat{V}}$ | 0 – 1 | Abundance | (Chao *et al.* 2005) |
| 6 | **Ružička** | $\beta=\frac{2\left( {\sum_{i=1}^{s} \left\vert x_{ij}-x_{ik} \right\vert}/{\sum_{i=1}^{s} \left\vert x_{ij}+x_{ik} \right\vert} \right)}{1+\left( {\sum_{i=1}^{s} \left\vert x_{ij}-x_{ik} \right\vert}/{\sum_{i=1}^{s} \left\vert x_{ij}+x_{ik} \right\vert} \right)}$ | 0 – 1 | Abundance | (Ružička 1958) |
| 7 | **Baselga Ružička turn** | $\beta=\frac{2(\min_{} B, C)}{A+\underset{}{2min} (B, C)}$ | 0 – 1 | Abundance | (Legendre 2014) |
| 8 | **Baselga Ružička nest** | $\beta=\left( \frac{\left\vert B-C \right\vert}{A+B+C} \right) \left( \frac{A}{A+2\min_{} (B, C)} \right)$ | 0 - 1 | Abundance | (Legendre 2014) |
| 9 | **Podani Ružička turn** | $\beta=\frac{2\underset{}{min(} B, C)}{A+B+C}$ | 0 – 1 | Abundance | (Podani *et al.* 2013) |
| 10 | **Podani Ružička nest** | $\beta=\left( \frac{\left\vert B-C \right\vert}{A+B+C} \right)$ | 0 – 1 | Abundance | (Podani *et al.* 2013) |
| 11 | **Bray-Curtis** | $\beta= \frac{\sum_{i=1}^{s} \left\vert x_{ij}-x_{ik} \right\vert}{\sum_{i=1}^{s} \left\vert x_{ij}+x_{ik} \right\vert}$ | 0 – 1 | Abundance | (Bray & Curtis 1957) |
| 12 | **Baselga B-C turn** | $\beta=\frac{\min_{} (B, C)}{A+\min_{} (B, C)}$ | 0 – 1 | Abundance | (Baselga 2013) |
| 13 | **Baselga B-C nest** | $\beta=\left( \frac{\left\vert B-C \right\vert}{2A+B+C} \right)\left( \frac{A}{A+\min_{} (B, C)} \right)$ | 0 – 1 | Abundance | (Baselga 2013) |
| 14 | **Podani B-C turn** | $\beta=\frac{2\underset{}{min(} B, C)}{2A+B+C}$ | 0 – 1 | Abundance | (Legendre 2014) |
| 15 | **Podani B-C nest** | $\beta=\left( \frac{\left\vert B-C \right\vert}{2A+B+C} \right)$ | 0 – 1 | Abundance | (Legendre 2014) |
| 16 | **Canberra** | $\beta=\frac{1}{S} \left[ \sum_{i=1}^{S} \left( \frac{\left\vert x_{ij}-x_{ik} \right\vert}{x_{ij}+x_{ik}} \right) \right]$ | 0 – 1 | Abundance | (Lance & Williams 1967) |
| 17 | **Morisita** | $\beta= 1- \frac{2 \sum x_{ij}x_{ik}}{\left( \lambda_{j}+ \lambda_{k} \right)N_{j}N_{k}}$ | 0 – 1 | Abundance | (Morisita 1959) |
| 18 | **Morisita-Horn** | $\beta=1- \frac{2 \sum x_{ij}x_{ik}}{\left[ \left( \sum x_{ij}^{2}/N_{j}^{2} \right)+ \left( \sum x_{ik}^{2}/N_{ik}^{2} \right) \right]N_{j}N_{k}}$ | 0 – 1 | Abundance | (Horn 1966) |
| 19 | **Horn** | $\beta= 1- \frac{\sum\left[ \left( x_{ij}+x_{ik} \right)\log(x_{ij}x_{ik}) \right]-\sum\left( x_{ij}\log x_{ij} \right)-\sum\left( x_{ik}\log x_{ik} \right)}{\left[ \left( N_{j}+N_{k} \right)\log\left( N_{j}N_{k} \right) \right]-\left( N_{j}\log N_{j} \right)-\left( N_{k}\log N_{k} \right)}$ | 0 – 1 | Abundance | (Horn 1966) |
| 20 | **Kulczynski** | $\beta=1-0.5\left( \frac{\sum\min{(x}_{ij},x_{ik})}{N_{j}}+ \frac{\sum\min{(x}_{ij},x_{ik})}{N_{k}} \right)$ | 0 – 1 | Abundance | (Kulczynski 1927) |
| 21 | **Renkonen** | $\beta= 1-\sum_{i=1} \min\left( p_{ij},p_{ik} \right)$ | 0 – 1 | Abundance | (Renkonen 1938) |
| 22 | **NESS_(m = 50)_** | $\beta=1-\frac{2\sum_{i=1}^{S} \mu_{ij}(m)\mu_{ik}(m)}{\sum_{i=1}^{S} {[\mu_{ij}\left( m \right)]}^{2} +\sum_{i=1}^{S} {[\mu_{ik}\left( m \right)]}^{2}}$ | 0 – 1 | Abundance | (Grassle & Smith 1976) |
| 23 | **Gower** | $\beta= \frac{1}{S}\sum_{i=1}^{S} \frac{\left\vert x_{ij}-x_{ik} \right\vert}{\max( x_{i})-\min(x_{i})}$ | 0 – 1 | Abundance | (Gower 1971) |
| 24 | **Jost Shannon** | $\beta=\frac{{}^{1}{D_{\gamma}}}{{}^{1}{D_{\bar{\alpha}}}}-1$ | 0 – 1 | Abundance | (Jost 2006, 2007), rescaled from 0 - 1 |
| 25 | **Jost Simpson** | $\beta=\frac{{{}^{2}D}_{\gamma}}{{{}^{2}D}_{\alpha}}-1$ | 0 – 1 | Abundance | (Jost 2006, 2007), rescaled from 0 – 1 |
| 26 | **Euclidean distance** | $\beta=\sqrt{\sum_{i=1}^{S} \left( x_{ij}-x_{ik} \right)^{2}}$ | 0 – 1 | Abundance | (Clifford & Stephenson 1975) |
| 27 | **Average Euclidean distance** | $\beta=\sqrt{\frac{\sum_{i=1}^{S} \left( x_{ij}-x_{ik} \right)^{2}}{S}}$ | 0 – no upper limit | Abundance | see (Krebs 1998) |
| 28 | **Manhattan** | $\beta= \sum_{i=1}^{S} \left\vert x_{ij}- x_{ik} \right\vert$ | 0 – no upper limit | Abundance | see (Krebs 1998) |
| 29 | **Alternate Gower** | $\beta= \frac{\sum_{i=1}^{S} \left\vert x_{ij}- x_{ik} \right\vert}{\sum_{i=1}^{S} w_{i}}$ | 0 – no upper limit | Abundance | (Anderson *et al.* 2006) |
| 30 | **CYd** | $\beta= \frac{1}{S}\sum_{i=1}^{S} \frac{n_{i}\log\frac{1}{2}-x_{ij}\log x_{ik}+ x_{ik}\log x_{ij}}{n_{i}}$ | 0 – no upper limit | Abundance | (Cao *et al.* 1997) |
| 31 | **Binomial** | $\beta= \frac{\sum_{i=1}^{S} x_{ij}{\log{(x}_{ij}}/{n_{i})} +{x_{ik}\log{(x}_{ik}}/{n_{i}) -n_{i}\log\frac{1}{2}}}{n_{i}}$ | 0 – no upper limit | Abundance | (Anderson & Millar 2004) |
| 32 | **Lande Shannon** | $\beta=H_{\gamma}- \overline{H}_{\alpha}$ | 0 - no upper limit | Abundance | (Lande 1996) |
| 33 | **Lande Simpson** | $\beta=\lambda_{\gamma}- \overline{\lambda}_{\alpha}$ | 0 - no upper limit | Abundance | (Lande 1996) |

**Table S2 Notation used in formulae for the 33 *β*-diversity metrics.**

| Symbol | Definition |
| --- | --- |
| *β* | Beta-diversity |
| *a* | Matching component: The number of species shared between the focal and contrasted assemblages |
| *b* | Matching component: The number of species unique to the contrasted assemblage and absent from the focal assemblage |
| *c* | Matching component: The number of species unique to the focal assemblage and absent from the contrasted assemblage |
| *x_ij_* | The number of individuals of species *i* in assemblage *j* |
| *x_ik_* | The number of individuals of species *i* in assemblage *k* |
| *x_i_* | ${=x}_{ij}+x_{ik}$ = total number of individuals of species *i* in assemblages *j* and *k* combined |
| *S* | The total number of species in focal and contrasted assemblages |
| *N_j_* | Total number of individuals in assemblage *j* |
| *N_k_* | Total number of individuals in assemblage *k* |
| *p_ij_* | Relative abundance of species *i* in assemblage *j* |
| **Table S2 continued** | |
| Symbol | Definition |
| *p_ik_* | Relative abundance of species *i* in assemblage *k* |
| $H_{\bar{\alpha}}$ | $\frac{1}{2}\left( \sum_{i=1}^{S} p_{ij}\log p_{ij} +\sum_{i=1}^{S} p_{ik}\log p_{ik} \right)$ = mean of Shannon entropy (Shannon 1948) in assemblages *j* and *k* |
| $H_{\gamma}$ | $\sum_{i=1}^{S} p_{ij}+p_{ik}\log p_{ij}+p_{ik}$ = Shannon entropy (Shannon 1948) for assemblages *j* and *k* pooled |
| $\lambda_{\bar{\alpha}}$ | $=\frac{1}{2}\left[ \frac{\sum[x_{ij} \left( x_{ij}- 1 \right)]}{N_{j}\left( N_{j}- 1 \right)} \right]+\left[ \frac{\sum[x_{ik} \left( x_{ik}- 1 \right)]}{N_{k}\left( N_{k}- 1 \right)} \right]$ = mean of Simpson’s index of diversity (Simpson 1949) for assemblages *j* and *k* |
| $\lambda_{\gamma}$ | $=\left[ \frac{\sum[x_{i} \left( x_{i}- 1 \right)]}{x_{i}\left( x_{i}- 1 \right)} \right]$ = Simpson’s index of diversity (Simpson 1949) for assemblages *j* and *k* pooled |
| *µ_ij_ (m)* | $=1-{(1-p_{ij})}^{m}$, where *m* is the size of a random sample drawn from a population. |
| *µ_ik_ (m)* | $=1-{(1-p_{ik})}^{m}$, where *m* is the size of a random sample drawn from a population |
| *w_i_* | a weight applied to species *i* in order to exclude joint absences. If *x_ij_ + x_ik_* = 0, then *w_i_* = 0, if *x_ij_ + x_ik_* > 0, then *w_i_* = 1 |
| ${}^{1}{D_{\alpha}}$ | $=$ $\exp\left[ -w_{1}\sum_{i=1}^{S} p_{i1}\log p_{i1}-w_{2}\sum_{i=1}^{S} p_{i2}\log p_{i2} \right]$ |
| ${}^{1}{D_{\gamma}}$ | $= exp\left[ -\sum_{i=1}^{S} {w_{j}p}_{ij}-\sum_{i=1}^{S} {w_{k}p}_{ik} \right]$, where *w*_j_ and *w*_k_ are weights reflecting the relative sizes of assemblages *j* and *k* |
| ${}^{2}{D_{\alpha}}$ | $=\left( \frac{1}{N}\sum_{i=1}^{S} p_{i1}^{2}+\frac{1}{N}\sum_{i=1}^{S} p_{i2}^{2} \right)^{\frac{1}{1-2}}$ |
| ${}^{2}{D_{\gamma}}$ | =$\left\{ \sum_{i=1}^{S} \left[ \frac{1}{N}\left( p_{i1}+p_{i2} \right)^{\frac{1}{1-2}} \right]^{2} \right\}^{\frac{1}{1-2}}$ |
| *D_jk_* | Shared species, present in assemblage *j* and assemblage *k* |
| *I* [expr] | Indicator function, *I =* 1 if expression is true, *I* = 0 if expression is false |
| **Table S2 continued** | |
| Symbol | Definition |
|  |  |
| $f_{1+}$ | $=\sum_{i=1}^{D_{jk}} I\left[ x_{ij}=1,x_{ik}\geq1 \right]$ = observed number of shared species that are singletons in assemblage *j* |
| $f_{+1}$ | $=\sum_{i=1}^{D_{jk}} I\left[ x_{ik}=1,x_{ij}\geq1 \right]$ = observed number of shared species that are singletons in assemblage *k* |
| $f_{2+}$ | $=\sum_{i=1}^{D_{jk}} I\left[ x_{ij}=2,x_{ik}\geq1 \right]$ = observed number of shared species that are doubletons in assemblage *j* |
| $f_{+2}$ | $=\sum_{i=1}^{D_{jk}} I\left[ x_{ik}=2,x_{ij}\geq1 \right]$ = observed number of shared species that are doubletons in assemblage *k* |
| $\hat{U}$ | $=\sum_{i=1}^{D_{jk}} \frac{x_{ij}}{N_{j}}+ \frac{N_{k}-1}{N_{k}}\frac{f_{+1}}{2f_{+2}}\sum_{i=1}^{D_{jk}} \frac{x_{ij}}{N_{j}}I\left( x_{ik}=1 \right)$ |
| $\hat{V}$ | $=\sum_{i=1}^{D_{jk}} \frac{x_{ik}}{N_{k}}+ \frac{N_{j}-1}{N_{j}}\frac{f_{1+}}{2f_{2+}}\sum_{i=1}^{D_{jk}} \frac{x_{ik}}{N_{k}}I\left( x_{ij}=1 \right)$ |

**Appendix S2 –** Evaluation of metrics

**Table S3** Summary of 16 conceptual (C1-16) and two sampling properties (S1-S2 ) of abundance-based *β-*diversity metrics. Properties and their evaluation are described in the methods section. The scores were standardised by the range of observed values for metric *i*, $\beta_{i, range}$ in each test.

| **Property** | **Score for metric *i*** |
| --- | --- |
| C1. Independent of *α*-diversity | ${C1}_{i}=\sqrt{\frac{1}{n_{t}n_{\alpha}}\sum_{t=1}^{n_{t}} \sum_{\alpha=1}^{n_{\alpha}} \left( \frac{\beta_{i,t,\alpha,}- \beta_{i,t,\alpha max}}{\beta_{i, range}} \right)^{2}}$, where $\beta_{i, t,\alpha}$ and $\beta_{i,t,\alpha max}$ are median *β*-diversity for metric *i* at turnover *t* and *α*-diversity *α* and *α_max_*, respectively, $\beta_{i, range}$ is the range of observed *β*-diversity for metric *i* in this test, *n_t_*  is the number of turnover levels, here 6,  *n_α_*  is the number of levels of  *α-*diversity, here 10. |
| C2. *β* is cumulative along a gradient of species turnover | ${C2}_{i}= \sqrt{\frac{1}{n_{t}n_{g}n_{j}}\sum_{t=1}^{n_{t}} \sum_{g=1}^{n_{g}} \sum_{j=1}^{n_{j}} \left( \frac{\left( \beta_{i,t,g,j}^{A, C}-( \beta_{i, t,g,j}^{A, B}+\beta_{i,t,g,j}^{B,C}) \right)}{\beta_{i, range}} \right)^{2}}$, where $\beta_{i, t,g, j}^{A, C}$, is *β*-diversity for metric *i* between assemblages A and C at turnover *t*, under a gradient of strength *g* in simulation *j*, $\beta_{i,t,g, j}^{A,B}+ \beta_{i,t,g, j}^{B,C}$is *β*-diversity expected under additivity, $\beta_{i, range}$ is the range of *β*-diversity for metric *i* in this test, *n_t_* is the number of turnover levels, here 6,  *n_g_*  is the number of gradient strengths, here 7, and *n*_j_ is the number of simulations at each unique combination of *t* and *g*, here 10000. |
| C3. Similarity is probabilistic when assemblages are independently and identically distributed | ${C3}_{i}=\sqrt{\frac{1}{n_{t}n_{j}}\sum_{t=1}^{n_{t}} \sum_{j=1}^{n_{j}} \left( \frac{\left. \left( {1-\beta}_{i,t, j}^{A,C} \right)- \left( {1-\beta}_{i,t, j}^{A,B} \right)\left( 1-\beta_{i,t, j}^{B,C} \right) \right.}{\beta_{i, range}} \right)^{2}}$, where $\beta_{i,t,j}^{A,C}$ is the value of *β*-diversity for metric *i* between assemblages A and C at turnover *t* in simulation *j* and $\beta_{i,j,t}^{A,B}\beta_{i,j,t}^{B,C}$ is *β*-diversity expected under probabilistic similarity, $\beta_{i, range}$ is the range of observed *β*-diversity for metric *i* in this test, *n_t_* is the number of turnover levels and *n_j_* is the number of simulations at each level of *t*, here 10000. |
| C4. Minimum of zero and positive | C4_i_ = TRUE / FALSE for $\beta_{i}^{A, A}=0$and $\beta_{i}^{A, B}\geq0$ where $\beta_{i}^{A, A}$ and $\beta_{i}^{A, B}$ are median *β*-diversity for metric *i* in 10000 simulations for two identical assemblages and two different assemblages where there is either species turnover, decoupling of species ranks or evenness differences. |
| C5. Monotonic: *β* increases in a series of assemblages with increasing species turnover | C5*_i_* = TRUE / FALSE for if *t_A,B_ < t_A,C_*  then $\beta_{i,t}^{A,B}< \beta_{i,t}^{A,C}$ , where  *t_A,B_* and *t_A,C_* are the proportion of species turned over between assemblages *A* and *B* and between *A* and *C,* respectively and $\beta_{i, t}^{A,B}$ and $\beta_{i,t}^{A,C}$ are median *β-*diversity in 10000 simulations for metric *i* at increasing levels of turnover, *t*. |
| C6. Monotonic:  *β* increases in a series of assemblages with increasing decoupling of species ranks | C6*_i_ =* TRUE / FALSE for if *r_A,B_ < r_A,C_*  then $\beta_{i, r}^{A,B}> \beta_{i, r}^{A,C}$, where *r*_A,B_ and  *r_A,C_* are the partial correlation between species ranks in assemblages *A* and B and *A* and *C*, respectively and $\beta_{i, r}^{A,B}$and $, \beta_{i,r}^{A,C}$ are median *β-*diversity in 10000 simulations for metric *i* with incrementally decreasing correlation between species ranks, *r*. |
| C7. Monotonic:  *β* increases in a series of assemblages with increasing evenness differences | C7*_i_ =* TRUE / FALSE for if *ΔE_A,B_ < ΔE_A, C_*  then $\beta_{i}^{A, B}< \beta_{i}^{A, C}$, where *ΔE*_A, B_ and  *ΔE_A, C_*  are the difference in evenness between assemblages *A* and *B* and *A* and *C*, respectively and $\beta_{i}^{A,B}$ and $\beta_{i}^{A,C}$ are median *β-*diversity in 10000 simulations for metric *i* with incrementally increasing levels of evenness differences, *ΔE*. |
| C8. *β* when extreme decoupling of species ranks < *β* when species turnover is complete | C8*_i_ =* TRUE / FALSE for *β_i, tmax, rmax_* >  *β_i, tmin, rmin_*_,_ where  *β_i, tmax, rmax_* and  *β_tmin, rmin_* are the median values of *β*-diversity for metric *i* under complete species turnover and no decoupling of species and under extreme decoupling of species ranks and no species turnover, respectively. |
| C9. *β* under extreme differences in evenness < *β*  when species turnover is complete | C9_i_ = TRUE / FALSE for *β_i,tmax, ΔEmin_* >  *β_i_,_tmin, ΔEmax_*, where  *β_i,tmax, ΔEmin_* and  *β_i, tmin, ΔEmax_* are the median values of *β*-diversity under complete species turnover and no evenness difference and under no species turnover and extreme evenness difference, respectively. |
|  |  |
| C10. Fixed upper bound | C10_i_ = TRUE/FALSE for $\frac{1}{2}\sum\beta_{i, t_{max}}^{2}$ = 1, where $\beta_{i}$ is the value of *β-*diversity for metric *i* when there is complete species turnover between assemblages. |
| C11. Symmetry | C11_i_ = TRUE/FALSE for *β_i,_ _A, B_* =  *β_i,B, A_*, where  *β_i_,_A, B_* and  *β_i,B, A_* are the values of *β*-diversity for metric *i* for assemblages A and B and B and A, respectively |
| C12. Double-zero asymmetry | C12_i_ = TRUE / FALSE for *ab* and *pr* in 1:10,  *β_i,_* _ab_ =  *β_i,, ab=0_* and  *β_i,_* _pr_ <  *β_i,, pr=0_* where  *β_i,_* _a_ and  *β_i,, a=0_* are the values of  *β*-diversity for metric *i* when *ab* double absences and no double absences, respectively, have been added to the assemblage pair.  *β_i,_* _pr_ and  *β_i,, pr=0_* are the values of  *β*-diversity for metric *i* when *pr* double presences and no double presences, respectively, have been added to the assemblage pair. |
| C13. *β* does not decrease in a series of nested assemblages | C13_i_ = TRUE / FALSE for *S_1,2_ < S_1,3_*  then $\beta_{i,t}^{1,2}\leq\beta_{i,t}^{1,3}$ , where *S* is difference in species richness between assemblages *1* and *2* and *1* and *3,* respectively and $\beta_{i}^{1,2}$ and $\beta_{i}^{1,3}$ are median *β-*diversity of assemblages *1* and *2* and *1* and *3,* respectively, for metric *i* at turnover, *t* in 10000 simulations. |
| C14. Independent of species replication | ${C14}_{i} =\sqrt{\frac{1}{n_{t}n_{x}}\sum_{t=1}^{n_{t}} \sum_{x=1}^{n_{x}} \left( \frac{\beta_{i,t,x}- \beta_{i,t,}}{\beta_{i,range}} \right)^{2}}$, where $\beta_{i,t}$ is median *β*-diversity in 10000 simulations for metric *i* at turnover *t* and $\beta_{i,t, x}$ is median *β*-diversity when *x* identical subsets are pooled (species replication), $\beta_{i, range}$ is the range of observed *β*-diversity for metric *i* in this test, *n_t_*  = the number of turnover levels, here 6, and *n_x_* is the number of levels of species replication, *x* , here 10*.* |
| C15. Independent of measurement units | ${C15}_{i} =\sqrt{\frac{1}{n_{t}n_{cc}}\sum_{t=1}^{n_{t}} \sum_{cc=1}^{n_{cc}} \left( \frac{\beta_{i,t,cc}- \beta_{i,t}}{\beta_{i,range}} \right)^{2}}$, where $\beta_{i,t}$ is median *β*-diversity in 10000 simulations for metric *i* at turnover *t* and $\beta_{i,t,cc}$ is median *β*-diversity when abundances in both assemblages are multiplied by a constant factor *cc*. $\beta_{i, range}$ is the range of observed *β*-diversity for metric *i* in this test, *n_t_*  is the number of turnover levels, here 6 and *n_c_* is the number of levels of the constant factor, *cc* , here 10*.* |
| C16. Independent of differences in abundance | ${C16}_{i} =\sqrt{\frac{1}{n_{t}n_{c}}\sum_{t=1}^{n_{t}} \sum_{c=1}^{n_{c}} \left( \frac{\beta_{i,t,c}- \beta_{i,t}}{\beta_{i,range}} \right)^{2}}$, where $\beta_{i,t}$ is median *β*-diversity in 10000 simulations for metric *i* at turnover *t* and $\beta_{i,t,c}$ is median *β*-diversity when abundances in one assemblage are multiplied by a constant factor *c*. $\beta_{i, range}$ is the range of observed *β*-diversity for metric *i* in this test, *n_t_*  is the number of turnover levels, here 6 and *n_c_* is the number of levels of the constant factor, *c* , here 10*.* |
| S1. Independent of sample size | ${S1}_{i} =\sqrt{\frac{1}{n_{t}n_{N}}\sum_{t=1}^{n_{t}} \sum_{N=1}^{n_{N}} \left( \frac{\beta_{i,t,N}- \beta_{i,t,Nmax}}{\beta_{i,range}} \right)^{2}}$, where $\beta_{i,t,N}$ and $\beta_{i,t,Nmax}$ are median *β*-diversity in 10000 simulations for metric *i* at turnover *t* and sample sizes *N* and *N*_max ,_ respectively, $\beta_{i, range}$ is the range of observed *β*-diversity for metric *i* in this test, *n_t_*  = the number of turnover levels, here 6 and *n_N_* is the number of sample size levels, here 16*.* |
| S2. Independent of unequal sample sizes | ${S2}_{i}=\sqrt{\frac{1}{n_{t}n_{\Delta N}}\sum_{t=1}^{n_{t}} \sum_{\Delta N=1}^{n_{\Delta N}} \left( \frac{\beta_{i, t,\Delta N}- \beta_{i, t,\Delta Nmin}}{\beta_{i, range}} \right)^{2}}$, where $\beta_{i,t,\Delta N}$ and $\beta_{i. t,\Delta Nmin}$are median *β*-diversity in 10000 simulations for metric *i* at turnover *t* and sample size difference $\Delta N$ and ${\Delta N}_{min}$, respectively, $\beta_{i, range}$ is the range of observed *β*-diversity for metric *i* in this test, *n_t_*  = the number of turnover levels, here 6 and *n_ΔN_* is the number of levels of sample size difference, here 16. |
|  |  |
|  |  |
|  |  |

**Table S4** Summary of five personality properties (P1-5). Score is the method used to evaluate *β-*diversity metric *i* for each property.

| **Personality** | **Score** |
| --- | --- |
| P1. Sensitivity to differences in *α*-diversity | ${P1}_{i}=\sqrt{\frac{1}{n_{\Delta\alpha}n_{t}}\sum_{t=1}^{n_{t}} \sum_{\Delta\alpha=1}^{n_{\Delta\alpha}} \left( \frac{\beta_{i,t,\Delta\alpha,}- \beta_{i,t,\Delta\alpha min,}}{\beta_{i,range}} \right)^{2}}$, where $\beta_{i,t,\Delta\alpha}$ and $\beta_{i,t,\Delta\alpha min}$ are the median values of *β*-diversity for metric *i* at turnover *t* and sample size difference $\Delta\alpha$ and ${\Delta\alpha}_{min}$, respectively, $\beta_{i, range}$ is the range of  *β*-diversity for metric *i* in this simulation and *n* is the number of unique combinations of *Δα* and *t*. |
| P2. Relative sensitivity to nestedness and turnover components of *β* | ${P2}_{i}=\frac{\beta_{i,tmax, smin}}{\beta_{i,tmin, smax}}$, where $\beta_{i, tmax, smin}$ and $\beta_{i,tmin, smax}$ are the median values of *β*-diversity under complete species turnover and no species nestedness and for extreme species loss and no species turnover, respectively. |
| P3. Relative sensitivity to decoupling of species ranks and species turnover components of *β* | ${P3}_{i}=\frac{\beta_{i, tmax, rmax}}{\beta_{i, tmin, rmin}}$, where $\beta_{i, tmax, rmax}$ and $\beta_{i,tmin, rmin}$ are the median values of  *β*-diversity for metric *i* under complete species turnover and no decoupling of species ranks and under extreme decoupling of species ranks and no species turnover, respectively. |
| P4. Relative sensitivity to evenness differences and species turnover components of *β* | ${P4}_{i}=\frac{\beta_{i, tmax, \Delta Emin}}{\beta_{i, tmin,\Delta E=max}}$, where $\beta_{i,tmax, \Delta Emin}$ and $\beta_{i, tmin, \Delta Emax}$are the median values of  *β*-diversity for metric *i* under complete species turnover and no evenness differences and under extreme evenness difference and no species turnover, respectively |
| P5. Relative sensitivity to turnover in rare versus common species | ${P5}_{i}=\frac{\beta_{i, n_{min}}}{\beta_{i, n_{max}}}$, where $\beta_{i, n_{min}}$ and $\beta_{i, n_{max}}$are the values of$\beta$ for metric *i* when the rarest and commonest species, respectively, are turned over. |

**Appendix S3** – **Supplementary Results**

**
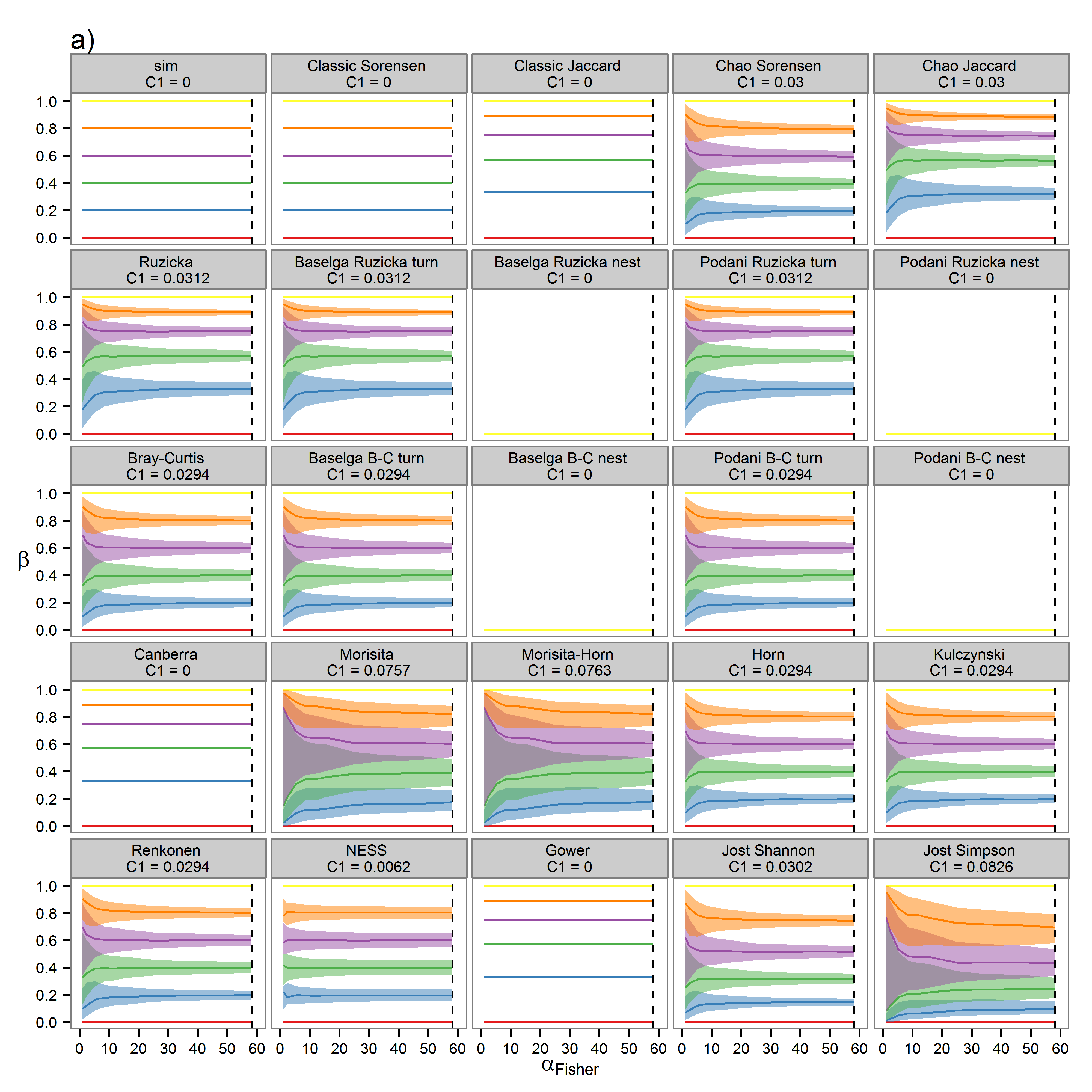
** Figures S1-S13 are the results of simulations testing the 33 metrics in Table S1 against the 16 conceptual and two sampling and in Table S3 and five personality traits in Table S4. On each of the *x* axes is a test-specific parameter describing some aspect of assemblage structure. The scores for each metric are shown above the plots and were calculated using the methods in Tables S3 and S4.

Fig. S1 Effect of *α*-diversity of assemblages (e.g. both low *α* or both high *α*) on the value of *β* for a) 25 metrics with a fixed upper limit and b) 8 metrics with no maxima. Solid lines and shaded areas are the median and interquartile range, respectively, of *β* for 10000 simulations at each unique combination of species turnover, *t*, and Fisher’s *α*-diversity, *α_Fisher_*. Metrics scored for desirable property C1, independence of *α*-diversity. Vertical dashed black lines intersect the reference values of median *β* at high *α_Fisher_*.

**
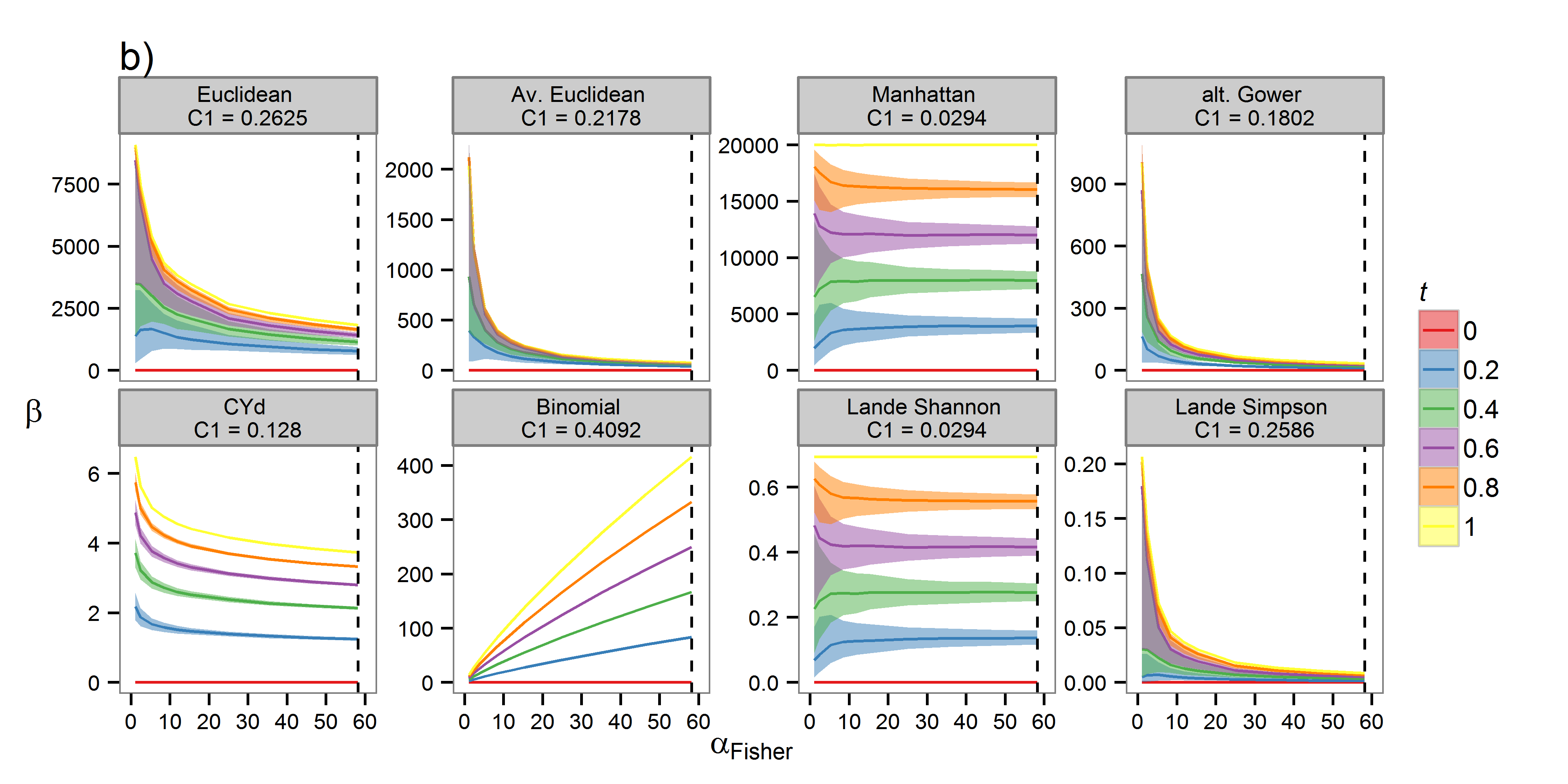
**

**
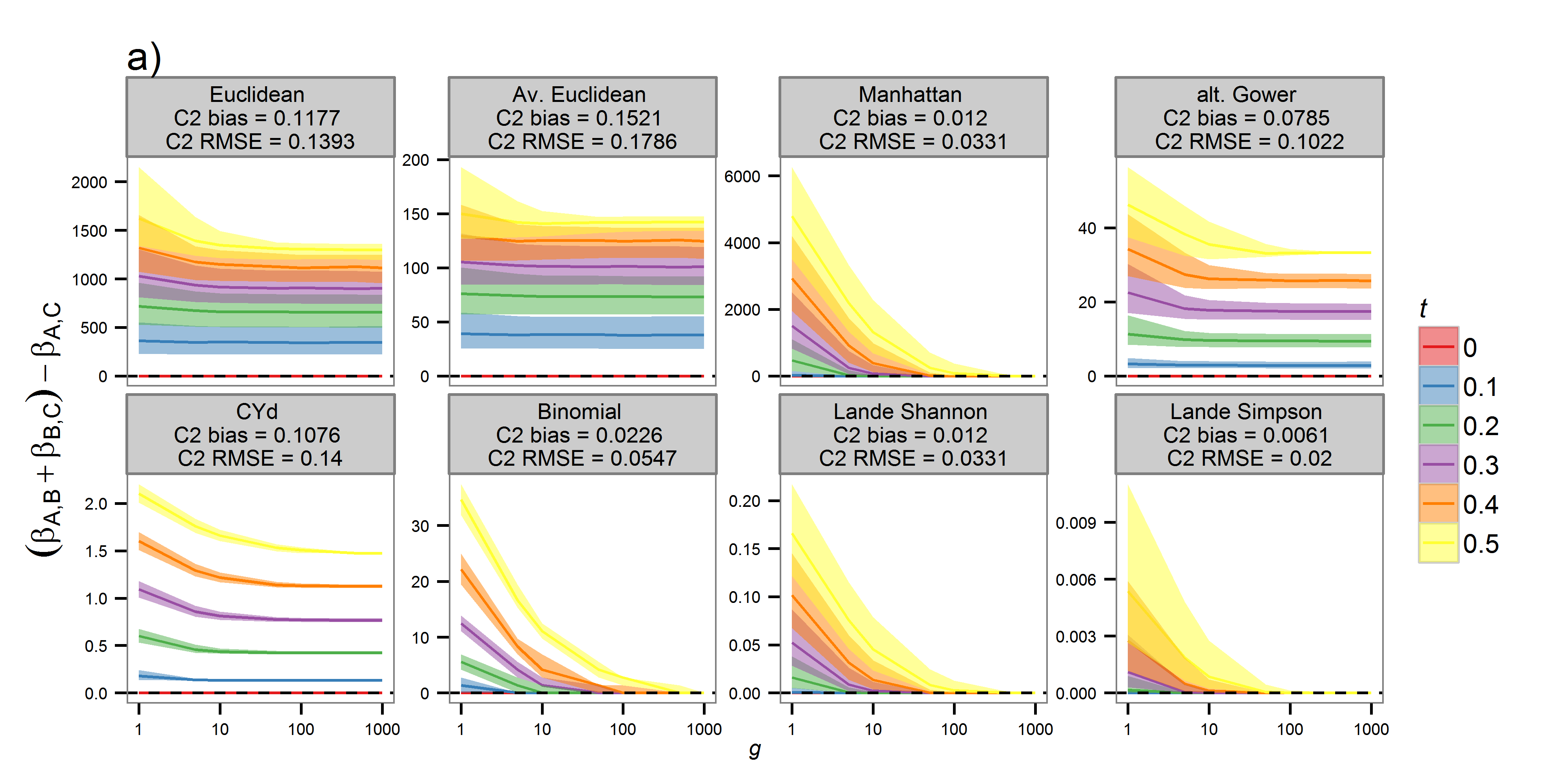

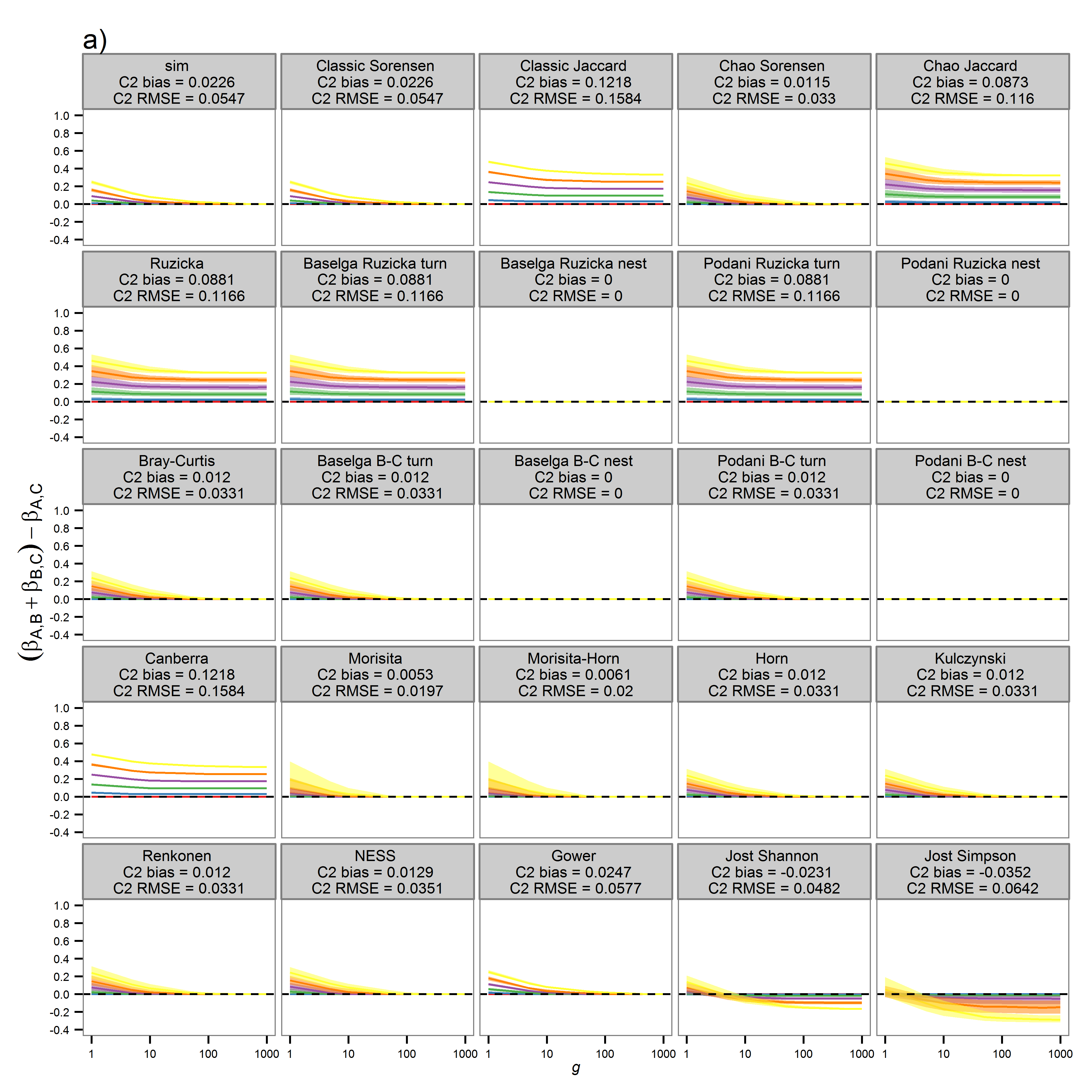
**

Fig. S2 Cumulative *β*-diversity under different strengths directional species turnover along a simulated environmental gradient, *g* for a) 25 metrics with a defined upper limit b) 8 metrics with no maxima. Solid lines and shaded areas are the median and interquartile range, respectively, of the differences between observed *β_A,C_* and that predicted if *β* were cumulative along a gradient of turnover (*β_A,B+_β_B,C_*), based on 10000 simulations at each unique combination of species turnover, *t*, and environmental gradient, *g*. Metrics are scored for property C2, *β* is cumulative along a gradient of species turnover. Horizontal dashed black lines at 0 represent perfect cumulative behaviour. Scores for bias are also presented in order to evaluate whether the metrics are systematically sub- or supra-additive.


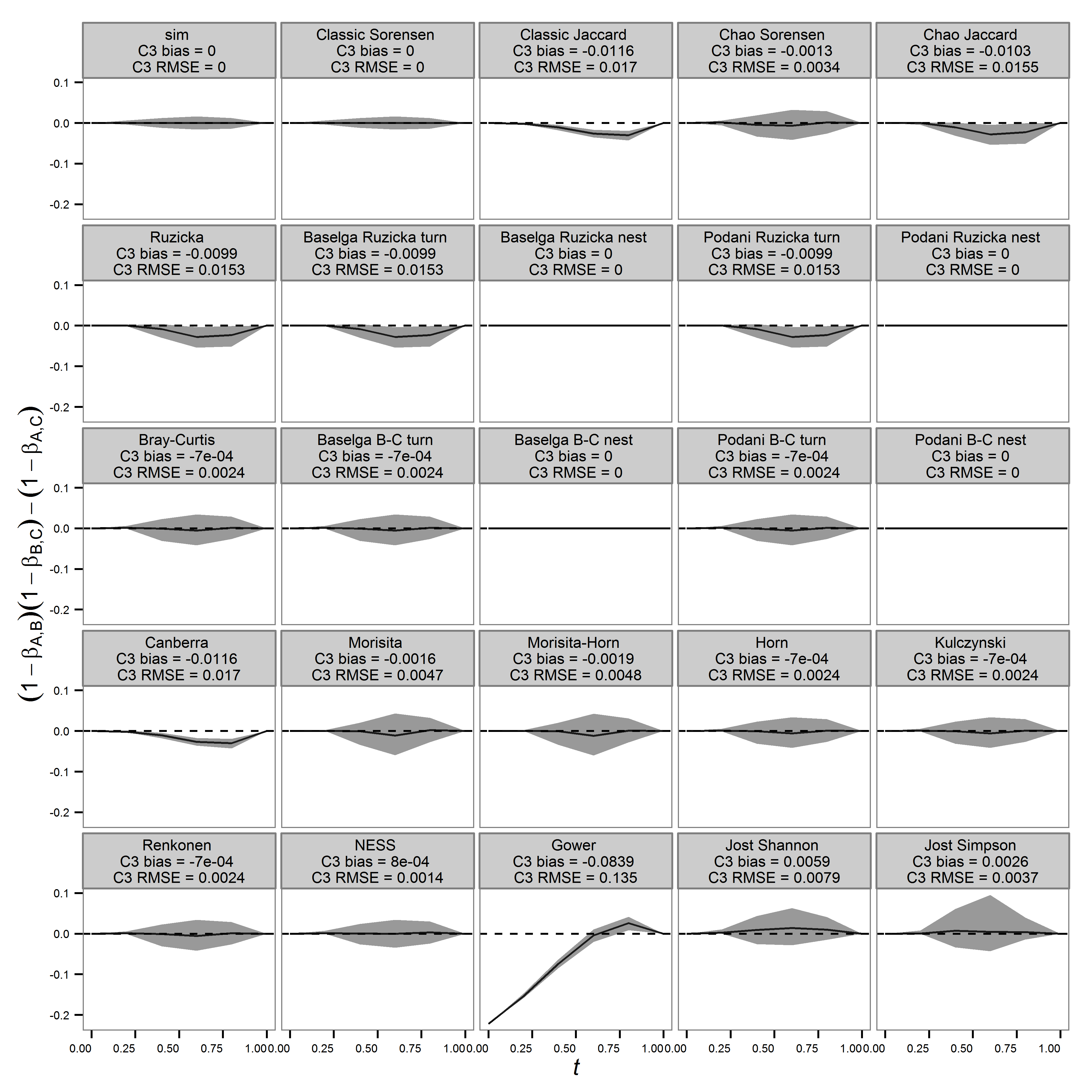


Fig. S3 Probabilistic *β*-diversity when assemblages are independent draws form a well-mixed metacommunity for 25 metrics with fixed upper limits. Metrics without upper limits do not have a similarity complement. Solid lines and shaded areas are the median and interquartile range, respectively, of the differences between observed similarity*,* (1-*β_A,C_*) and that predicted if similarity were probabilistic, (1-*β_A,B_*)(1-*β_B,C_*) based on 10000 simulations at each level of species turnover, *t*. Metrics are scored for desirable property C3, similarity is probabilistic when assemblages are independently and identically distributed. Horizontal dashed black lines at 0 represent perfect probabilistic behaviour. The scores for bias are also presented to evaluate whether the metrics are systematically sub- or supra-probabilistic.


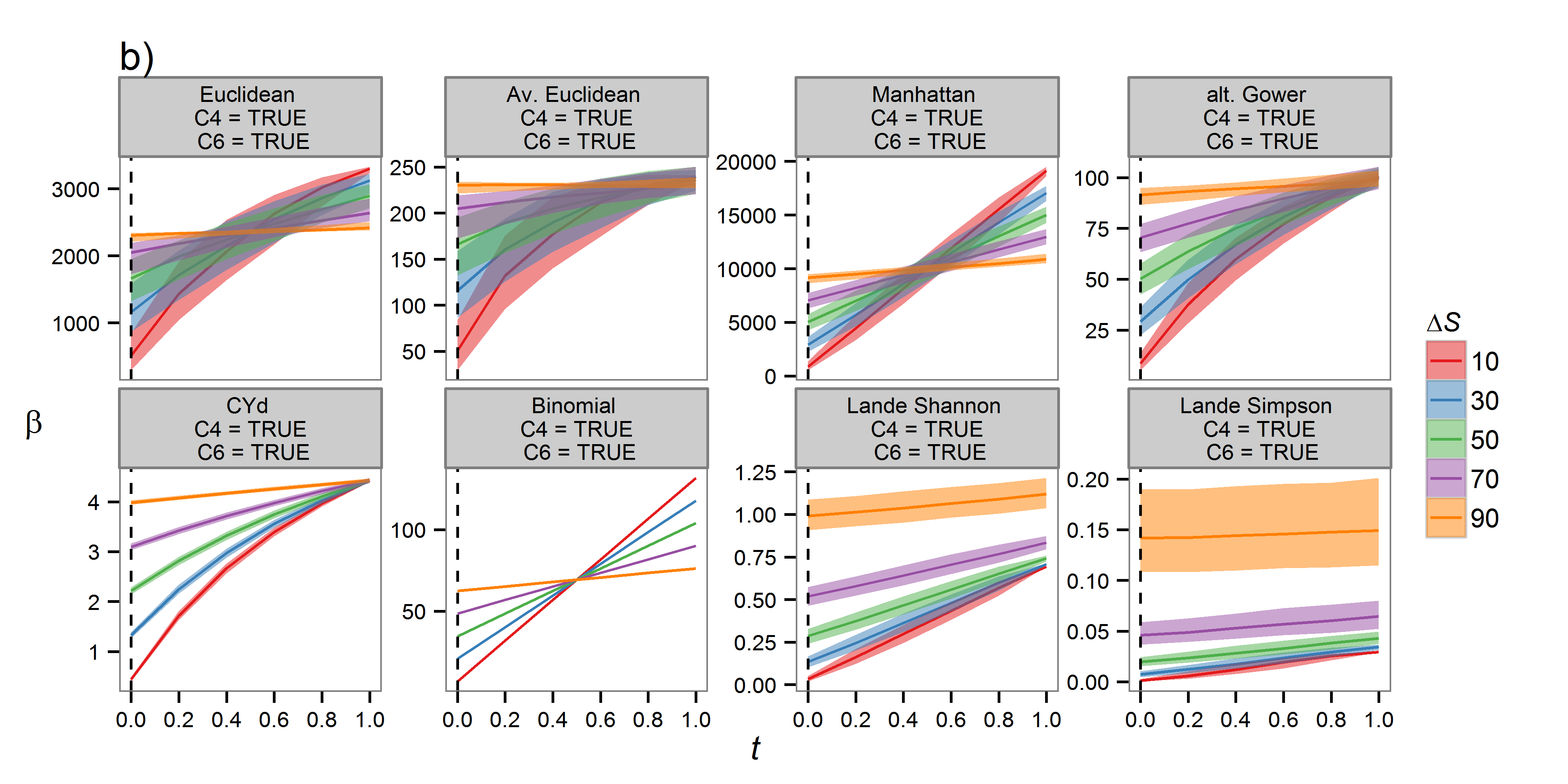

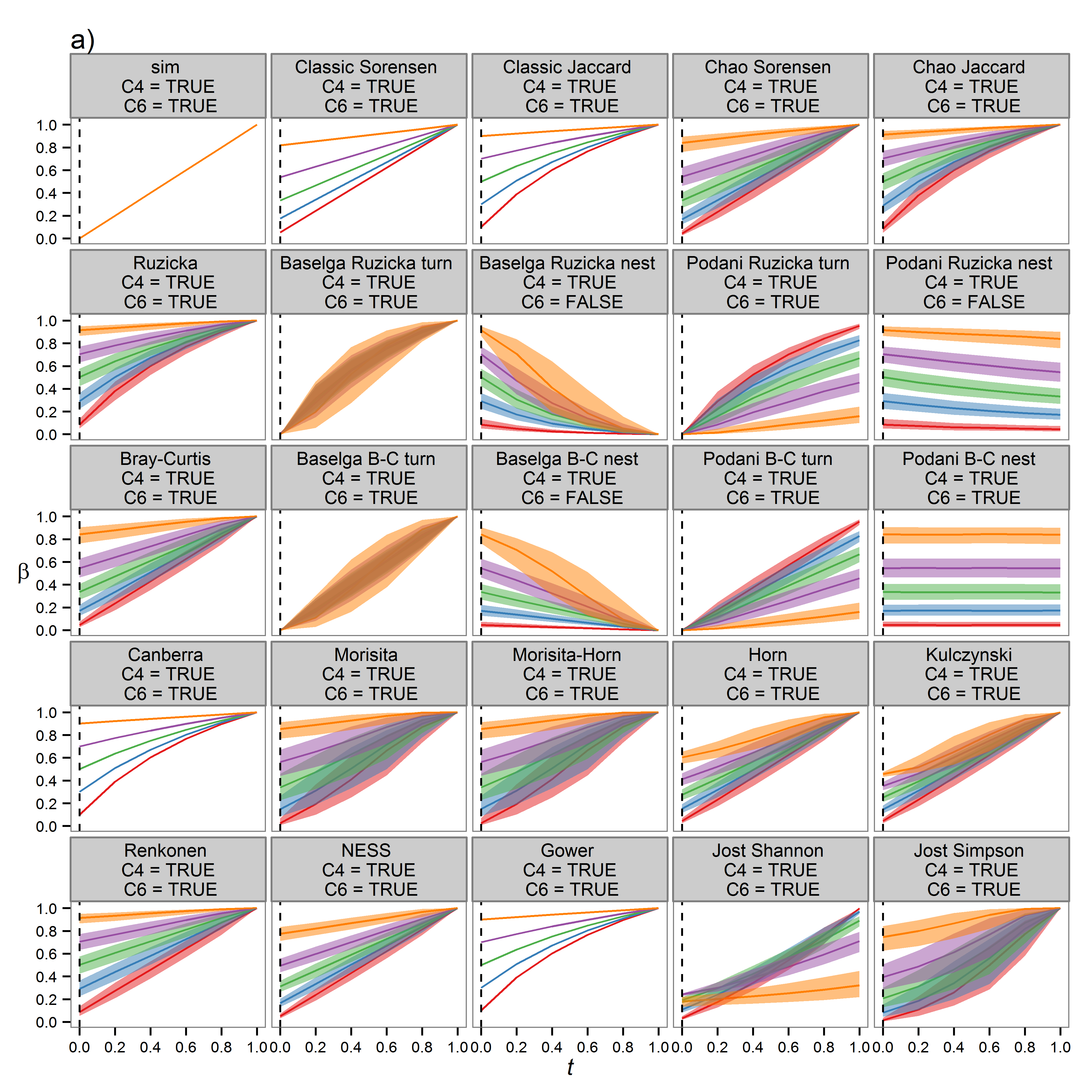


Fig. S4 Effect of species turnover on *β*-diversity for a) 25 metrics with a fixed upper limit b) 8 metrics without maxima. Solid lines and shaded areas are the median and interquartile range, respectively, of *β* based on 10000 simulations at each level of species turnover, *t*. Metrics are scored as TRUE or FALSE for desirable property C4, minimum of zero and positive and C6, monotonic increase with species turnover. Vertical dashed black lines intersect the reference values of median *β* at *t*=0 (no species turnover).


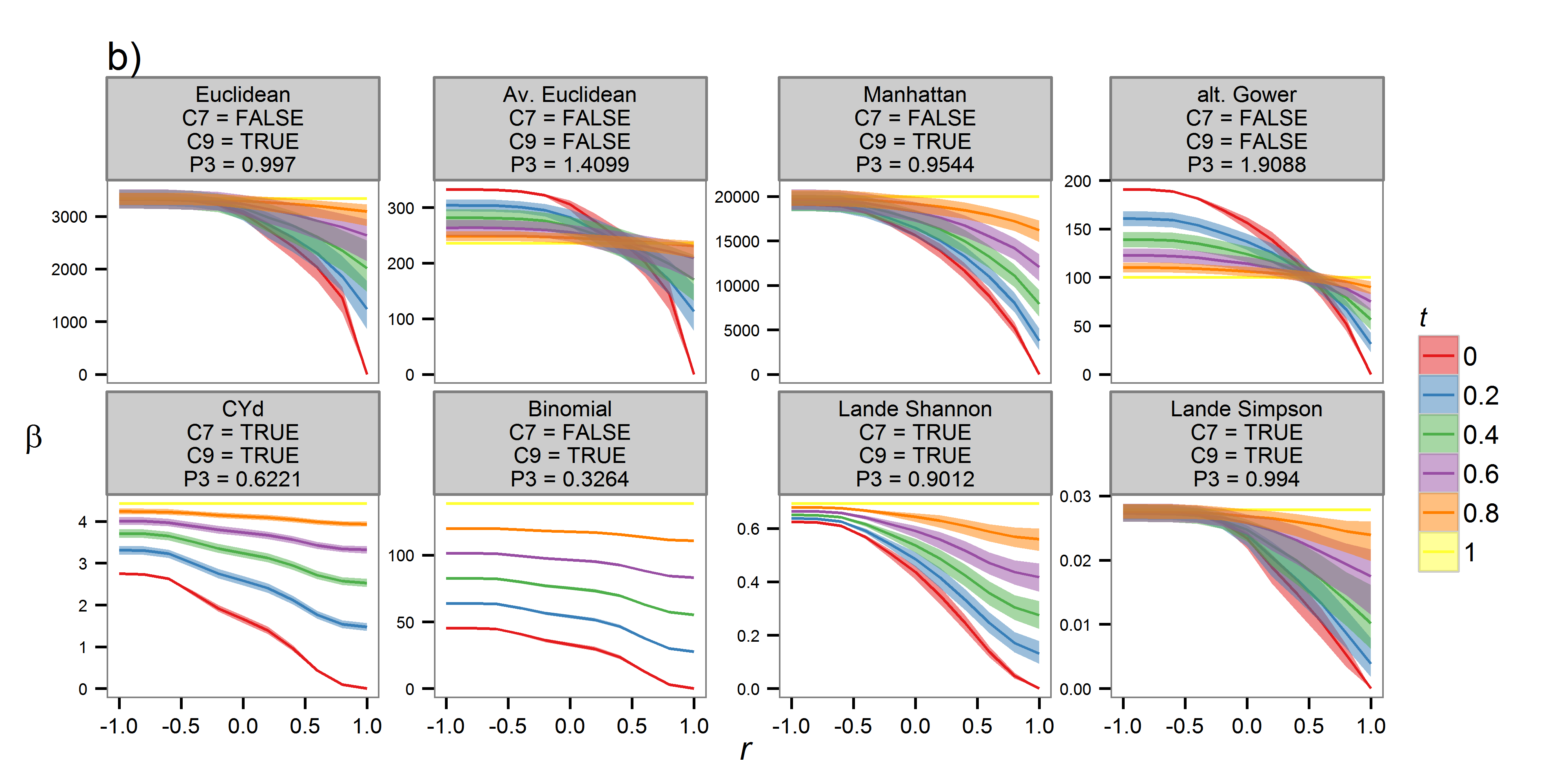

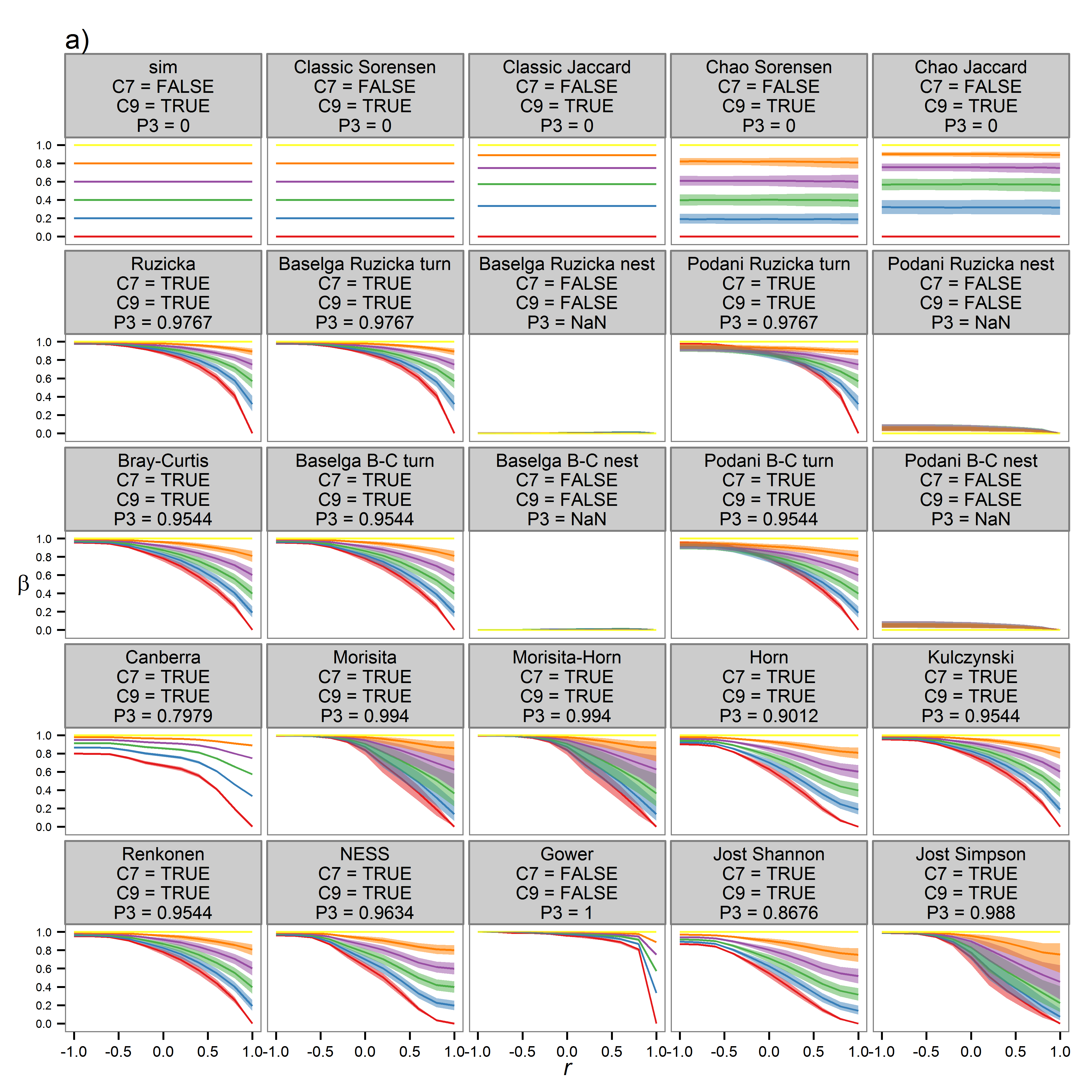


Fig. S5 Effect of decoupling of species ranks on *β*-diversity for a) 25 metrics with defined maxima and minima b) 8 metrics with no defined maxima. Solid lines and shaded areas are the median and interquartile range, respectively, of *β* based on 10000 simulations at each unique combination of species turnover, *t*, and partial correlation between ranks, *r.* Metrics are scored for desirable properties C7, monotonic increase with decoupling of species ranks and C9, extreme decoupling of species ranks (partial correlation = -1) is less than *β* for complete species turnover. Metrics are also scored for P3, relative sensitivity to decoupling of species ranks and species turnover. Vertical dashed black lines intersect the reference values of median *β* at *r* = 1 (perfect correlation between ranks).


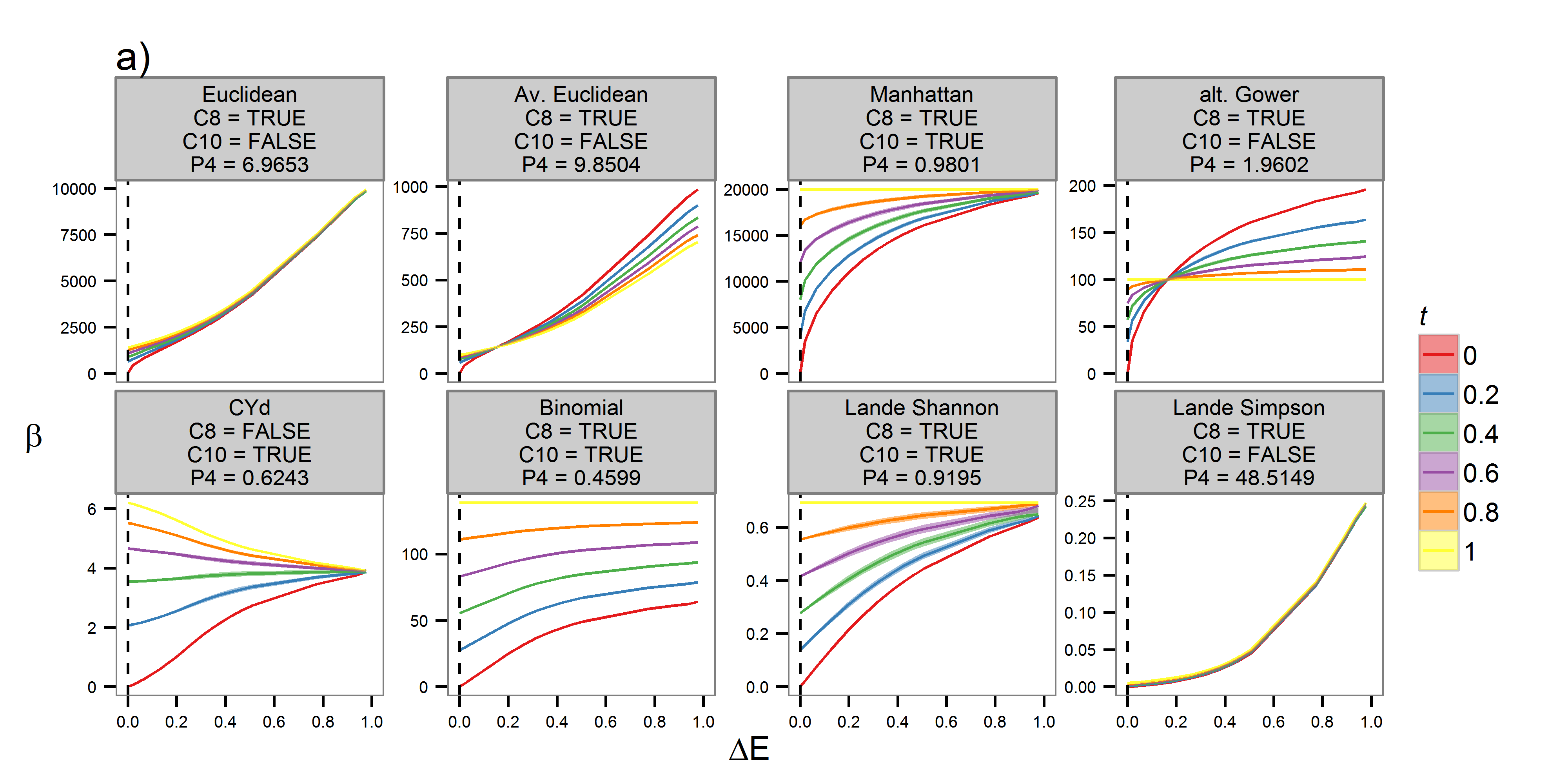

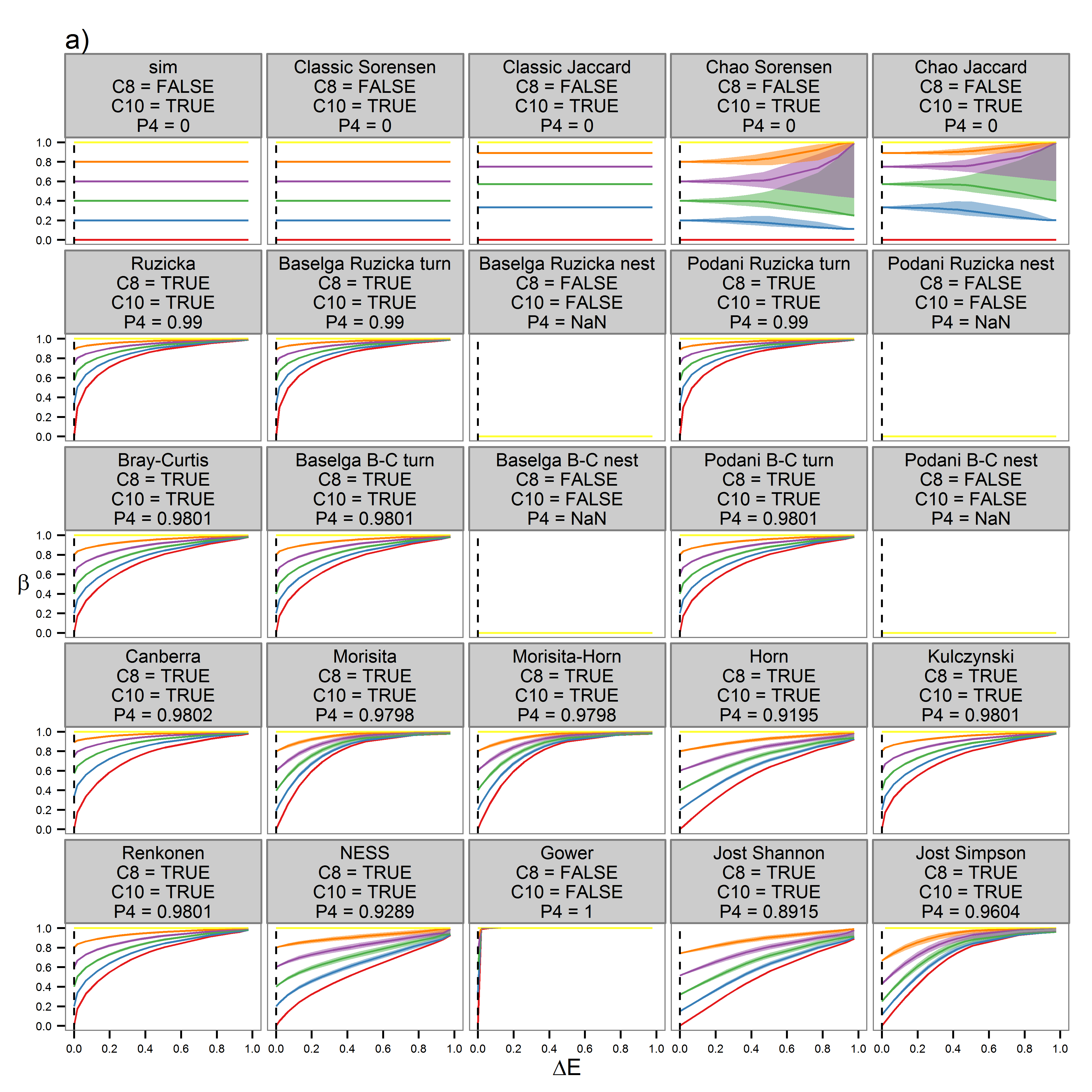


Fig. S6 Effect of evenness differences on *β*-diversity for a) 25 metrics with fixed upper limits b) 8 metrics with no maxima. Solid lines and shaded areas are the median and interquartile range, respectively, of *β* based on 10000 simulations at each unique combination of species turnover, *t*, and difference in evenness, Δ*E*. Metrics are scored for desirable properties C8, monotonic increase with evenness differences, and C10, median *β* under extreme evenness differences (Δ*E* ~ 1) is less than median *β* when species turnover is complete, (*t*=1). Metrics are also scored for P4, relative sensitivity to evenness differences and species turnover components of *β*. Vertical dashed black lines intersect the reference values of median *β* at Δ*E*=0 (no evenness differences).


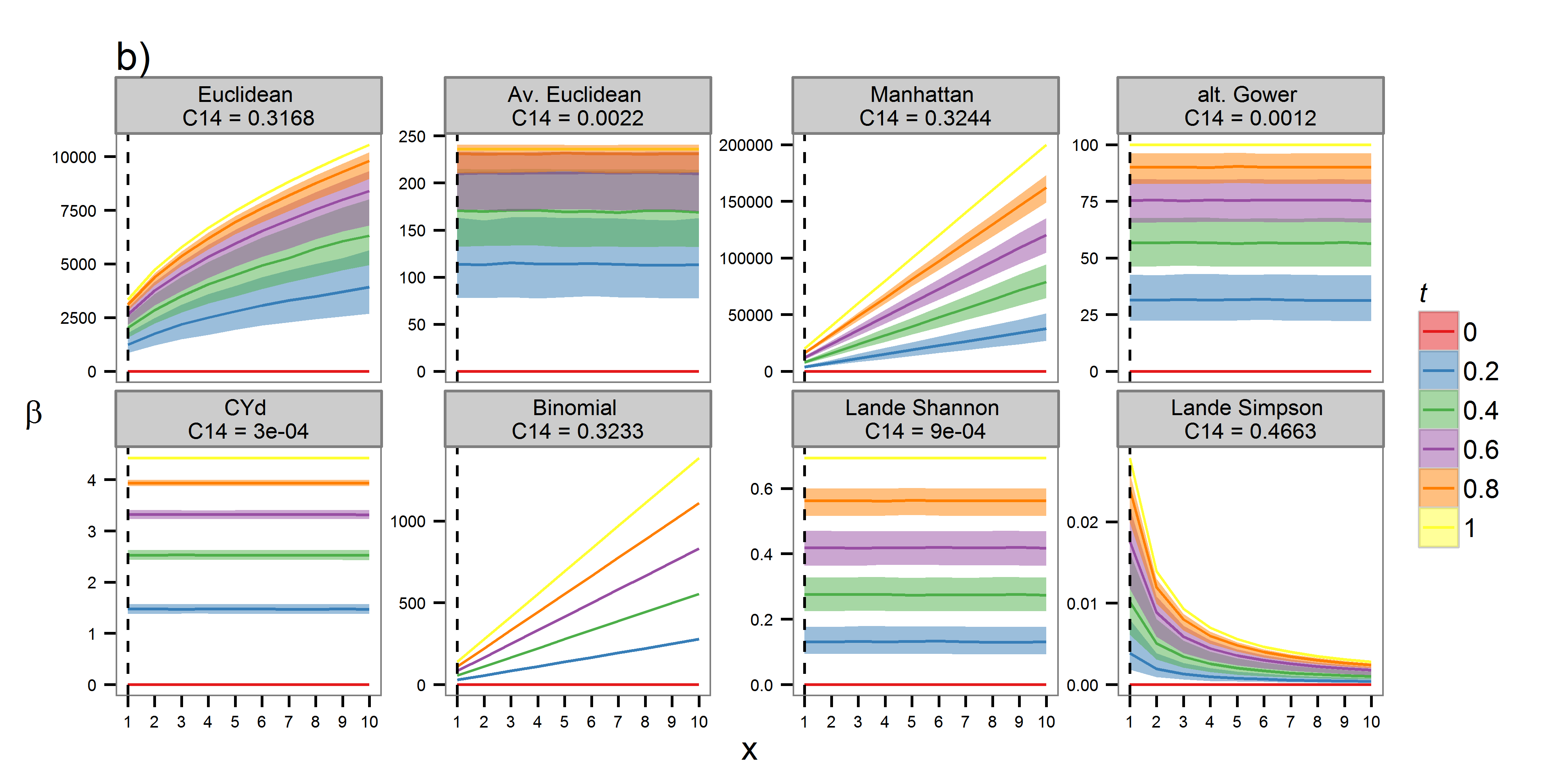

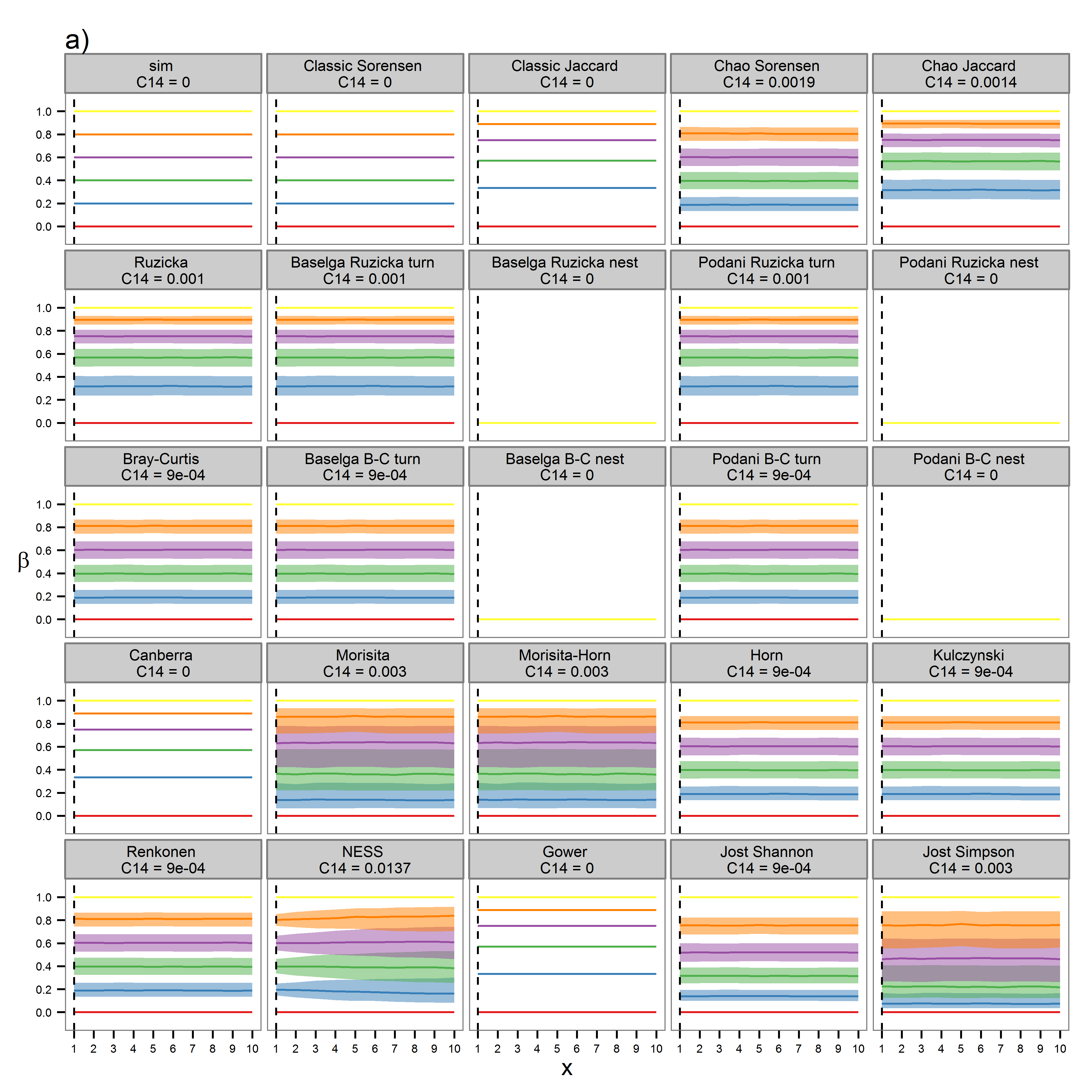


Fig. S7 Effect of species replication (pooling *x* identical subsets of an assemblage pair) on *β*-diversity for a) 25 metrics with fixed upper limits b) 8 metrics with no maxima. Solid lines and shaded areas are the median and interquartile range, respectively, of *β* based on 10000 simulations at each unique combination of species turnover, *t*, and number of species replication events, *x*. Metrics are scored for desirable property C14, independence of species replication. Vertical dashed black lines intersect the reference values of median *β* at *x*=1 (no species replication).


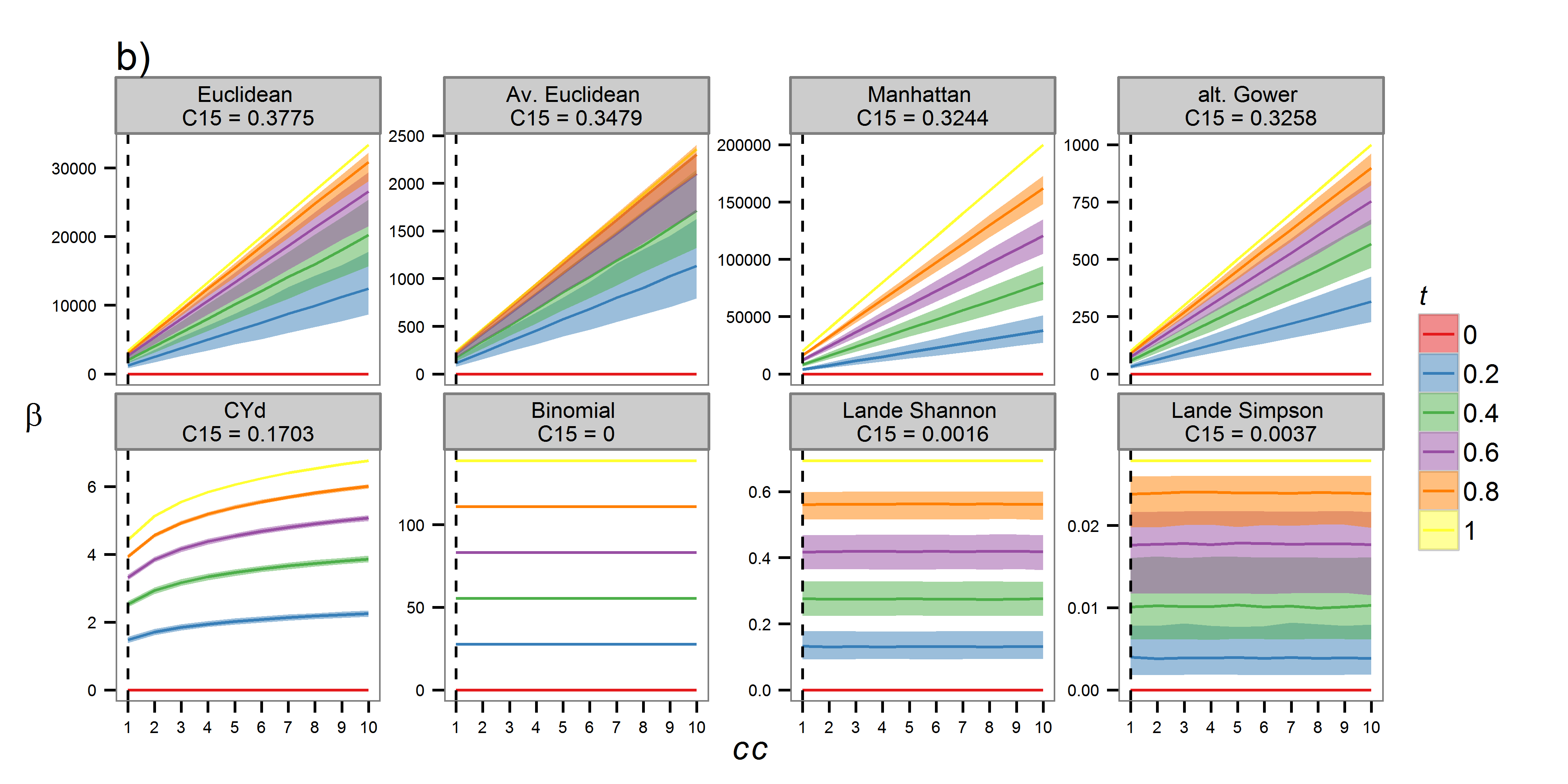

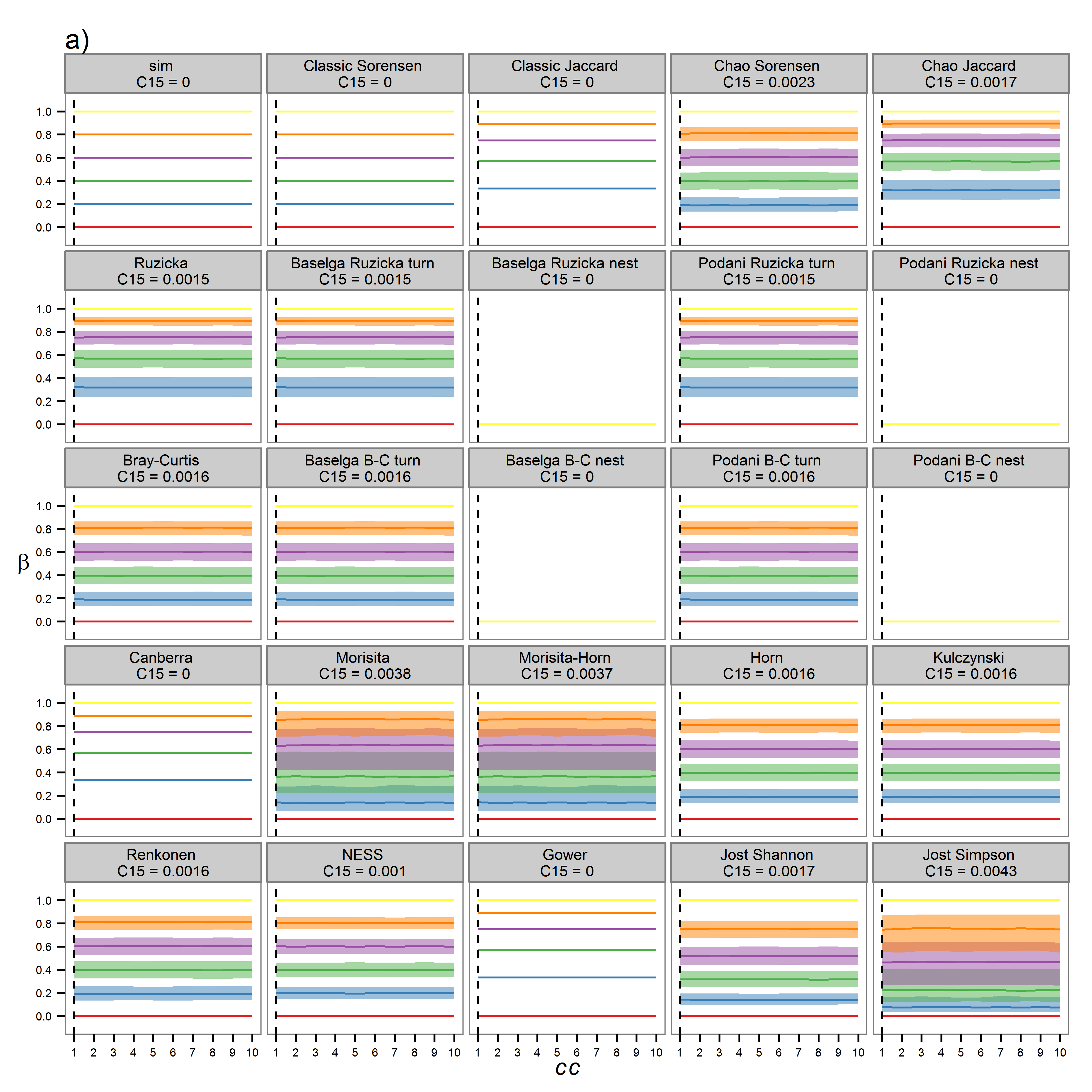


Fig. S8 Effect of units used to measure abundance on *β*-diversity for a) 25 metrics with fixed upper limits b) 8 metrics with no maxima. Solid lines and shaded areas are the median and interquartile range, respectively, of *β* based on 10000 simulations at each unique combination of species turnover, *t*, and a constant factor, *cc*, by which abundances in both assemblages are multiplied. Metrics are scored for desirable property C15, independence of measurement units. Vertical dashed black lines intersect the reference values of median *β* at *cc*=1 (no change in measurement units).


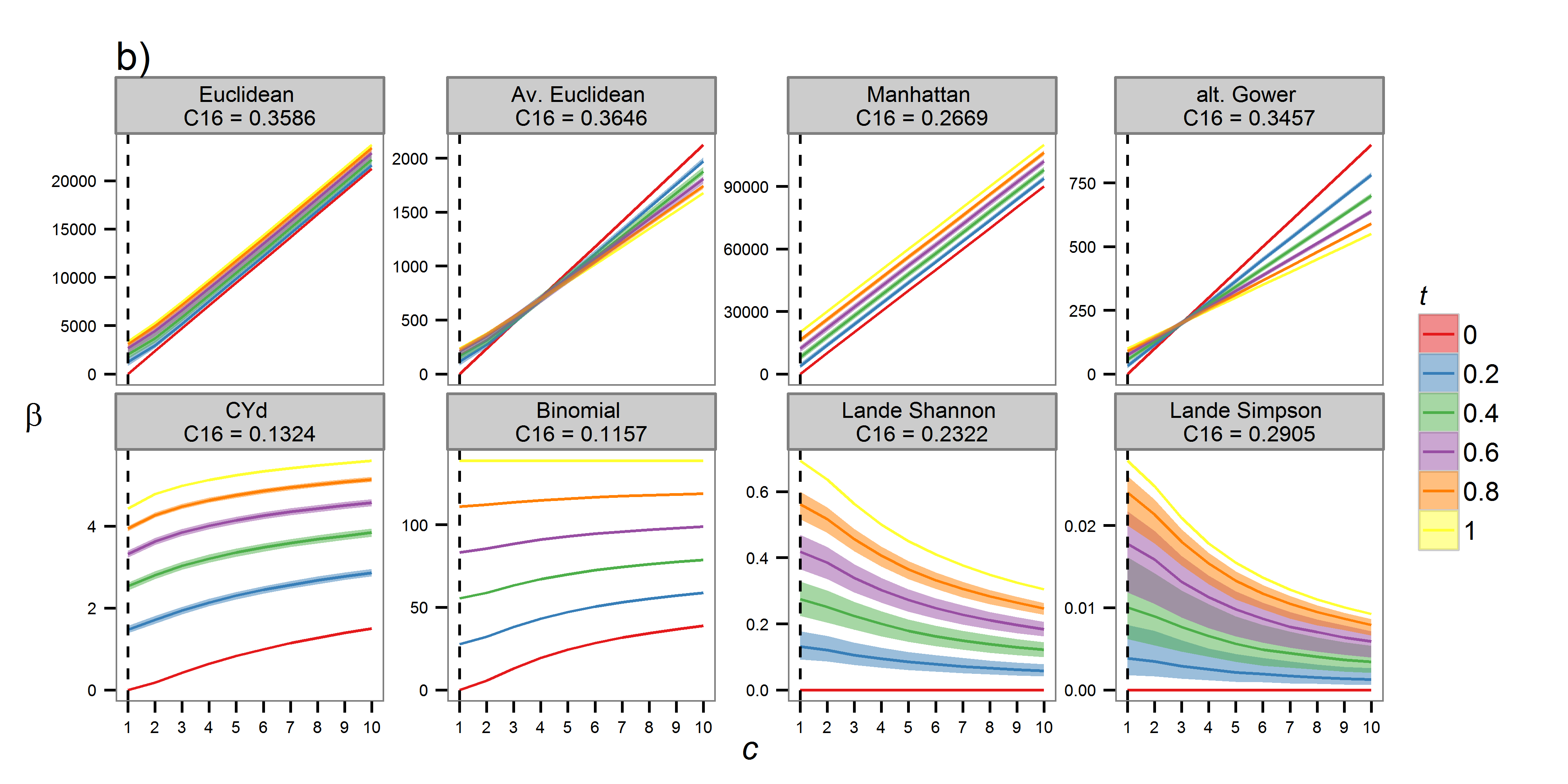

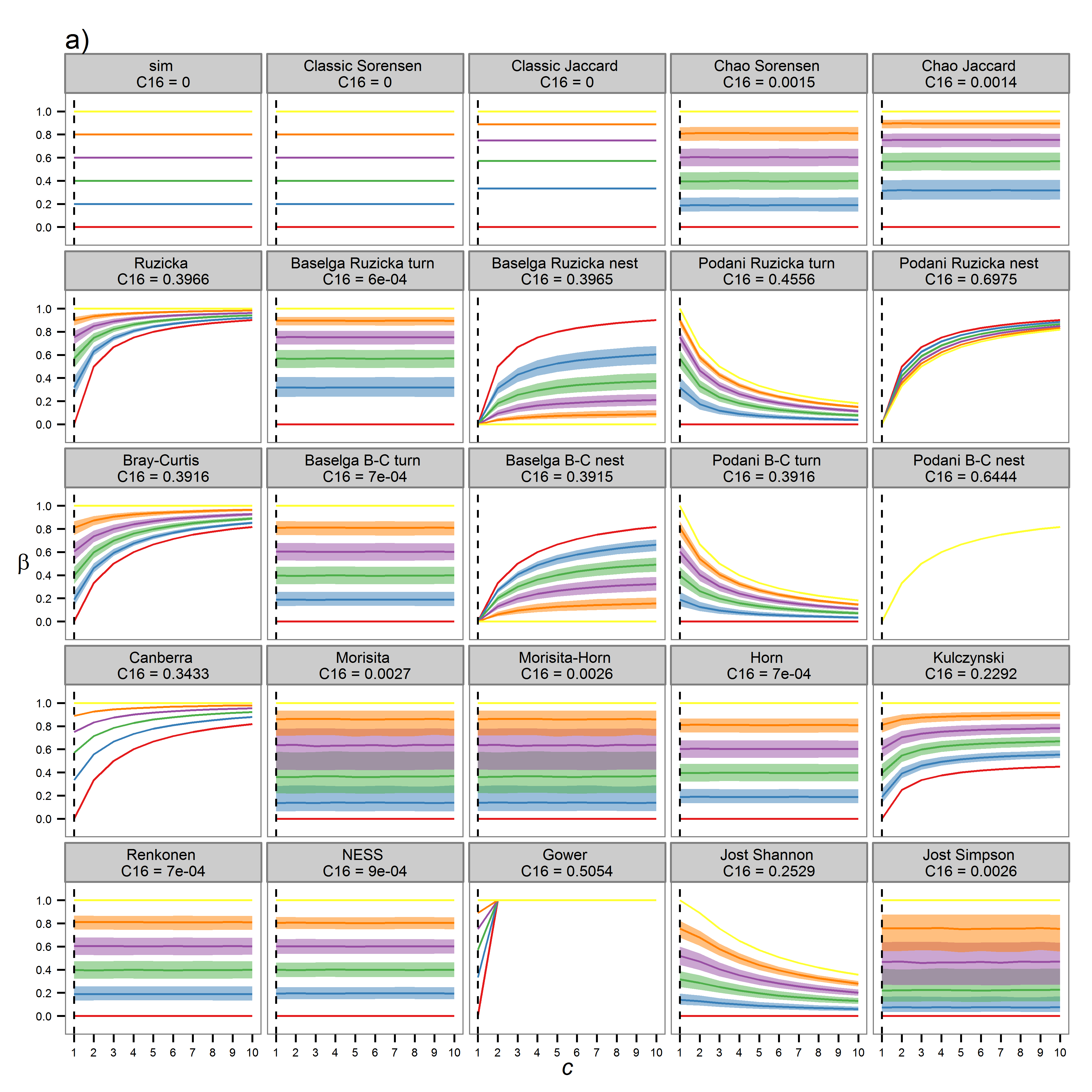


Fig. S9 Effect of differences in abundance between two assemblages on *β*-diversity for a) 25 metrics with fixed upper limits b) 8 metrics with no maxima. Solid lines and shaded areas are the median and interquartile range, respectively, of *β* based on 10000 simulations at each unique combination of species turnover, *t*, and a constant factor, *c*, by which abundances in one assemblage are multiplied. Metrics are scored for desirable property C16, independence of differences in abundance. Vertical dashed black lines intersect the reference values of median *β* at *c*=1 (no abundance differences).


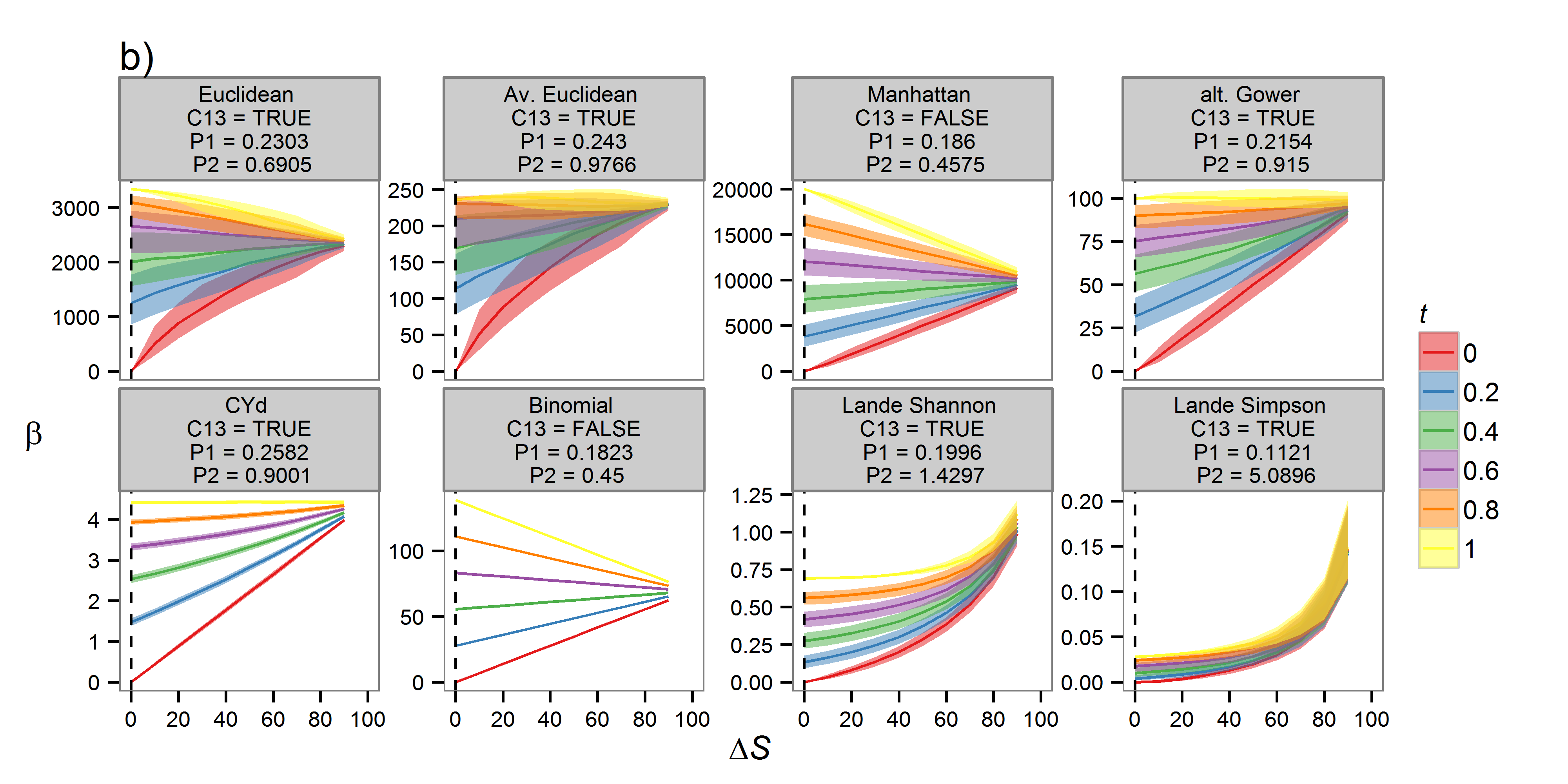

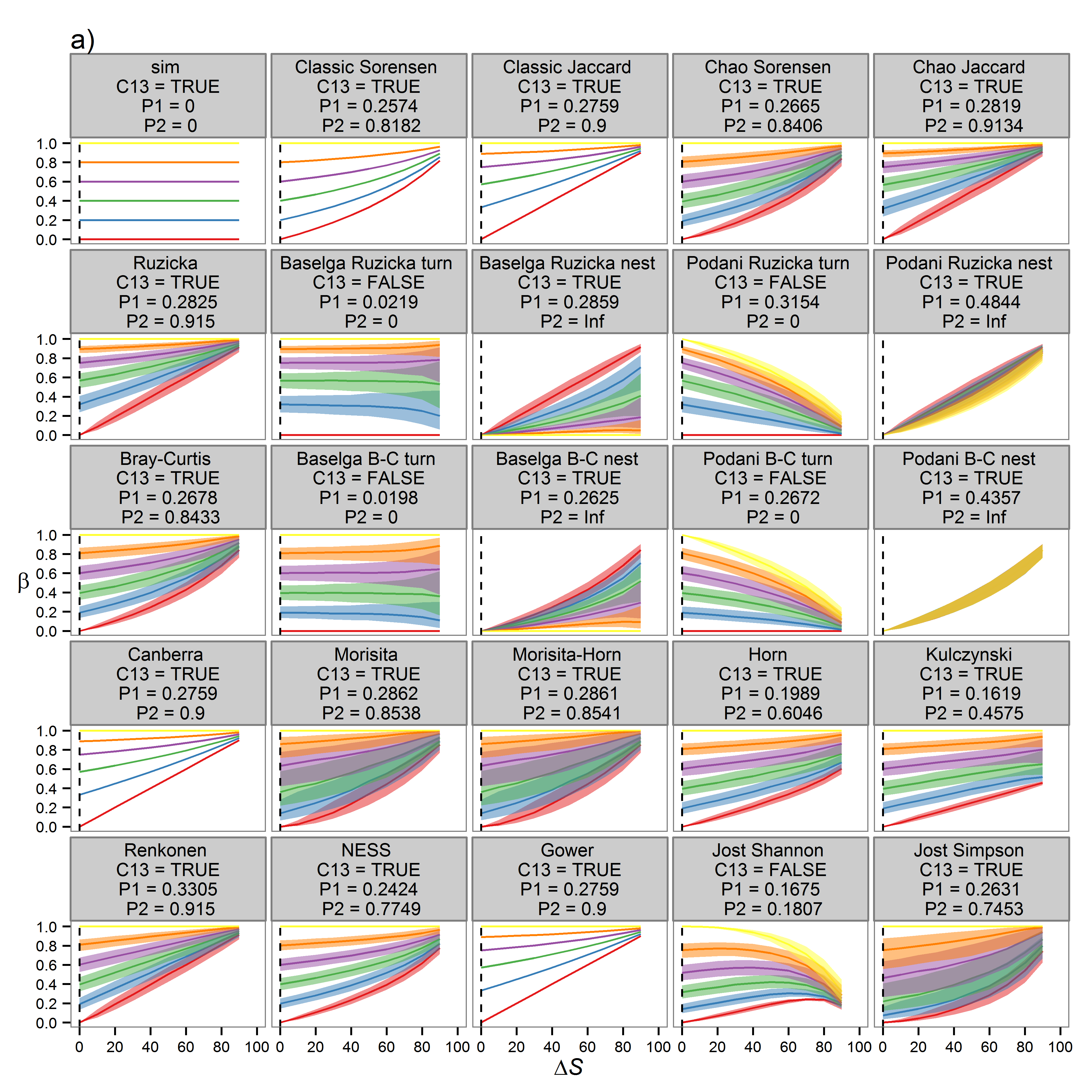


Fig. S10 Effect of nestedness on the value of *β-*diversity for a) 25 metrics with fixed upper limits and b) 8 metrics with no maxima. Solid lines and shaded areas are the median and interquartile range, respectively, of *β* based on 10000 simulations at each unique combination of species turnover, *t*, and difference in species richness, *ΔS*. Metrics are scored for desirable property C13, *β* does not decrease in a series of nested assemblages and personality traits P1, sensitivity to nestedness, and P2 relative sensitivity to nestedness and turnover. Vertical dashed black lines intersect the reference values of median *β* at *ΔS*=0 (no differences in species richness).

Fig. S11 Sensitivity to turnover in rare versus common species by a) 25 metrics with fixed upper limits b) 8 metrics with no maxima. Solid lines are the value of *β* when a single species with relative abundance, *n*, is turned over. Metrics are scored for personality trait P5, relative sensitivity to turnover in rare versus common species. A value of 1 indicates that a metric weights turnover in rare and common species equally, while a value of less than one indicates rare species contribute less to the value of *β.* A value of zero indicates a metric is almost completely insensitive to turnover in rare species.


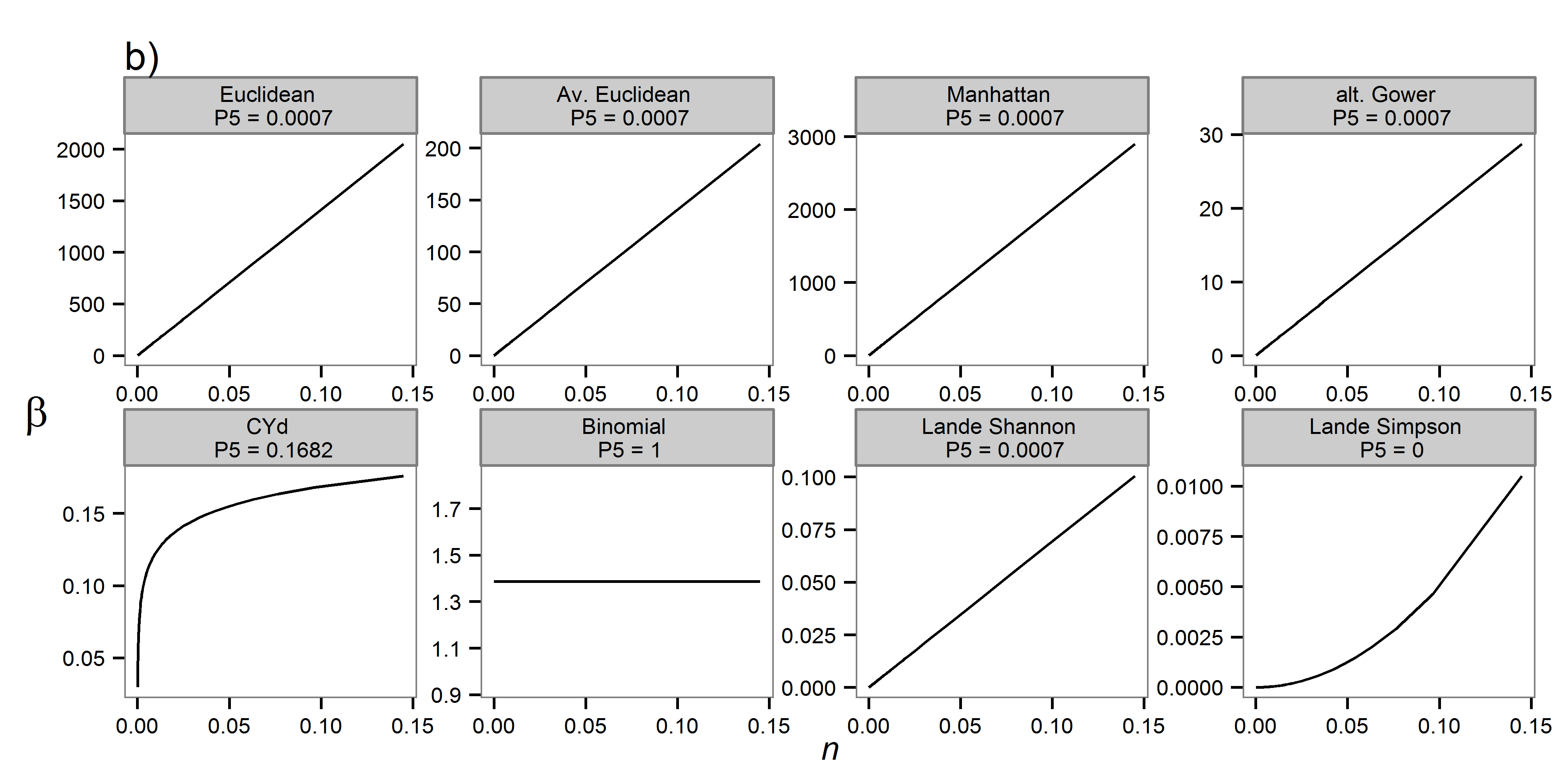

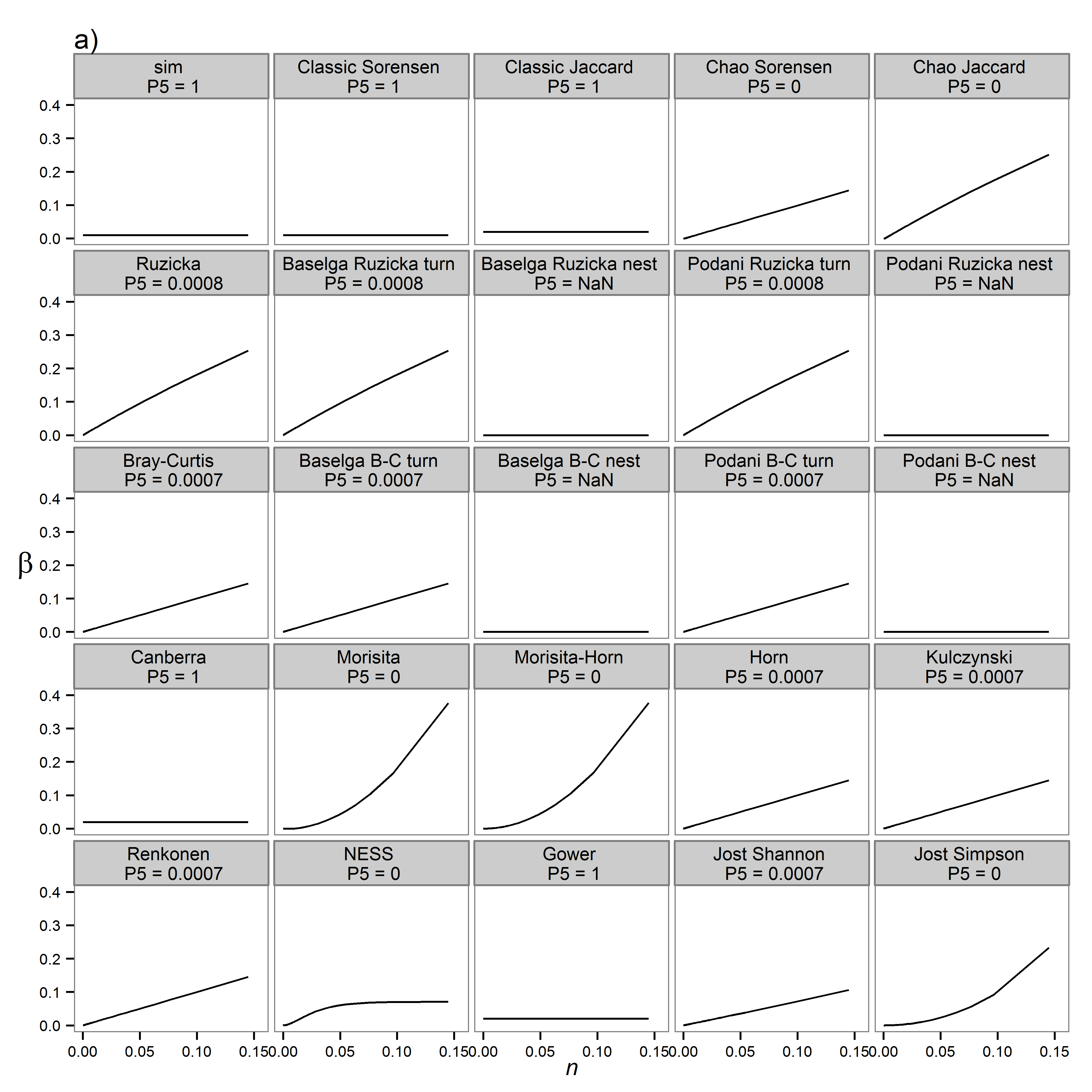


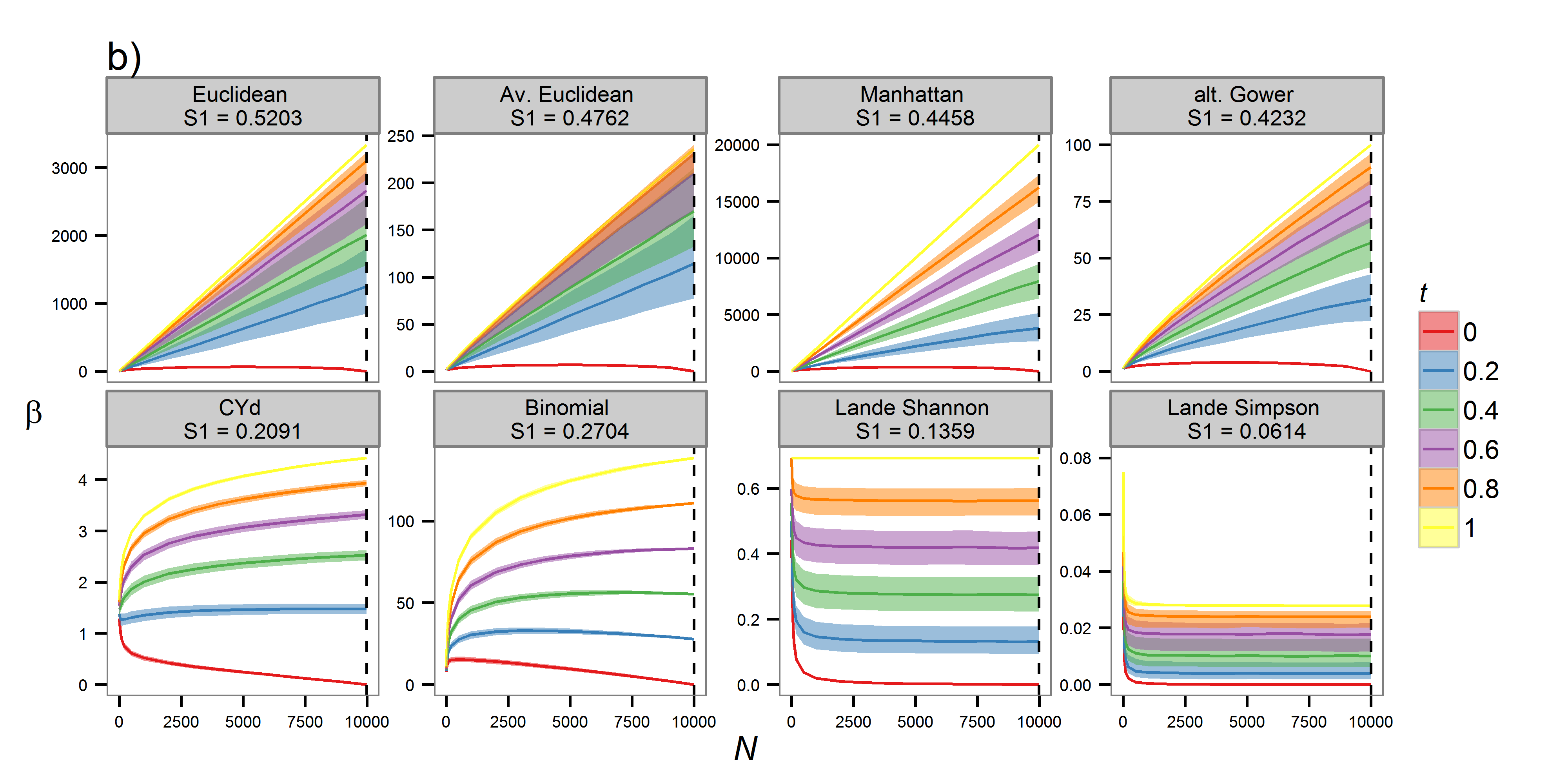

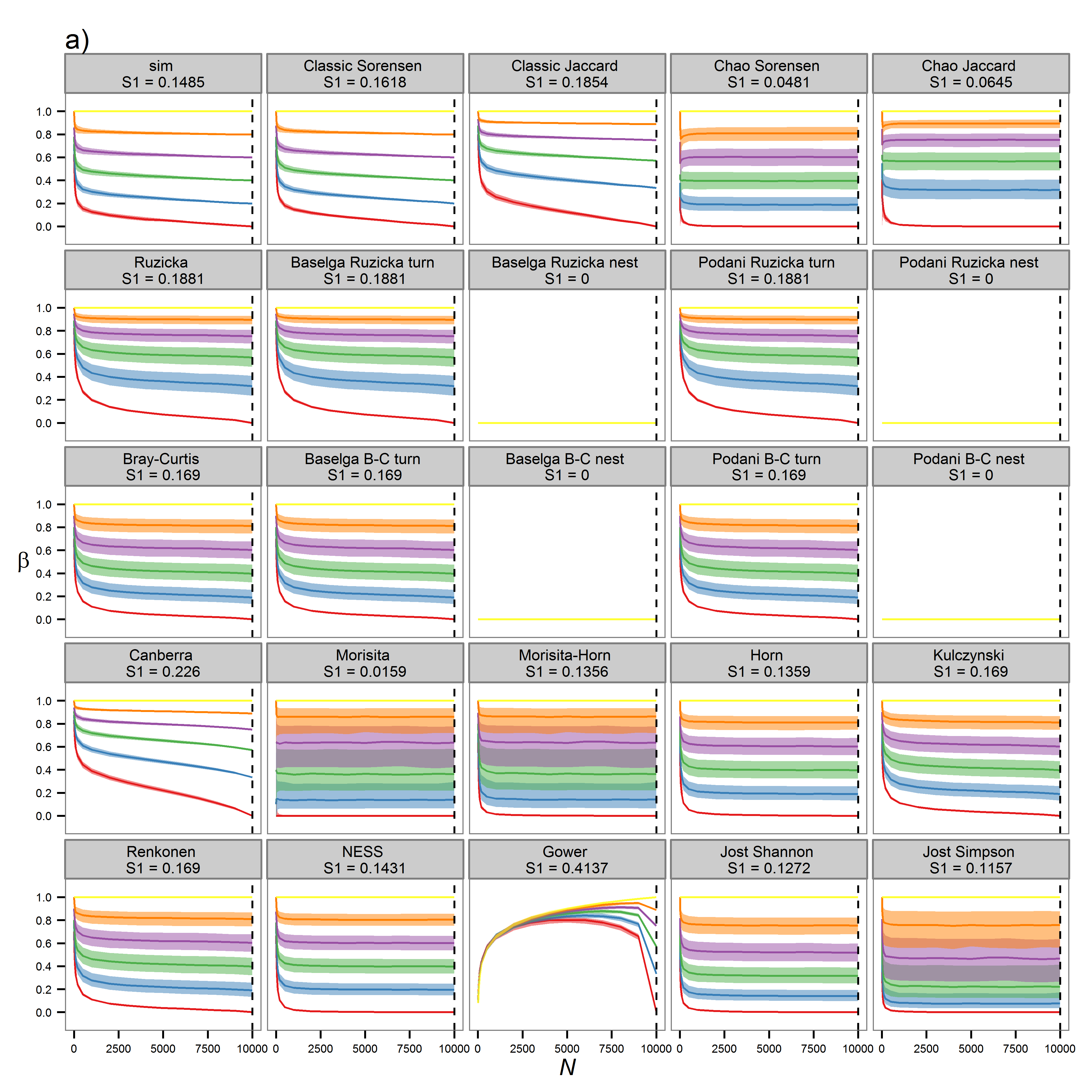


Fig. S12 Effect of sample size on the value of *β* for a) 25 metrics with defined minima and maxima and b) 8 metrics with no defined maxima. Solid lines and shaded areas are the median and interquartile range, respectively, of *β* for 10000 simulations at each unique combination of species turnover, *t,* and sample size, *N*. Metrics are scored for desirable property S1, independence of sample size. Vertical dashed black lines intersect the reference values of median *β* at *N*=10000 (fully censused assemblages).


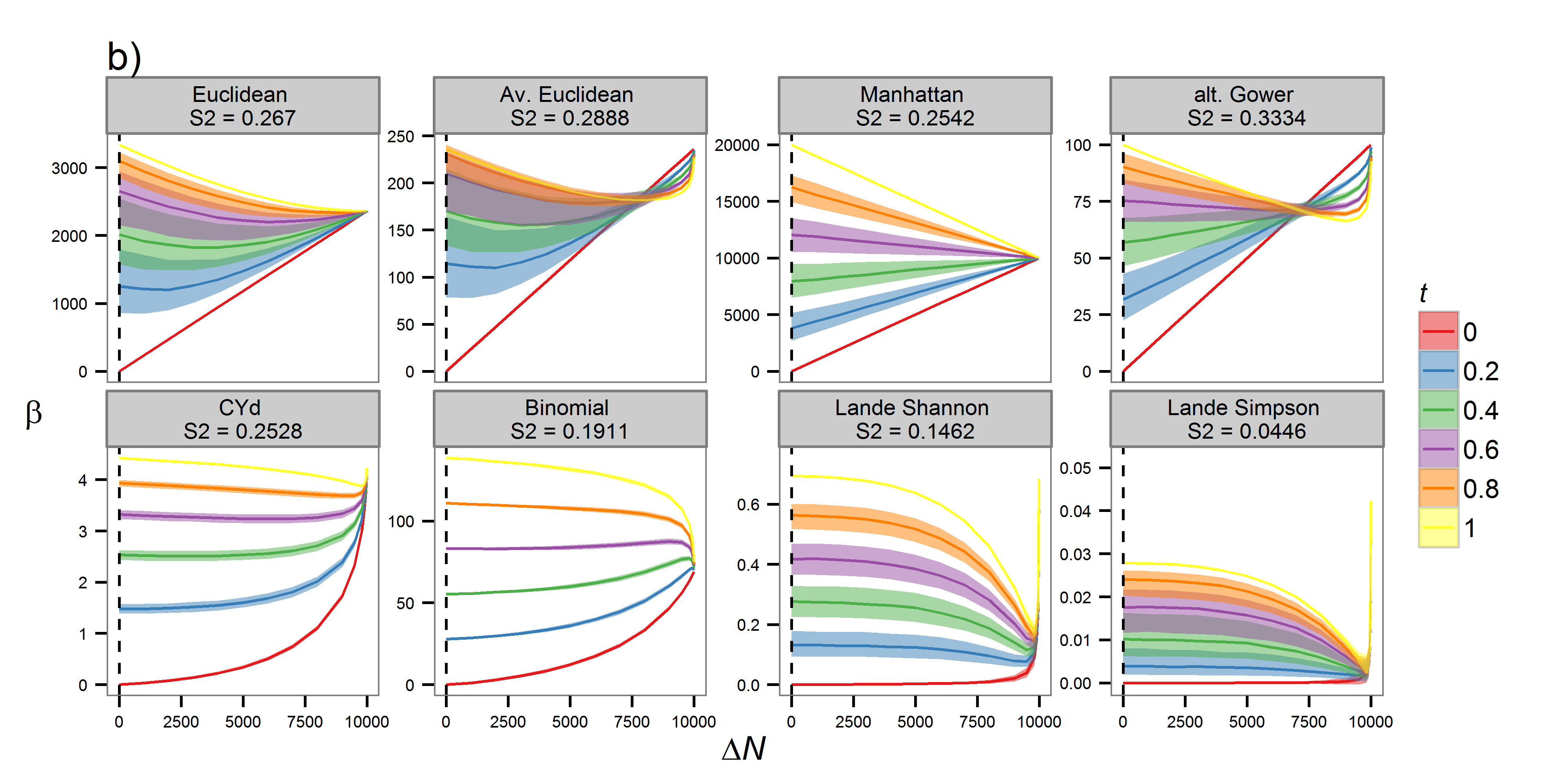

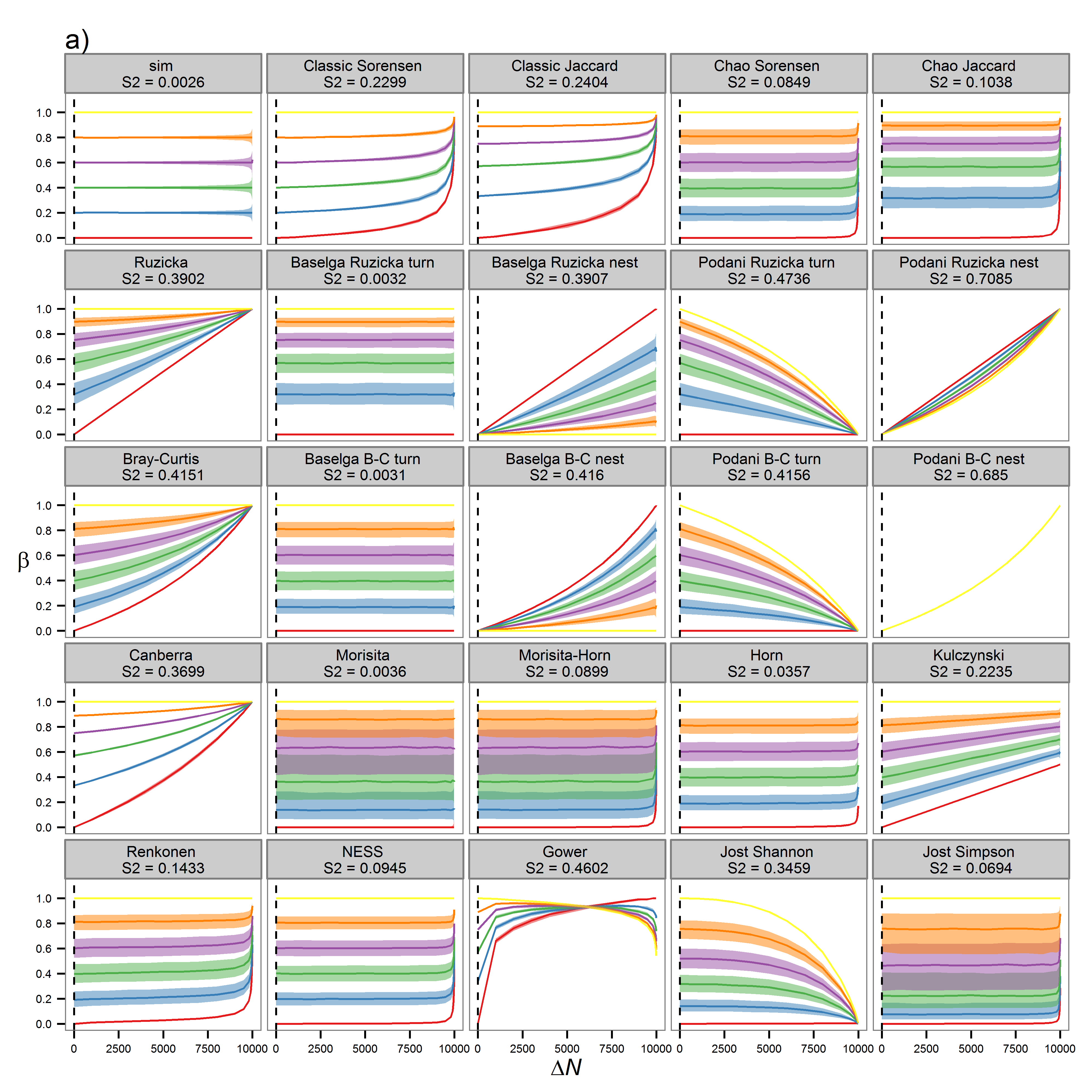


Fig. S13 Effect of unequal sample size on the value *β* for a) 25 metrics with fixed upper limits b) 8 metrics with no maxima. Solid lines and shaded areas are the median and interquartile range, respectively, of *β* for 10000 simulations at each unique combination of species turnover, *t,* and sample size difference, *ΔN*. Metrics are ordered by their scores for desirable property S2, unbiased by unequal sample size. Vertical dashed black lines intersect the reference values of median *β* at *ΔN*=0 (equal sample sizes).


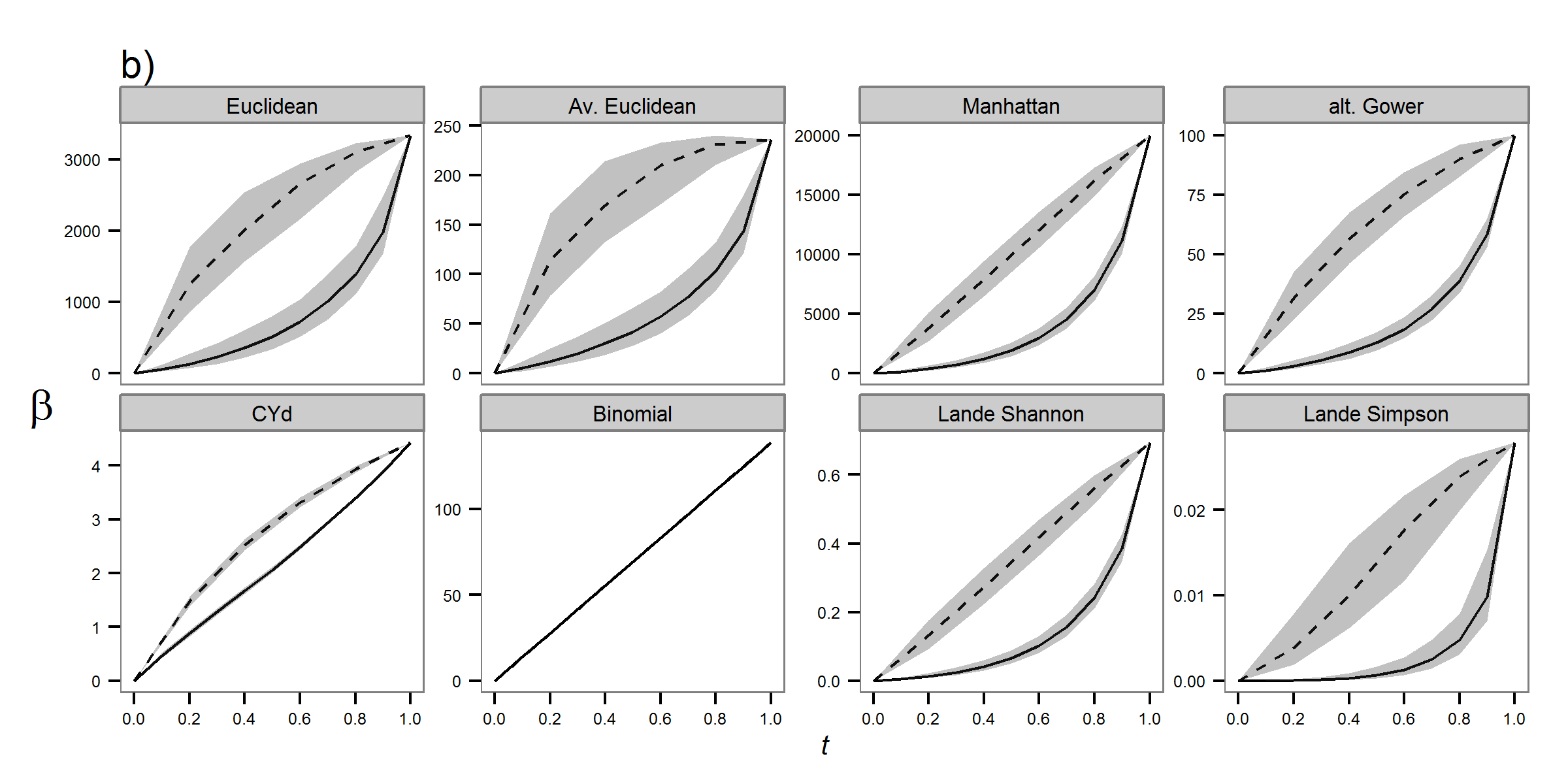

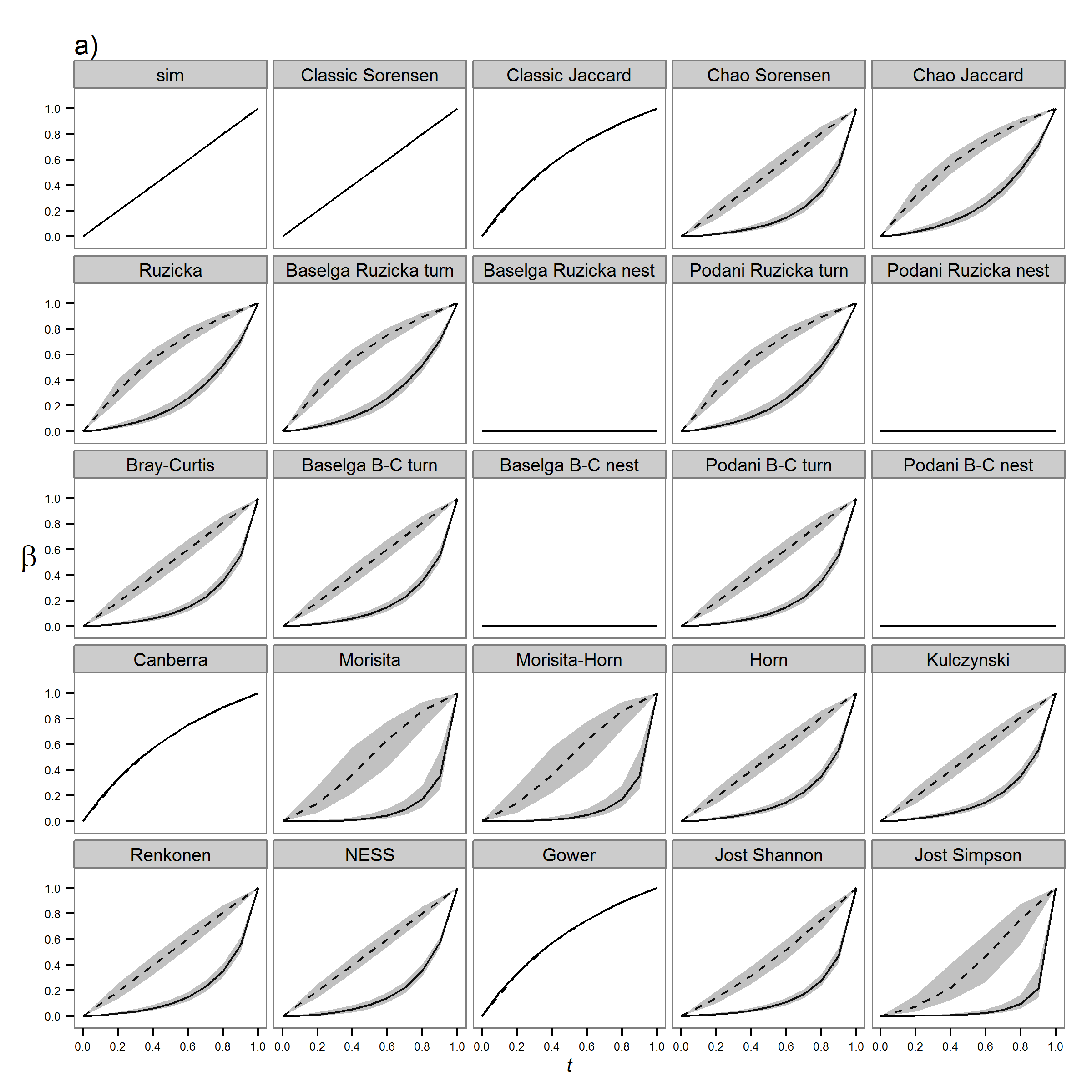


Fig. S14 Effect of species turnover on *β*-diversity under a positive occupancy-abundance relationship (ONR) for a) 25 metrics with fixed upper limits b) 8 metrics with no maxima. Dashed black lines are the median of *β* under random species turnover. Solid black lines are the median of *β* when the probability of a species being turned over is inversely proportional to its relative abundance (a positive ONR with exponent 0.65). Shaded areas are the interquartile ranges of *β*. Median and interquartile range values are based on 10000 simulations at each level of species turnover, *t*.


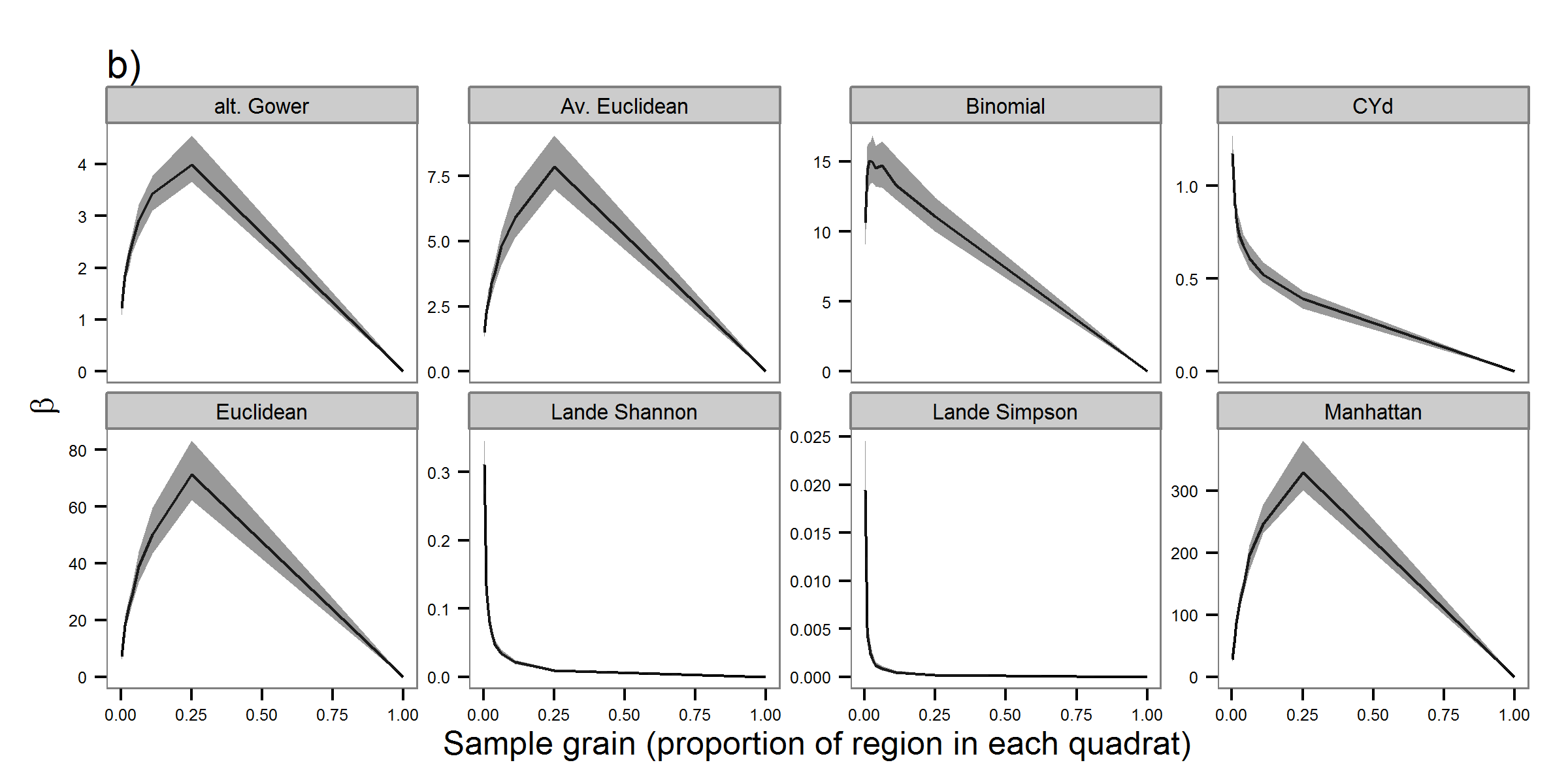

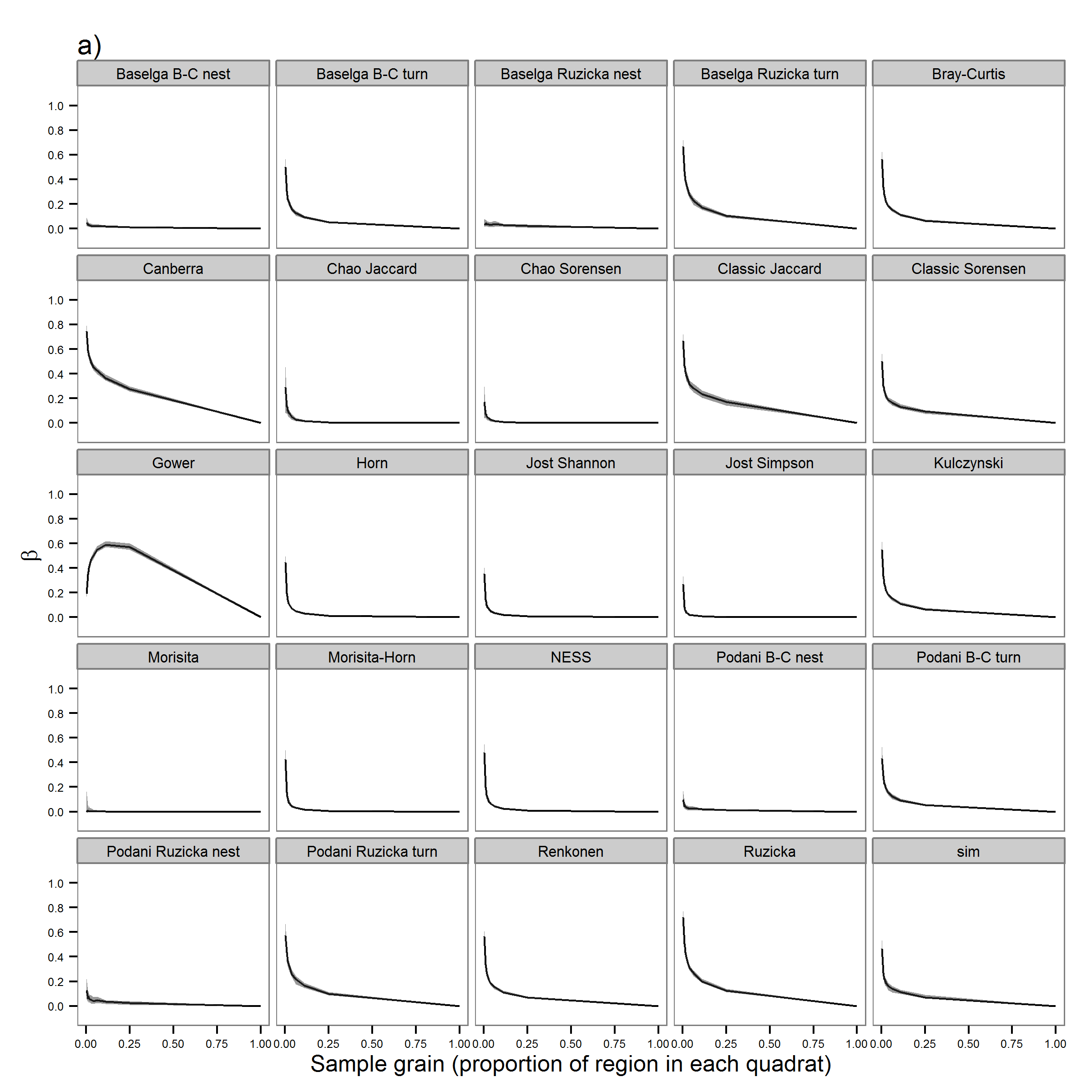


Fig. S15 Effect of the spatial grain of sampling on *β*-diversity for a) 25 metrics with fixed upper limits b) 8 metrics with no maxima. Solid lines and shaded areas are the median and interquartile, respectively, of *β* based on 100 simulations at each spatial grain. Individuals of each species are assumed to be distributed according to an inhomogeneous Poisson point process (the Thomas process). Quadrat pairs are equally sized and their position is sampled at random from within the simulated study region.

**
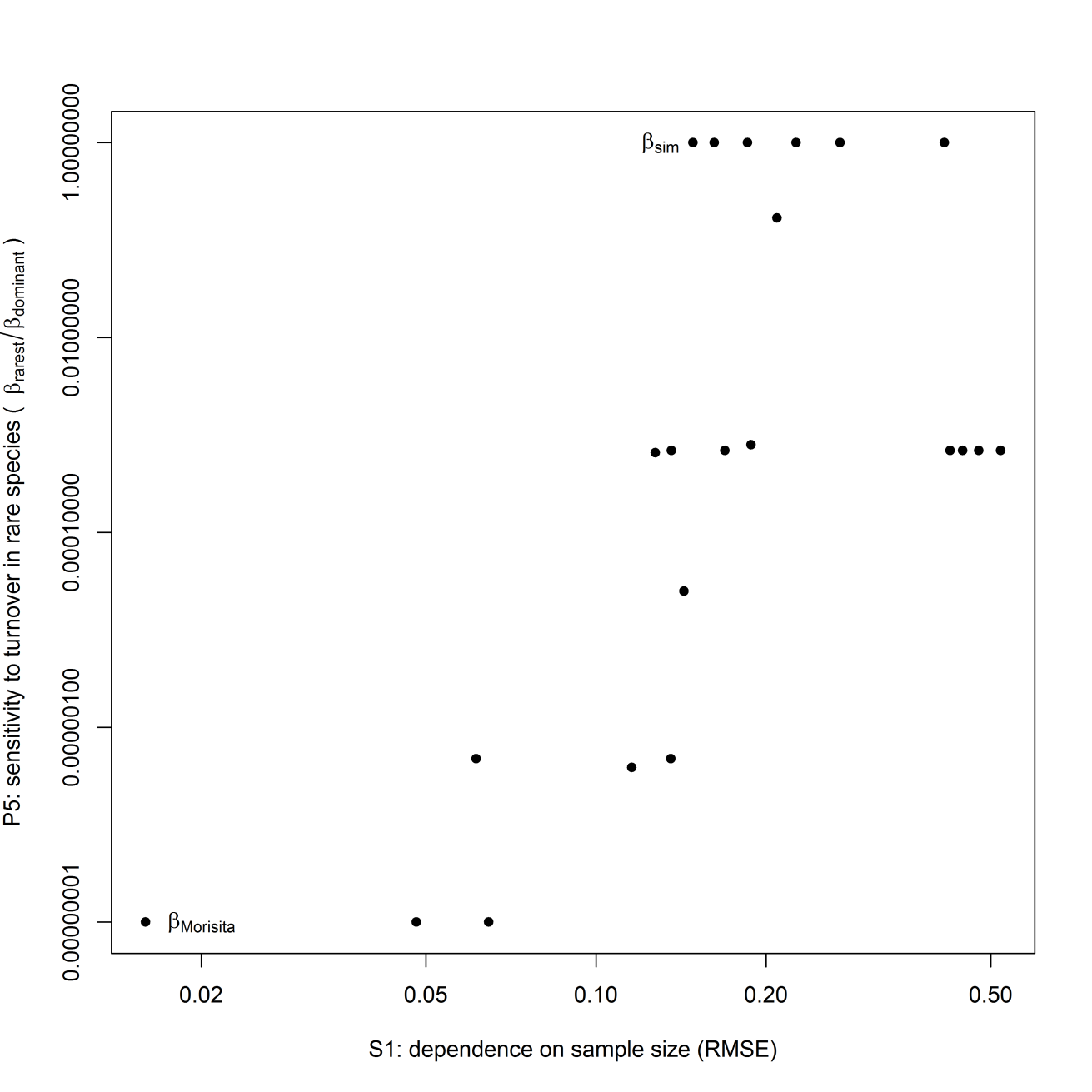
**

Fig. S16 Trade-off between sampling property S1 (independence of sample size) and personality property P5 (relative sensitivity to turnover in rare and common species). Black dots are the 29 metrics tested against the two properties. *β*_Morisita_ and *β*_sim_ represent two extremes of this trade-off.

**Table S5** Rows contain the number of properties (including both conceptual and sampling properties) for which a metric was out-performed or equalled by another metric. C4, C5 and C11 were excluded from these analyses as all metrics met these criteria. A value of 15 indicates a metric is Pareto-dominated (outperformed or equalled across all desirable properties).

|  | sim | Classic Sorensen | Classic Jaccard | Chao Sorensen | Chao Jaccard | Ruzicka | Baselga R turn | Podani R turn | Bray-Curtis | Baselga B-C turn | Podani B-C turn | Canberra | Morisita | Morisita-Horn | Horn | Kulczynski | Renkonen | NESS | Gower | Jost Shannon | Jost Simpson | Euclidean | Av. Euclidean | Manhattan | alt. Gower | CYd | Binomial | Lande Shannon | Lande Simpson |
| --- | --- | --- | --- | --- | --- | --- | --- | --- | --- | --- | --- | --- | --- | --- | --- | --- | --- | --- | --- | --- | --- | --- | --- | --- | --- | --- | --- | --- | --- |
| sim | NA | 13 | 11 | 8 | 7 | 7 | 6 | 6 | 8 | 7 | 7 | 10 | 9 | 9 | 9 | 8 | 8 | 8 | 7 | 7 | 8 | 4 | 4 | 5 | 4 | 6 | 6 | 7 | 6 |
| Classic Sorensen | 15 | NA | 11 | 9 | 8 | 7 | 7 | 6 | 8 | 8 | 7 | 10 | 10 | 10 | 10 | 9 | 9 | 9 | 7 | 7 | 9 | 4 | 4 | 5 | 4 | 6 | 7 | 8 | 7 |
| Classic Jaccard | 15 | 15 | NA | 10 | 10 | 9 | 9 | 8 | 10 | 10 | 9 | 12 | 11 | 11 | 11 | 11 | 11 | 10 | 8 | 8 | 11 | 5 | 4 | 5 | 5 | 7 | 7 | 8 | 7 |
| Chao Sorensen | 13 | 12 | 11 | NA | 11 | 9 | 10 | 8 | 11 | 12 | 10 | 10 | 10 | 8 | 13 | 11 | 12 | 11 | 8 | 8 | 8 | 5 | 4 | 6 | 5 | 7 | 6 | 9 | 7 |
| Chao Jaccard | 14 | 13 | 11 | 12 | NA | 10 | 11 | 9 | 12 | 13 | 11 | 10 | 11 | 10 | 14 | 12 | 13 | 13 | 9 | 10 | 10 | 5 | 4 | 7 | 6 | 7 | 7 | 10 | 8 |
| Ruzicka | 13 | 13 | 11 | 10 | 9 | NA | 14 | 12 | 13 | 13 | 12 | 12 | 12 | 12 | 14 | 14 | 14 | 13 | 6 | 12 | 12 | 5 | 5 | 7 | 6 | 8 | 7 | 11 | 8 |
| Baselga R turn | 13 | 12 | 10 | 8 | 7 | 13 | NA | 13 | 12 | 13 | 12 | 10 | 10 | 10 | 12 | 12 | 12 | 11 | 6 | 11 | 10 | 3 | 3 | 6 | 4 | 6 | 6 | 9 | 6 |
| Podani R turn | 13 | 13 | 11 | 10 | 9 | 15 | 15 | NA | 14 | 14 | 14 | 12 | 12 | 12 | 14 | 14 | 14 | 13 | 7 | 13 | 12 | 5 | 5 | 8 | 6 | 8 | 8 | 11 | 8 |
| Bray-Curtis | 12 | 12 | 10 | 8 | 7 | 9 | 9 | 7 | NA | 14 | 13 | 12 | 11 | 11 | 15 | 15 | 15 | 12 | 5 | 9 | 10 | 5 | 5 | 7 | 5 | 8 | 6 | 12 | 8 |
| Baselga B-C turn | 12 | 11 | 9 | 6 | 5 | 8 | 9 | 8 | 13 | NA | 13 | 10 | 9 | 9 | 14 | 13 | 14 | 10 | 5 | 8 | 8 | 3 | 3 | 6 | 3 | 6 | 5 | 10 | 6 |
| Podani B-C turn | 12 | 12 | 10 | 8 | 7 | 9 | 10 | 8 | 15 | 15 | NA | 12 | 11 | 11 | 15 | 15 | 15 | 12 | 5 | 10 | 10 | 5 | 5 | 8 | 5 | 8 | 7 | 12 | 8 |
| Canberra | 13 | 13 | 13 | 9 | 9 | 10 | 11 | 9 | 10 | 11 | 9 | NA | 12 | 12 | 12 | 12 | 12 | 11 | 6 | 10 | 12 | 5 | 4 | 6 | 5 | 9 | 7 | 9 | 8 |
| Morisita | 11 | 10 | 9 | 9 | 8 | 10 | 11 | 9 | 11 | 12 | 10 | 10 | NA | 10 | 12 | 11 | 12 | 10 | 5 | 8 | 10 | 3 | 4 | 4 | 4 | 6 | 4 | 8 | 5 |
| Morisita-Horn | 11 | 10 | 9 | 11 | 9 | 10 | 11 | 9 | 11 | 12 | 10 | 10 | 13 | NA | 13 | 11 | 12 | 10 | 5 | 9 | 12 | 3 | 4 | 4 | 4 | 6 | 4 | 8 | 8 |
| Horn | 11 | 10 | 9 | 6 | 5 | 8 | 9 | 7 | 12 | 13 | 11 | 10 | 10 | 9 | NA | 12 | 13 | 9 | 5 | 7 | 8 | 3 | 3 | 5 | 3 | 6 | 4 | 10 | 6 |
| Kulczynski | 12 | 11 | 9 | 8 | 7 | 8 | 9 | 7 | 13 | 14 | 12 | 10 | 11 | 11 | 15 | NA | 15 | 12 | 5 | 7 | 10 | 3 | 3 | 5 | 3 | 7 | 6 | 11 | 7 |
| Renkonen | 12 | 11 | 9 | 7 | 6 | 8 | 9 | 7 | 13 | 14 | 12 | 10 | 10 | 10 | 15 | 13 | NA | 11 | 5 | 7 | 9 | 3 | 3 | 5 | 3 | 6 | 4 | 10 | 7 |
| NESS | 11 | 10 | 9 | 9 | 7 | 8 | 9 | 7 | 9 | 10 | 8 | 10 | 11 | 11 | 12 | 9 | 10 | NA | 6 | 8 | 10 | 4 | 4 | 5 | 4 | 6 | 5 | 9 | 8 |
| Gower | 15 | 15 | 14 | 12 | 11 | 11 | 10 | 9 | 12 | 11 | 11 | 14 | 12 | 12 | 12 | 12 | 12 | 12 | NA | 11 | 11 | 8 | 8 | 8 | 8 | 9 | 10 | 10 | 10 |
| Jost Shannon | 11 | 11 | 10 | 11 | 10 | 8 | 10 | 8 | 12 | 14 | 12 | 10 | 12 | 11 | 14 | 14 | 14 | 13 | 6 | NA | 11 | 5 | 4 | 8 | 4 | 8 | 8 | 12 | 8 |
| Jost Simpson | 12 | 11 | 9 | 11 | 9 | 10 | 11 | 9 | 12 | 13 | 11 | 10 | 13 | 12 | 14 | 12 | 13 | 11 | 6 | 9 | NA | 3 | 4 | 5 | 4 | 6 | 5 | 9 | 8 |
| Euclidean | 14 | 14 | 13 | 14 | 14 | 13 | 14 | 12 | 13 | 14 | 12 | 13 | 15 | 15 | 15 | 15 | 15 | 15 | 11 | 13 | 15 | NA | 11 | 13 | 13 | 13 | 12 | 15 | 14 |
| Av. Euclidean | 14 | 14 | 14 | 13 | 13 | 13 | 14 | 12 | 13 | 14 | 12 | 14 | 14 | 14 | 15 | 15 | 15 | 13 | 11 | 12 | 14 | 10 | NA | 12 | 14 | 14 | 11 | 14 | 12 |
| Manhattan | 13 | 13 | 13 | 13 | 12 | 11 | 13 | 11 | 13 | 15 | 13 | 12 | 14 | 14 | 15 | 15 | 15 | 14 | 9 | 12 | 13 | 8 | 7 | NA | 8 | 12 | 13 | 15 | 11 |
| alt. Gower | 14 | 14 | 13 | 12 | 11 | 12 | 13 | 11 | 13 | 14 | 12 | 13 | 14 | 14 | 15 | 15 | 15 | 13 | 11 | 12 | 14 | 8 | 9 | 11 | NA | 13 | 11 | 14 | 12 |
| CYd | 14 | 14 | 13 | 12 | 12 | 12 | 13 | 11 | 12 | 13 | 11 | 11 | 14 | 14 | 14 | 13 | 14 | 13 | 8 | 10 | 14 | 6 | 5 | 7 | 6 | NA | 9 | 12 | 10 |
| Binomial | 14 | 13 | 12 | 13 | 12 | 11 | 13 | 11 | 12 | 14 | 12 | 12 | 14 | 14 | 14 | 12 | 14 | 14 | 8 | 12 | 13 | 9 | 8 | 10 | 8 | 10 | NA | 13 | 11 |
| Lande Shannon | 11 | 10 | 10 | 10 | 9 | 9 | 10 | 8 | 12 | 13 | 11 | 11 | 12 | 12 | 15 | 13 | 14 | 12 | 7 | 9 | 11 | 6 | 5 | 8 | 5 | 9 | 8 | NA | 10 |
| Lande Simpson | 11 | 10 | 10 | 11 | 10 | 11 | 12 | 10 | 11 | 12 | 10 | 11 | 14 | 13 | 13 | 12 | 12 | 12 | 8 | 11 | 11 | 8 | 8 | 9 | 8 | 10 | 9 | 12 | NA |

**References**

Anderson, M.J., Ellingsen, K.E. & McArdle, B.H. (2006). Multivariate dispersion as a measure of beta diversity. *Ecology Letters*, **9**, 683–93.

Anderson, M.J. & Millar, R.B. (2004). Spatial variation and effects of habitat on temperate reef fish assemblages in northeastern New Zealand. *Journal of Experimental Marine Biology and Ecology*, **305**, 191–221.

Baselga, A. (2013). Separating the two components of abundance-based dissimilarity: balanced changes in abundance vs. abundance gradients. *Methods in Ecology and Evolution*, **4**, 552–557.

Bray, J.R. & Curtis, J.T. (1957). An Ordination of the upland forest communities of southern Wisconsin. *Ecological Monographs*, **27**, 326–349.

Cao, Y., Williams, W.P. & Bark, A.W. (1997). Similarity Measure Bias in River Benthic Auswuchs Community Analysis. *Water Environment Research*, **69**, 95–106.

Chao, A., Chazdon, R.L., Colwell, R.K. & Shen, T.-J. (2005). A new statistical approach for assessing similarity of species composition with incidence and abundance data. *Ecology Letters*, **8**, 148–159.

Clifford, H.T. & Stephenson, W. (1975). *An Introduction to Numerical Classification*. Academic Press, New York.

Gower, J.C. (1971). A General coefficient of similarity and some of its properties. *Biometrics*, **27**, 857–871.

Grassle, J.F. & Smith, W. (1976). A Similarity Measure Sensitive to the Contribution of Rare Species and Its Use in Investigation of Variation in Marine Benthic Communities. *Oecologia*, **25**, 13–22.

Horn, H.S. (1966). Measurement of overlap in comparative ecological studies. *American Naturalist*, **100**, 419–424.

Jaccard, P. (1912). The distribution of the flora in the alpine zone. *New Phytologist*, **11**, 37–50.

Jost, L. (2006). Entropy and diversity. *Oikos*, **113**, 363–375.

Jost, L. (2007). Partitioning diversity into independent alpha and beta components. *Ecology*, **88**, 2427–2439.

Koleff, P., Gaston, K.J. & Lennon, J.J. (2003). Measuring beta diversity for presence-absence data. *Journal of Animal Ecology*, **72**, 367–382.

Krebs, C. (1998). *Ecological Methodology*, 2nd ed.n. Harper and Row, New York.

Kulczynski, S. (1927). Die pflanzenassoziationen der Penninen. *Bulletin International de l’Academie Polonaise des Sciences et des Lettres, Classe des sciences mathematiques et naturelles, Serie B*, **Supp 2**, 57–203.

Lance, G.N. & Williams, W.T. (1967). Mixed-data classificatory programs I.) Agglomerative Systems. *Australian Computer Journal*, **1**, 15–20.

Lande, R. (1996). Statistics and partitioning of species diversity, and similarity among multiple communities. *Oikos*, **76**, 5–13.

Legendre, P. (2014). Interpreting the replacement and richness difference components of beta diversity. *Global Ecology and Biogeography*, **23**, 1324–1334.

Lennon, J.J., Koleff, P., Greenwood, J.J.D. & Gaston, K.J. (2001). The geographical structure of British bird distributions: diversity, spatial turnover and scale. *Journal of Animal Ecology*, **70**, 966–979.

Morisita, M. (1959). Measuring of interspecific association and similarity between communities. *Memoirs of the Faculty of Science, Kyushu University, Series E: Biology*, **3**, 65–80.

Podani, J., Ricotta, C. & Schmera, D. (2013). A general framework for analyzing beta diversity, nestedness and related community-level phenomena based on abundance data. *Ecological Complexity*, **15**, 52–61.

Renkonen, O. (1938). Statisch-ökologische Untersuchungen über die terrestrische Käferwelt der finnischen Bruchmoore. *Annales Zoologici Societatis Zoologicæ-Botanicæ Fennicæ Vanamo*, **6**, 1–231.

Ružička, M. (1958). Anwendung mathematisch-statistischer Methoden in der Geobotanik (Synthetische Bearbeitung von Aufnahmen). *Biologia, Bratislava*, **13**, 647–661.

Shannon, C.E. (1948). A mathemtical theory of communication. *Bell System Technical Journal*, **27**, 379–423.

Simpson, E.H. (1949). Measurement of Diversity. *Nature*, **163**, 688.

Sørensen, T.A. (1948). A method of establishing groups of equal amplitude in plant sociology based on similarity of species content, and its application to analyses of the vegetation on Danish commons. *Kongelige Danske Videnskabernes Selskabs Biologiske Skrifter*, **5**, 1–34.
